# Temporal pattern of loss/persistence of duplicate genes involved in signal transduction and metabolic pathways after teleost-specific genome duplication

**DOI:** 10.1186/1471-2148-9-127

**Published:** 2009-06-05

**Authors:** Yukuto Sato, Yasuyuki Hashiguchi, Mutsumi Nishida

**Affiliations:** 1 Division of Molecular Marine Biology, Ocean Research Institute, The University of Tokyo, 1-15-1 Minamidai, Nakano-ku, Tokyo 164-8639, Japan; 2 Division of Population Genetics, National Institute of Genetics, 1111 Yata, Mishima, Shizuoka 411-8540, Japan

## Abstract

**Background:**

Recent genomic studies have revealed a teleost-specific third-round whole genome duplication (3R-WGD) event occurred in a common ancestor of teleost fishes. However, it is unclear how the genes duplicated in this event were lost or persisted during the diversification of teleosts, and therefore, how many of the duplicated genes contribute to the genetic differences among teleosts. This subject is also important for understanding the process of vertebrate evolution through WGD events. We applied a comparative evolutionary approach to this question by focusing on the genes involved in long-term potentiation, taste and olfactory transduction, and the tricarboxylic acid cycle, based on the whole genome sequences of four teleosts; zebrafish, medaka, stickleback, and green spotted puffer fish.

**Results:**

We applied a state-of-the-art method of maximum-likelihood phylogenetic inference and conserved synteny analyses to each of 130 genes involved in the above biological systems of human. These analyses identified 116 orthologous gene groups between teleosts and tetrapods, and 45 pairs of 3R-WGD-derived duplicate genes among them. This suggests that more than half [(45×2)/(116+45)] = 56.5%) of the loci, probably more than ten thousand genes, present in a common ancestor of the four teleosts were still duplicated after the 3R-WGD. The estimated temporal pattern of gene loss suggested that, after the 3R-WGD, many (71/116) of the duplicated genes were rapidly lost during the initial 75 million years (MY), whereas on average more than half (27.3/45) of the duplicated genes remaining in the ancestor of the four teleosts (45/116) have persisted for about 275 MY. The 3R-WGD-derived duplicates that have persisted for a long evolutionary periods of time had significantly larger number of interacting partners and longer length of protein coding sequence, implying that they tend to be more multifunctional than the singletons after the 3R-WGD.

**Conclusion:**

We have shown firstly the temporal pattern of gene loss process after 3R-WGD on the basis of teleost phylogeny and divergence time frameworks. The 3R-WGD-derived duplicates have not undergone constant exponential decay, suggesting that selection favoured the long-term persistence of a subset of duplicates that tend to be multi-functional. On the basis of these results obtained from the analysis of 116 orthologous gene groups, we propose that more than ten thousand of 3R-WGD-derived duplicates have experienced lineage-specific evolution, that is, the differential sub-/neo-functionalization or secondary loss between lineages, and contributed to teleost diversity.

## Background

Whole-genome duplication (WGD) is believed to be one of several major evolutionary events that shaped the genomes of eukaryotes from yeasts and plants to vertebrates [1-6]. WGD, which generates dozens of thousands of duplicate genes, is usually followed by massive gene loss or the acquisition of new roles for the duplicated genes (i.e., sub-/neo-functionalization [7,8]). The evolution of these duplicate genes should occur independently among the lineages that diverged after the WGD. Such an event may contribute to the emergence of genomic differences among lineages that have experienced a WGD, and, therefore increase the genetic and phenotypic diversity among the organisms in that group.

Jawed vertebrates, which have the most complex body plan and behavioral characteristics, are thought to have experienced two rounds of (1R- and 2R-) WGD events early in their evolution, and teleost fishes experienced one more WGD (3R-WGD [6,9-12]). This notion is supported by data from several recent genomic analyses [4,13-21]. Since these events (1R-, 2R-, and 3R-WGD) occurred in ancestors shared by major phylogenetic groups such as tetrapods and teleosts, they may have been important for the formation of vertebrate-specific genomic features.

The teleost-specific 3R-WGD may be crucial to understanding certain aspects of teleost diversity; in addition, the event is very interesting given its time of occurrence. The 1R- and 2R-WGDs, which contributed to the formation of jawed-vertebrate genomes, including those of mammals, occurred before the split between tetrapods and teleosts; thus, they are very old events [5,14,15,17]. Probably because of their oldness, the 1R- and 2R-WGDs seem to have left few traces in the duplicated genes or tree topology of the gene families within the current genomes [4,22,23]. On the other hand, the 3R-WGD is estimated to have occurred in an ancestor of teleosts but after the divergence of teleosts and tetrapods [24-26]. Thus, it is the relatively recent WGD shared by a large vertebrate group, i.e., teleosts. Therefore, we can expect that teleost genomes contain many more WGD-derived duplicate genes and their 'traces' of evolution than tetrapod genomes. In addition, in teleosts, whole-genome sequence data from multiple species [16,20,27], reliable phylogenetic frameworks, and estimated divergence times between lineages [28-33] are now available. Thus, we can systematically analyze the evolution of WGD-derived duplicate genes and their lineage-specific actions by focusing on teleosts, which experienced 3R-WGD. This will provide valuable insights into the evolution of vertebrates through WGDs. 

Previous studies have analyzed WGD-derived duplicate genes mainly by pairwise comparisons using the genomes of phylogenetically distant species such as human and pufferfishes [15-17,19,20]. Studies focusing on a particular gene family have also been performed (e.g., [34,35]). Studies on the medaka and green spotted puffer fish (*Tetraodon*) genomes suggested that they contain about 2,000 pairs of duplicate genes derived from the 3R-WGD [20]. The analyses based on pairwise comparison, however, are insufficient to address the detailed evolutionary process, e.g., the temporal- and lineage-specific manner of gene loss/persistence, after the WGD. These concerns are still not fully resolved by studies based on whole genome data from a few teleost species [36,37], and they can be resolved only by ancestral-state inferences by using multiple genome data, and phylogenetic frameworks and divergence time estimations. Thus, it is unknown how duplicated genes have been maintained or lost temporarily through lineage diversification after the WGD and how many of them contribute to the current genomes of each species.

To address these concerns, we estimated the evolutionary processes experienced by the duplicated genes after the 3R-WGD by analyzing the whole-genome data of four teleosts (zebrafish, stickleback, medaka, and *Tetraodon*) based on teleost phylogeny and their divergence time estimates. In our comparative analyses, we focused on signal transduction pathways involved in learning, memory, and sensory perception, which may have played crucial roles in vertebrate evolution, as well as energy metabolism, which is common to eukaryotes. As representatives of each pathway, we selected four molecular interaction networks: long-term potentiation of synaptic transmission (LTP), taste transduction (TT), olfactory transduction (OT), and tricarboxylic acid cycle (TCA). First, we sought duplicate genes derived from the 3R-WGD by intensive phylogenetic and conserved synteny analyses of the genes involved in the above networks. Next, we estimated the temporal pattern of duplicate gene loss/persistence after the 3R-WGD on the basis of teleost phylogeny and their divergence time framework [28,30,31,33]. The 3R-WGD-derived duplicates that have persisted over the course of evolution were analyzed in terms of their function, locations in the network topologies, numbers of interacting partners, and total length of coding sequences.

## Results

### Orthologous gene groups between tetrapods and teleosts

In this study, protein-coding genes involved in LTP, TT, OT, and TCA were analyzed to search for duplicate genes derived from the 3R-WGD, and their subsequent loss or retention was inferred. According to the KEGG pathway database [38], LTP, TT, OT, and TCA (the network schemes are based on knowledge in the human) comprise 67, 24, 32, and 27 human loci, respectively (olfactory receptor [OR] and taste receptor type 2 [T2R] genes were excluded; see Methods). Among these genes, three, eight, and five were repeatedly involved in LTP and TT; LTP and OT; and LTP, TT, and OT, respectively. After removing these overlaps, 130 human genes [see Additional file 1: Table S1] were subjected to a comparative genomic analysis with other animal genomes.

**Figure 1:**
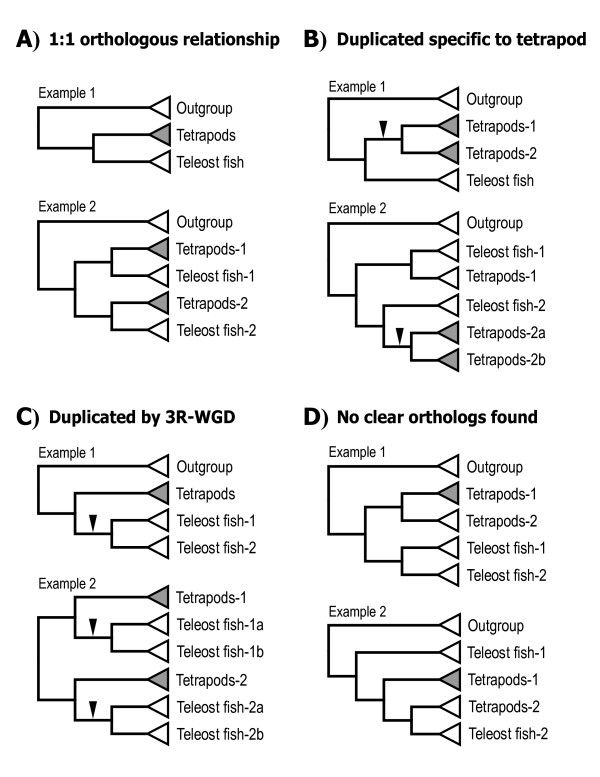
**Schematic view of the four categories of orthologous gene groups**. (A) Gene groups with a 1:1 orthologous relationship between tetrapods and teleost fishes. (B) Gene groups that were duplicated specifically within the human or tetrapod lineage. (C) Gene groups that were duplicated through 3R-WGD. (D) Gene groups with no clear orthologous relationship between tetrapods and teleosts. Arrows denote gene duplication events. Triangles denote the orthologous gene clades derived from tetrapods, teleosts, or outgroup organisms (*Ciona *and *Drosophila*). Shaded triangles denote orthologous gene clades containing a human networks-related gene.

Putative orthologs of these 130 human genes in *Tetraodon*, stickleback, medaka, zebrafish, chicken, clawed frog, ascidian, and fruit fly were obtained through BLASTN searches against their genomes using human protein-coding sequences as queries (for details, see Methods). The obtained genes were subjected to a series of phylogenetic analyses, including preliminary and secondary neighbor-joining (NJ) analyses and final maximum likelihood (ML) analysis. Based on the ML trees [see Additional file 2: Fig. S1–S63], the genes were classified into four categories (Figure 1; for details, see Methods). As a result, 119 of the 130 human genes were found to have orthologs in teleost genomes [see Additional file 2: Fig. S1–S63], while no clear orthologs were identified for the remaining 11 genes (PPP3CB; PPP3R2; CALM1, 2, 3, and 6; PRKCG; GUCA1C; and CLCA1, 2, and 4) [see Additional file 2: Fig. S14, S15, S16, S25, S45, and S47]. Among the 119 human genes, 3 were duplicated specifically in human [see Additional file 2: Fig. S7 and S53B]. Accordingly, we identified a total of 116 orthologous gene groups between tetrapods and teleosts, corresponding to the 130 human genes examined (Table 1).

**Table 1:** Number of orthologous relationships identified between human and teleosts.

	LTP	TT	OT	TCA	Grand total^1^
# of network-related loci in human	67	24	32	28	130
Duplicated specific to tetrapods or human	2	2	2	1	3
No clear orthologs	7	0	8	0	11
# of orthologous relationships identified	58	22	22	27	116

^1^Overlaps between genes that were involved in more than one network were controlled.

*Abbreviations*: LPT, long-term potentiation; TT, taste transduction; OT, olfactory transduction; TCA, tricarboxylic acid cycle

### Persistence of the duplicated genes after the 3R-WGD

Systematic analysis of the gene phylogenies described above successfully identified the duplicated genes derived from the 3R-WGD. These identifications were ascertained based on previously published information related to doubly conserved synteny in the medaka genome [20] shown in [Additional file 2: Fig. S1–S63] and the genome-to-genome conserved synteny information presented in [Additional file 2: Fig. S64–S68]. Duplicated genes derived from the 3R-WGD were identified in at least one species in 45 of the 116 orthologous gene groups (Table 2). This indicates that the genome of the common ancestor of the four teleosts (zebrafish, medaka, stickleback, and *Tetraodon*) contained at least 161 (116+45) loci belonging to the 116 orthologous gene groups, meaning that 56.5% [(45×2)/161] of the loci in the ancestor were duplicate genes derived from the 3R-WGD.

**Table 2:** Number of network-related orthologous gene groups in which gene duplication by the 3R-WGD was detected.

	LTP	TT	OT	TCA	Grand total^1^
Total # of identified relationships	58	22	22	27	116
1:1 orthologs	36	14	10	20	71
Duplicated by 3R-WGD	22	8	12	7	45
3R-WGD-derived duplicate-loci/total gene loci in CA	55.00%	53.30%	70.60%	41.20%	56.50%
	(44/80)	(16/30)	(24/34)	(14/34)	(90/161)
# of 3R-WGD-derived pairs retained in current genome(s)					
One genome	1	1	2	1	5
Two genomes	10	4	5	2	20
Three genomes	7	2	4	3	15
Four genomes	4	1	1	1	5

^1^Overlaps between genes that were involved in more than one network were controlled.

*Abbreviations*: LPT, long-term potentiation; TT, taste transduction; OT, olfactory transduction; TCA, TCA cycle; 3R-WGD, third-round whole genome duplication; CA, common ancestor of zebrafish, medaka, stickleback, and *Tetraodon*.

**Figure 2:**
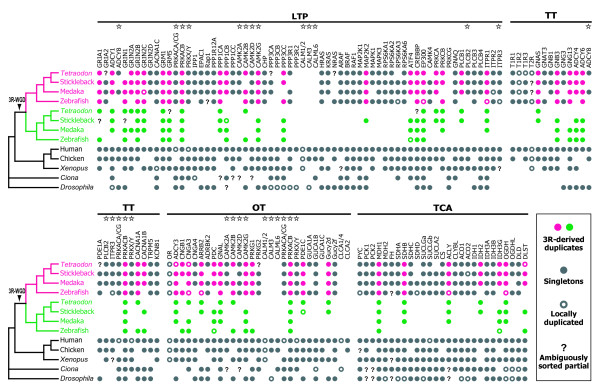
**Schematic representation of the results of comparative genomics-based data mining and maximum likelihood phylogenetic inferences for each gene**. The magenta and green circles denote the presence of genes belonging to the orthologous gene groups in which 3R-WGD-derived duplicates were detected. Gray circles indicate the presence of singleton genes. Open circles denote loci that were specifically duplicated within the species. Question marks indicate the presence of partial gene sequences that are phylogenetically unsorted. Stars denote those gene groups involved in more than one network. Abbreviations: LPT, long-term potentiation; TT, taste transduction; OT, olfactory transduction; TCA, tricarboxylic acid cycle; 3R-WGD, third-round whole genome duplication.

Figure 2 shows a comprehensive graphical representation of the results of our data-mining, phylogenetic, and synteny analyses. Circles indicate the presence of particular genes in the genome of each species, providing information related to lineage-specific loss or the persistence of the 3R-WGD-derived duplicates within the four teleost genomes. For instance, the zebrafish genome is missing one gene for PPP1CA in its LTP network, whereas the other fishes possess both members of the 3R-WGD-derived pair. Based on the lineage-specific presence or absence of the duplicates, we counted the number of 3R-WGD-derived genes and the total number of loci in the four teleost genomes (Table 3). The average of these numbers across the four teleosts shows that, among 133 loci still present on average in a teleost genome, average 55 (55/133 = 41.4%) remain duplicated from the 3R-WGD in at least one teleost genome (not in all four teleost genomes).

**Table 3:** Total number of network-related loci and 3R-WGD-derived duplicated loci identified in each teleost genome.

	LTP		TT		OT	
	# of loci	3R-derived	# of loci	3R-derived	# of loci	3R-derived
CA	58	NA	22	NA	22	NA
*Tetraodon*	66 (68)	26 (26)	24 (27)	6 (6)	29 (31)	16 (18)
Stickleback	71 (74)	34 (35)	27 (34)	12 (12)	27 (33)	16 (22)
Medaka	71 (73)	30 (31)	25 (27)	10 (10)	26 (26)	12 (12)
Zebrafish	55 (61)	22 (23)	24 (25)	10 (10)	26 (29)	12 (13)
						
Average	65.8 (69.0)	28.0 (28.8)	25.0 (28.3)	9.5 (9.5)	27.0 (29.8)	14.0 (16.3)
						
		
	TCA		Grand total^1^			
	# of loci	3R-derived	# of loci	3R-derived		
		
CA	27	NA	116	NA		
*Tetraodon*	30 (31)	10 (10)	133 (141)	52 (54)		
Stickleback	33 (33)	16 (16)	144 (162)	72 (79)		
Medaka	31 (31)	10 (10)	136 (140)	56 (57)		
Zebrafish	26 (31)	2 (2)	117 (131)	38 (40)		
						
Average	30.0 (31.5)	9.5 (9.5)	132.5 (143.5)	54.5 (57.5)		

The numerals in parentheses indicate the number of loci in which lineage-specific gene duplications were incorporated.

^1^Overlaps between genes that were involved in more than one network were controlled.

*Abbreviations*: LPT, long-term potentiation; TT, taste transduction; OT, olfactory transduction; TCA, tricarboxylic acid cycle; CA, common ancestor of tetrapods and teleost fishes; 3R, third-round whole genome duplication; NA, not applicable

A few genes were found to have expanded dramatically by repeated duplication independent of WGD. For example, in the OT system, phosphodiesterase 1C (PDE1C) genes were duplicated specifically in the stickleback genome [see Additional file 2: Fig. S44], implying the existence of multiple bypass circuits that regulate the cyclic AMP (cAMP) pathway in the stickleback OT system. Other examples include the calmodulin (CaM or CALM) genes involved in LTP and OT. The CaM gene family included diverse members before the split of tetrapods and teleosts, and the differential members have persisted in the tetrapod and teleost genomes, respectively [see Additional file 2: Fig. S16]. Interestingly, the amino acid sequences of CALM1 and 2 in tetrapods and their closest CALMs in teleosts are identical in their alignable region.

### Temporal loss or persistence of the duplicated genes after the 3R-WGD

**Figure 3:**
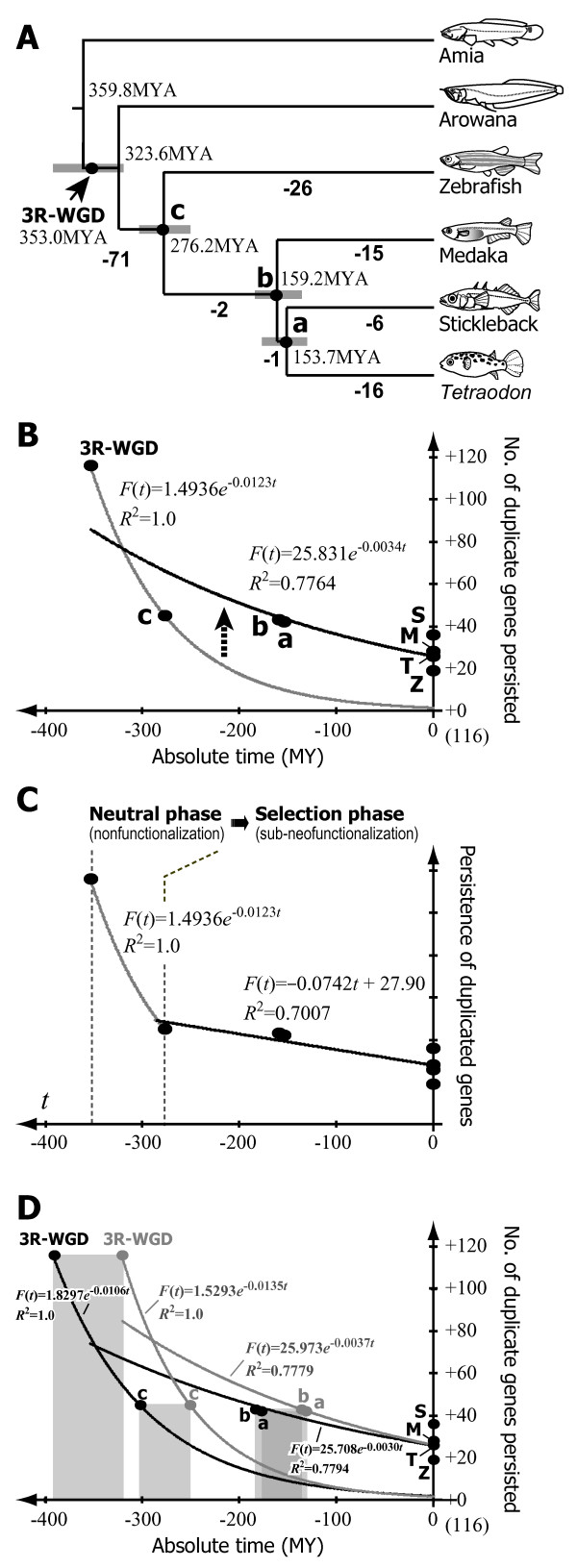
**Inferred temporal process of gene loss after 3R-WGD in a teleost ancestor**. (A) The estimated numbers of gene loss events in the teleost phylogeny was parsimoniously drawn from the presence or absence of loci belonging to the 116 orthologous gene groups. The branch lengths are proportional to the estimated divergence time among the lineages [33]. Gray bars indicate 95% confidence intervals for them. (B) Approximation of the number of gene loss events based on a neutral model of the loss-of-function of duplicated genes (α*e*^-2*μt*^) [8,65,66]. Gray and black lines show the approximation from 3R-WGD and point c, and all data points, respectively. (C) Proposed phase transition during duplicate gene loss. All data points except the 3R-WGD were approximated by a linear equation. (D) Approximation of the number of gene loss events to the upper and lower extremes of the 95% confidence intervals of estimated occurrence time of the 3R-WGD [15,17,19] and divergence times of teleosts [33]. The approximations to the upper and lower extremes are shown by black and gray lines, respectively.

Based on our data concerning the presence or absence of 3R-WGD-derived duplicate loci (Figure 2), we estimated the number of gene loss events on the basis of the teleost phylogeny using parsimony (for details, see Methods). The inferred gene loss events were then assigned to the phylogenetic tree with branch lengths proportional to the estimated divergence time derived from a molecular clock analysis of mitochondrial genome sequences [33] (Figure 3A).

The obtained temporal pattern of gene loss (Figure 3B) indicates that many (71/116 genes; see Table 2) of the duplicated genes were rapidly lost following the 3R-WGD before the split of zebrafish (node *c*), whereas on average more than half (27.3/45 genes; see Table 3 "Grand total": 54.5/2 = 27.3) of the duplicated genes remaining in the ancestor of the four teleosts (45/116 genes) have persisted for about 275 million years (MY). Least-squares fitting of a neutral model of the loss of gene function to the data points for the 3R-WGD and the common ancestor yielded an exponential decay curve with a slope of -0.0123 (Figure 3B gray line). On the other hand, approximation to all data points yielded a moderate curve with slope = -0.0034 (Figure 3B black line), showing that the tempo of gene loss was not constant over the course of evolution after the 3R-WGD (Figure 3C). This result was essentially unchanged when we used upper and lower extremes of the 95% confidence intervals of the estimated times of 3R-WGD and lineage divergences in the approximation (relevant slopes were -0.0106 and -0.0030, and -0.0135 and -0.0037 when upper and lower extreme values were used, respectively; see Figure 3D).

### Properties of the gene groups that retained 3R-WGD-derived duplicates

**Figure 4:**
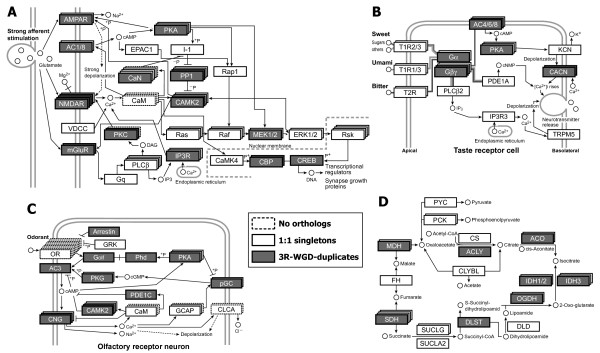
**Distributions of the orthologous gene groups containing duplicate genes that arose through 3R-WGD (shown as gray boxes) in the network diagrams**. Panels A-D: long-term potentiation (LTP), taste transduction (TT), olfactory transduction (OT), and tricarboxylic acid cycle (TCA), respectively. White boxes denote orthologous gene groups with no 3R-WGD-derived duplicates (1:1 orthologous relationship between tetrapods and teleosts). Dashed boxes denote gene groups in which no clear orthologous relationship was identified between tetrapods and teleosts. The network schemes of the LTP, TT, OT, and TCA are based on knowledge in human summarized in KEGG pathway database [38].

We characterized the 3R-WGD-derived duplicate genes on the basis of several properties of the encoded proteins. In terms of their locations in the network topologies of LTP, TT, OT, and TCA, we were not able to find a local concentration of 3R-WGD-derived duplicates (e.g., their localization at upstream or downstream of the pathways; Figure 4). In addition, although most of the proteins included in the four systems are of different types, we found no significant difference in the frequency of 3R-WGD-derived duplicates (described in Table 2) among the four networks (χ^2 ^= 1.7266, d.f. = 3, *P *= 0.6310).

**Figure 5:**
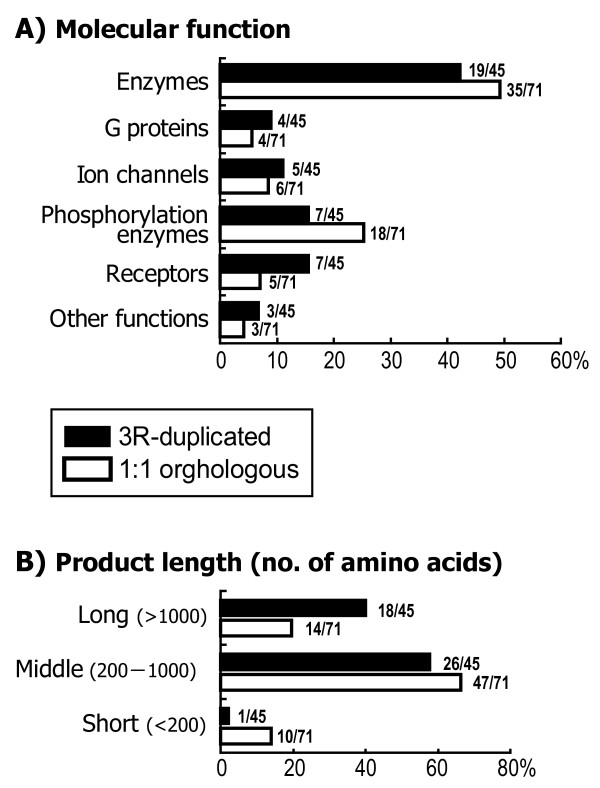
**Characteristics of the orthologous gene groups in which duplicated genes generated by the 3R-WGD were detected (black bar) compared to the groups in which 3R-WGD-derived duplicate genes were not detected (white bar)**. (A) Frequencies of six types of protein function. (B) Frequency of each of three classes of protein-coding region length (# of amino acids).

We next compared the function, total length, and number of interacting partners of the proteins in the networks. There was no significant difference in the frequency of each type of function (e.g., enzymes, G proteins, ion channels, phosphorylation enzymes, and receptors) between the 3R-WGD-derived duplicate genes and 1:1 orthologous genes (χ^2 ^= 4.3984, d.f. = 5, *P *= 0.4936; Figure 5A). However, there was a significant difference in the length of the protein-coding sequences. The protein peptides encoded by the 3R-WGD-derived duplicate genes tended to be long (>1000 amino acids) rather than short (<200 amino acids; χ^2 ^= 8.5044, d.f. = 2, *P *= 0.0142; Figure 5B; human protein were used as the hypothetical ancestral state). Additionally, the 3R-WGD-derived duplicates were enriched in longer-sized glutamate receptors (GRM1, GRM5, GRIN2A, GRIN2B, and GRIN2C) compared to the 1:1 orthologous genes, although this trend was not statistically significant (χ^2 ^= 2.2011, d.f. = 2, *P *= 0.3327). The average number of interacting partners was also significantly higher for the 3R-WGD-derived duplicate genes (*n*_1 _= 71, *n*_2 _= 45, Welch's *t *= 2.0203, *P *= 0.0470) [see Additional file 1: Table S2]. Within the 3R-WGD-derived duplicates, there were no significant differences in the length of protein-coding sequences and the number of interacting partners among the genes that remain duplicated in one, two, three, and four teleost genomes (see Table 2; data not shown).

## Discussion

### Persistence of the duplicated genes after the WGD event

Careful analysis of more than 100 orthologous gene groups in the tetrapod and teleost genomes suggested that the genome of a common ancestor of four teleosts (zebrafish, medaka, stickleback, and *Tetraodon*) contained a high proportion (90/161 = 56.5%) of duplicated genes derived from the teleost-specific 3R-WGD (Table 2). This estimate was produced by extensive data mining of whole-genome databases and reliable ML phylogenetic analyses of the genes involved in LTP, TT, OT, and TCA. Although these genes represent small fraction of the whole genome, and such transcriptional, signal-transduction, and metabolic networks may have different biases for duplicate gene preservation [21,37], we found no significant difference in the frequency of 3R-WGD-derived duplicates among the four networks (see Results and Table 2). This may imply that there is no remarkable difference in retention rate of the 3R-WGD-derived duplicates between subcellular networks that would be important in vertebrate evolution, and metabolism networks that are common to eukaryotes. A possible reason for this is the relatively smaller population size of vertebrates, in which the neutral, random genetic drift is more dominant factor in their duplicate gene loss/retention [8]. In the monocellular eukaryotes, which have much larger population sizes, on the other hand, natural selection would play a more dominant role in duplicate gene loss/retention. Actually, in a ciliate *Paramecium *study, it is suggested that gene dosage constraints (a form of natural selection) after WGD favoured co-retention of the genes within same metabolic pathways or protein complexes [39].

If the retention ratio of 56.5% represents a trend in the whole genome, the duplicate genes generated by the 3R-WGD may have occupied more than half of the protein-coding loci in a common ancestor of the four teleosts. This may even underestimate the true proportion of the 3R-WGD-derived duplicates in the common ancestor, since some duplicates may have been lost independently in all 4 species. In any case, such a large number of duplicate loci, probably more than ten thousand genes, may have undergone lineage-specific loss or differential functionalization among teleosts after their diversification. Such lineage-specific gene evolution would characterize the genome of each teleost lineage, and consequently, contribute to various diversity among modern teleosts, which include more than 26,000 species, 500 families, and 40 orders [40].

Our results also suggests that many of the 3R-WGD-derived duplicate genes persist in modern teleost genomes. The number of the duplicates identified (Table 3) suggests that they comprise on average 41.4% of the protein-coding loci in modern teleosts. This value is higher than those reported in previous studies based on pairwise comparisons of the human and teleost genomes, i.e., 15.2% [(2134×2)/28005] to 20.0% [(2009×2)/20131] [20,41]. These estimations are based on analysis of much larger number of genes compared to those of this study, however, these might be underestimations for the retention rate of 3R-WGD-derived duplicates due to the limited sensitivity of semi-automatic BLAST-based analyses. In our study, we simultaneously analyzed multiple teleost genomes using state-of-the-art methods for phylogenetic inference. Thus, we may have more effectively identified the 3R-WGD-derived duplicates, but instead, our analysis covers limited fraction of the genomes (116 orthologous genes; about 0.5% of all protein-coding genes).

Although teleost genomes may contain large number of 3R-WGD-derived duplicate genes as suggested above, it is reported that teleost and tetrapod genomes have similar number of protein-coding genes in general (mammals: 22,000 on average in human, mouse, dog, and cow; teleosts: 23,000 on average in *Tetraodon*, stickleback, medaka, and zebrafish) [41]. This may be explained by both an increase of gene number in tetrapods through repeated chromosomal rearrangements and local gene duplications [16,20], and secondary loss of 1R- and 2R-WGD-derived duplicates in teleosts [37]. These issues should be addressed within the broader context of 1R/2R/3R-WGDs in early vertebrate evolution based on the emerging genome sequence data of shark (elephantfish), lamprey, lancelet, and sea urchin in the future.

### Evolution of the duplicate genes derived from the 3R-WGD

The inferred temporal pattern of loss/persistence of the 3R-WGD-derived duplicates (Figure 3) provides new insights into the long-term evolution of duplicate genes after WGD. Whereas many (71/116 genes) of the duplicated genes are estimated to have been lost during the initial 75 MY before the divergence of Otocephala (the group including zebrafish; Figure 3B gray line), on average more than half (27.3/45 genes) of the genes survived until the divergence of Otocephala (45/116 genes) persisted for 275 MY (Figure 3B black line). Such a temporal pattern of duplicate gene maintenance cannot be solely explained by a complete neutral loss of gene function, which would be expected to lead to an exponential drop in gene number over time (Figure 3B gray line). Instead, it appears that the genes which persisted for 275 MY have been maintained by selection. This evolutionary scenario is essentially unchanged if we use both upper and lower extremes of 95% confidence intervals for estimated times of the 3R-WGD and lineage divergences (see Figure 3D). Our results, however, are based on a subset of genes in the genomes of only four teleosts and an approximate divergence times of teleosts with large error intervals of 50–100 MYA [33]. In addition, our results might be affected by incompleteness of the draft genome data of teleosts, especially that of zebrafish. Therefore, extrapolating our model proposed here to genome evolution in 26,000 teleost species over 300 MYA should be verified in further studies with an increased number of sample genes and genomes.

The long-term maintenance of the duplicates may be attributable to sub-/neo-functionalization of their gene functions [8,42-44] in general, although advantageous effect of the increase of gene dosage by duplication may also contribute to the duplicate gene persistence [45]. The model of subfunctionalization predicts that, initially after the WGD, many of the duplicated genes were rapidly lost due to the neutral accumulation of degenerative mutations leading to a loss of gene function (neutral phase; Figure 3C); however, a subset of duplicate genes were maintained through subfunctionalization caused by degenerative mutations leading to the loss of a subset of gene functions. Such subfunctionalized duplicates can persist for extremely long (evolutionary) periods of time by selection (selection phase; Figure 3C) and thus afford opportunities for the evolution of new genes with novel functions or more adaptive properties (sub-neofunctionalization [26,43,44]). The proposed time course of gene loss/persistence after the 3R-WGD (Figure 3) seems to be well-matched with the model of sub-neofunctionalization.

The above view is supported by the present results of several analyses related to protein multifunctionality. Because the initial subfunctionalization occurs primarily through degenerative mutations leading to the loss of a subset of gene functions, it is theoretically possible that the probability of subfunctionalization depends on the degree of multifunctionality of the genes or their encoded proteins [8]. This prediction is supported by the observation that the genes included among the 3R-WGD-derived duplicates are significantly longer in terms of their coding sequence (longer sequences likely contain more protein domains and motifs; Figure 5B) and have a significantly larger total number of interacting partners [see Additional file 1: Table S2]. These results, which are compatible with the theoretical prediction of the subfunctionalization model, imply that the 3R-WGD-derived duplicates that remain in modern teleosts have maintained initially by subfunctionalization.

The timing of the occurrences of sub-neofunctionalization is important for interpreting our results and understanding long-term genome evolution after WGDs. If the sub-neofunctionalization have completed mainly in the initial phase after the 3R-WGD but before the divergence of the four teleosts (see Figure 3), this would have contribute little to the differential gene functions among teleost lineages. However, if the neofunctionalization following subfunctionalization occurred mainly after the divergence of the four teleosts, such lineage-specific sub-neofunctionalization of many 3R-WGD-derived duplicates would have contributed to various aspects of teleost diversity. We propose that the latter is the case in general, because neofunctionalization depends on rare beneficial mutations [8] and, thus, this process will proceed gradually over long evolutionary periods of time [26]. These issues, however, should be addressed by further analyses on the spatio-temporal patterns of gene expression and/or repertoires of protein function of the respective 3R-WGD-derived duplicates in the future.

### Genome and gene duplication and teleost evolution

The present study clearly shows that a few gene groups among those analyzed here have expanded dramatically by repeated gene duplications independent of WGD. One representative of them is the well known OR (olfactory receptor) gene family. In vertebrates, this family is constituted by about ten subfamilies [46], and the different subfamily has expanded dramatically in each genome of tetrapods and teleosts, respectively [46,47]. Furthermore, in the OT system, PDE1C genes were found to have been duplicated specifically in stickleback genome [see Additional file 2: Fig. S44]. PDE is a key enzyme in the cyclic AMP pathway, as it regulates the localization, duration, and amplitude of intracellular cAMP signaling [48]. Sticklebacks, therefore, may have multiple bypass circuits to regulate the cAMP pathway possibly in the OT system via sub-/neo-functionalization of these highly duplicated PDE1C genes. These multiple occurrences of the PDE1C genes may be a genetic basis for some adaptive traits of sticklebacks. Recent analysis on the stickleback PDE1C genes has favoured the notion that these multiple duplicates have been retained, not by sub-/neo-functionalization in their gene functions, but rather through their effect of increased gene dosage within the OT system, which is possibly correlated with the territorial behaviour of stickleback [49]. In summary, the above results suggest that both of WGD and lineage-specific expansion of particular genes generated genomic characteristics of each species.

It has been suggested that the 3R-WGD promoted speciation and thus led to an increase in species diversity among teleosts [12,50-54]; however, the association between WGD and species richness in teleosts remains controversial [55]. Even if the 3R-WGD is less directly associated with species richness, we propose that the lineage-specific evolution of many 3R-WGD-derived duplicates, that is, the differential sub-neofunctionalization or secondary loss between lineages, contributed to various aspects of genomic diversity that exists among teleosts. Such diversity may underlie the adaptive radiation of teleosts that inhabit a broad range of ecological zones – from marine to fresh water, from deep to shallow sea environments, and from equatorial to polar regions. To understand the diversity and evolution of teleosts, as well as vertebrates in general, it will be particularly valuable to characterize the differences between teleosts and the basal non-teleost fishes, which have not experienced the 3R-WGD [24-26], in relation to the genetic impact of the 3R-WGD.

## Conclusion

More than 100 genes was systematically analyzed based on whole-genome sequences of multiple teleost fish species (zebrafish, medaka, stickleback, and *Tetraodon*) and state-of-art methods of phylogenetic inference. This revealed that, while many of the duplicate genes derived from the teleost-specific 3R-WGD has been rapidly lost after the 3R-WGD within about 75 MY, the remaining duplicates may have occupied more than half (56.5%) of the protein-coding loci in a common ancestor of the four teleosts. The following temporal pattern of loss/persistence of these duplicates is understood by sub-neofunctionalization model of duplicate gene evolution, and this view is supported by analysis of length of protein-coding sequences and numbers of interaction partner of proteins, which are likely associated with gene multifunctionality. Based on these results, derived from the first systematic assessment of evolutionary fate of more than 100 genes after the 3R-WGD, we propose that many of the 3R-WGD-derived duplicates, probably more than ten thousand of genes, have undergone lineage-specific evolution or secondary loss, and contributed to teleost diversity. Lineage-specific expansion of some portion of gene families also appears to have contributed to genomic characteristics of each lineages.

## Methods

### Identification of orthologous gene groups between tetrapods and teleosts

Network diagrams for LTP, TT, OT, and TCA, and the coding sequences of the human genes that comprise these networks were obtained from the KEGG pathway database [38]. The obtained human coding sequences were used as queries for a BLASTN search against the Ensembl genome database [27,41]. The following versions of the Ensembl genome database were used: human (*Homo sapiens*, NCBI 36, October 2005), chicken (*Gallus gallus*, WASHUC2, May 2006), clawed frog (*Xenopus tropicalis*, JGI 4.1, August 2005), zebrafish (*Danio rerio*, Zv7, April 2007), medaka (*Oryzias latipes*, HdrR, October 2005), stickleback (*Gasterosteus aculeatus*, BROAD S1, February 2006), green spotted puffer fish (*Tetraodon nigroviridis*, TETRAODON 7, April 2003), ascidian (*Ciona intestinalis*, JGI 2, March 2005), and fruit fly (*Drosophila melanogaster*, BDGP 4.3, July 2005). Since the two pufferfishes *Fugu rubripes *and *T. nigroviridis *are much closer to each other than to the other teleosts above, only the latter, for which the genome sequence was determined at relatively high coverage [16], was analyzed. The resulting BLAST hits were manually screened (*E*-value cut-off of < 10^-3^) and evaluated for their gene product length and Ensembl annotations to confirm their similarity to the human gene queries. When only a partial sequence was found in the Ensembl database, we predicted the full-length coding sequence from the genomic sequence using the program WISE2 [56].

The primary sequences of the proteins obtained by the above procedure were aligned using ClustalW [57]. All gap-containing sites were removed. For each alignment, a preliminary NJ analysis was performed based on Poisson-corrected genetic distances using MEGA 3.1 [58]. Based on the resultant NJ trees, those sequences comprising clades, which were apparently distinct from any human query gene, were excluded. The remaining sequences were again analyzed by NJ to identify ingroup and outgroup sequences. The selected primary sequences were then re-aligned and subjected to ML phylogenetic analysis using the program TreeFinder (version June 2007) [59,60] with 1,000 LR-ELW (the expected-likelihood weights applied to local rearrangements of tree topology) edge support tests [61]. In almost all cases in this analysis, the obtained tree topology was consistent between ML and second-ML tree with slight changes in the ML score and supporting values for the nodes of the tree. When the final ML tree was ambiguous, further ML analysis was performed based on nucleotide sequences. The phylogenetic information contained within nucleotide sequences is generally greater than that contained within amino acid sequences, and, in many cases, it increases the resolution of the resulting phylogeny. Such analysis require significant time and effort, and therefore could not be applied to all analyses undertaken in this study. The best-fitting amino acid and nucleotide substitution models were selected using programs ProtTest 1.4 [62] and ModelTest 3.06 [63], respectively.

Based on the final ML trees [see Additional file 2: Fig. S1–S63], the genes were classified into four categories: (*i*) genes with a 1:1 orthologous relationship [64] between human and teleosts (Figure 1A); (*ii*) genes that were duplicated in human or tetrapods but not in teleosts (Figure 1B); (*iii*) 3R-WGD-derived duplicates (Figure 1C); this identification was confirmed by data showing doubly conserved synteny in the medaka genome [20] [indicated in Additional file 2: Fig. S1–S63] and genome-to-genome conserved synteny analyses mentioned below [shown in Additional file 2: Fig. S64–S68]; and (*iv*) genes with no clear orthologous relationship between human and teleosts (Figure 1D). A full list of the genes used in our final ML analysis, including their Ensembl IDs, is given in [Additional file 3: appendix].

### Synteny analysis

To confirm whether the putative 3R-WGD-derived duplicate genes were actually generated by the 3R-WGD, we analyzed conserved syntenies in the medaka genome. In Supplementary Table 15 of the report showing the draft genomic sequence of medaka [20], the authors presented information on conserved syntenies in the medaka genome. Specifically, they determined whether those medaka genes corresponding to 20,352 human protein-coding genes were located in a block of doubly conserved synteny (DCS) derived from the 3R-WGD. Using this information, we confirmed that the inferred teleost-specific duplicate genes were derived from the 3R-WGD. The results of this validation were shown in supplementary figures [see Additional file 2] (the data of gene locations are not shown).

For those teleost-specific duplicate genes that appeared to be derived from the 3R-WGD but were not located on DCSs in medaka, we analyzed the genomic regions around the loci in human, zebrafish, medaka, stickleback, and *Tetraodon*. Physical mapping data in the neighborhood of each locus were obtained via queries and BLAST searches (*E*-value threshold < 10^-3^) using the orthologous prediction section of the Ensembl genome database [27,41]. We picked up all identifiable genes described as putative orthologs of the queries. Their genomic locations were then used to rebuild the synteny maps [see Additional file 2: Fig. S64–S68].

### Analysis of the temporal pattern of gene loss

The numbers of gene loss events after the 3R-WGD was estimated using parsimony based on the teleost phylogeny [28,30,31]. The inferred total number of 3R-WGD-derived duplicate pairs that persisted in each node of the phylogeny was matched with the corresponding divergence time estimate [33]. The derived two-dimensional data points, estimated occurrence time of the 3R-WGD event (average 353.0 MY ago) [15,17,19], and four data points representing the current state of the four teleost genomes were approximated to a neutral model of the loss-of-function of duplicated genes, α*e*^-2*μt *^[8,65,66], using least-squares fitting. The parameters *μ*, *t*, and *α *corresponds to the null mutation rate, number of generations since the time of duplication, and normalization factor, respectively. In this analysis, absolute time (years) was used to approximate the number of generations.

### Estimation of the number of interacting partner of the investigated proteins

The number of interacting partners for the proteins in the LTP, TT, OT, and TCA was counted on the basis of human network diagrams obtained from the KEGG pathway database [38]. In the case of CAMK2B, CAMK2G, PRKACB, CAMK2A, CAMK2D, ADCY8, PLCB2, ITPR3, PRKACA, PRKACG, PRKX, PRKY, CaM, and PKA, those molecular interactions that served essentially the same function were counted as one. For example, adenylate cyclase 8 (ADCY8, EC: 4.6.1.1) is involved in LTP and TT, and it interacts with CaM and cAMP in the former, and with cAMP and G-protein in the latter; however, the total number of interacting partners for ADCY8 was counted as three (CaM, cAMP, and G-proteins). In the case of CaM, although CaM interacts with many of types of proteins, only two interacting partners were counted (Ca ^2+ ^and another protein). This is because Ca ^2+^-activated CaM functions as a calcium sensor and signal transducer for a multitude of proteins. This view is supported by the fact that the amino acid sequences of CaM are extremely conserved among vertebrates [67] while the interacting partners of CaM are numerous. By similar reasoning, the number of interacting partners for PKA was counted as two (cAMP and another protein). All OR genes among the OT and T2R genes in the TT network were excluded from the analysis because the OR and T2R gene families have been enormously expanded by specific duplications in mammals, and the orthologous relationships between tetrapods and teleosts are less clear [46,47,68]. If large numbers of OR and T2R loci were incorporated into the current analysis, the results would be biased and beyond the scope of this study. The numbers loci of the taste receptor type 1 genes (T1R1, T1R2, and T1R3) are as previously reported [69,70].

## List of abbreviations used

3R-WGD: third-round whole genome duplication; LTP: long-term potentiation; TT: taste transduction; OT: olfactory transduction; TCA: tricarboxylic acid cycle; OR: olfactory receptor; T2R: taste receptor type 2; NJ: neighbour joining; ML: maximum likelihood; PPP: protein phosphatase; CALM (gene name) and CaM (protein name): calmodulin; PRK: protein kinase; GUCA: guanylate cyclase activator; CLCA: calcium-dependent chloride channel; MY: million years; cAMP: cyclic adenosine monophosphate; DCS: doubly conserved synteny; CAMK: calcium/calmodulin-dependent protein kinase; ADCY: adenylate cyclase; PLCB: phospholipase C beta; ITPR: inositol 1,4,5-triphosphate receptor; T1R: taste receptor type 1; PKA: protein kinase A: LR-ELW, the expected-likelihood weights applied to local rearrangements of tree topology.

## Authors' contributions

YS, YH, and MN designed the study. YS carried out the analyses and drafted the manuscript. MN participated in coordination and helped to draft the manuscript. All authors read and approved the final version of the manuscript.

## Supplementary Material

Additional file 1 **Supplementary tables**. This PDF file includes supplementary tables S1 and S2. Click here for file

Additional file 2 **Supplementary figures**. This PDF file includes supplementary figures S1 – S68. Click here for file

Additional file 3 **Supplementary appendix**. This PDF file includes an appendix. Click here for file
