# Supplementary material for: Temporal pattern of loss/persistence of duplicate genes involved in signal transduction and metabolic pathways after teleost-specific genome duplication
Source: BMC Evol Biol. 2009 Jun 5;9:127. doi: 10.1186/1471-2148-9-127 (PMC2702319; doi:10.1186/1471-2148-9-127)
Supplement: Additional file 1 — Supplementary tables. This PDF file includes supplementary tables S1 and S2. [file 1471-2148-9-127-S1.pdf]

Table S1 - List of the network-related genes studied.

Abbreviated name: function of molecule	Name(s) of gene locus(loci) in human (related network[s]; # of interacting partners)
AMPAR: glutamate receptor	
	GRIA1 (LTP; 5), GRIA2 (LTP; 5)
AC: adenylate cyclase	
	ADCY1 (LTP; 2), ADCY3 (OT; 4), ADCY4 (TT; 2), ADCY6 (TT; 2), ADCY8 (LTP, TT; 2)
NMDAR: glutamate receptor	
	GRIN1(LTP; 5), GRIN2A (LTP; 5), GRIN2B (LTP; 5), GRIN2C (LTP; 5), GRIN2D (LTP; 5)
VDCC: calcium channel	
	CACNA1C (LTP, 1)
mGluR: glutamate receptor	
	GRM1(LTP; 3), GRM5 (LTP; 3)
PKA: protein kinase A	
	PRKACA (LTP, TT, OT; 2), PRKACB (LTP, TT, OT; 2), PRKACG (LTP, TT, OT; 2), PRKX (LTP, TT, OT; 2), PRKY (LTP, TT, OT; 2)
I-1 or IPP1: protein phosphatase	
	IPP1 (LTP; 3)
EPAC1: guanyl-nucleotide exchange factor	
	EPAC1 (LTP; 2)
Rap1: GTPase	
	Rap1 (LTP; 3)
PP1: protein phosphatase	
	PPP1R12A (LTP; 2), PPP1CA (LTP; 2), PPP1CB (LTP; 2), PPP1CC (LTP; 2)
CaMK2: calcium/calmodulin-dependent protein kinase II	
	CAMK2A (LTP, OT; 3), CAMK2B (LTP, OT; 3), CAMK2D (LTP, OT; 3), CAMK2G (LTP, OT; 3)
CaN: calcium binding/protein phosphatase	
	CHP (LTP; 2), PPP3CA (LTP; 2), PPP3CB (LTP; 2), PPP3CC (LTP; 2), PP3R1 (LTP; 2), PP3R2 (LTP; 2)
CaM: calmodulin	
	CALM1 (LTP, OT; 2), CALM2 (LTP, OT; 2), CALM3 (LTP, OT; 2), CALML6 (LTP, OT; 2)

Ras: GTPase

HRAS (LTP; 2), KRAS (LTP; 2), NRAS (LTP; 2)

Raf: protein kinase

ARAF (LTP; 3), BRAF (LTP; 3), RAF1 (LTP; 3)

MEK1/2: protein kinase kinase

MAP2K1 (LTP; 3), MAP2K2 (LTP; 3)

ERK1/2: protein kinase

MAPK1 (LTP; 3), MAPK3 (LTP; 3)

Rsk: protein serine/threonine kinase

RPS6KA1 (LTP; 2), RPS6KA2 (LTP; 2), RPS6KA3 (LTP; 2), RPS6KA6 (LTP; 2)

CREB: transcription factor

ATF4 (LTP; 3)

CBP: histone acetyltransferase

CREBBP (LTP; 2), EP300 (LTP; 2)

CaMK4: protein kinase

CAMK4 (LTP; 2)

PKC: protein kinase C

PRKCA (LTP; 2), PRKCB (LTP; 2), PRKCG (LTP; 2)

Gq: G protein

GNAQ (LTP; 2)

PLCβ: phospholipase C beta

PLCB1 (LTP; 3), PLCB2 (LTP, TT; 3), PLCB3 (LTP; 3), PLCB4 (LTP; 3)

IPR: inositol 1,4,5-triphosphate receptor

ITPR1 (LTP; 2), ITPR2 (LTP; 2), ITPR3 (LTP, TT; 2)

TAS1R: taste receptor, type 1

T1R1 (TT; 2), T1R2 (TT; 2), T1R3 (TT; 2)

Gα: G protein alpha subunit

GNAS (TT; 2), GNAT3 (TT; 4)

Gβγ: G protein beta and gamma subunit

GNB1 (TT; 4), GNB3 (TT; 4), GNG3 (TT; 4), GNG13 (TT; 4)

PDE1: phosphodiesterase 1

PDE1A (TT; 2), PDE1C (OT; 2)

CACN: calcium channel

CACNA1A (TT; 3), CACNA1B (TT; 3)

TRPM5: potential cation channel

TRPM5 (TT; 2)

KCN: potassium voltage-gated channel

KCNB1 (TT; 3)

CNG: cyclic nucleotide gated channel

CNGB1 (OT; 4), CNGA3 (OT; 4), CNGA4 (OT; 4)

Arrestin

ARRB2 (OT; 1)

GRK: receptor kinase

ADRBK2 (OT; 1)

Phd: phosducin

PDC (OT; 2)

Golf: G protein alpha subunit, olfactory type

GNAL (OT; 3)

PKG: cGMP-dependent protein kinase

PRKG1 (OT; 2), PRKG2 (OT; 2)

GCAP: guanylate cyclase activator

GUCA1A (OT; 2), GUCA1B (OT; 2), GUCA1C (OT; 2)

pGC: guanylate cyclase

Gucy2d (OT; 3), Gucy2f (OT; 3)

CLCA: calcium-dependent chloride channel

CLCA1 (OT; 3), CLCA2 (OT; 3), CLCA4 (OT; 3)

PYC: pyruvate carboxylase

PYC (TCA; 2)

PCK: phosphoenolpyruvate carboxykinase

PCK1 (TCA; 2), PCK2 (TCA; 2)

MDH: L-malate dehydrogenase

MDH1 (TCA; 2), MDH2 (TCA; 2)

FH: fumarate hydratase

FH (TCA; 2)

SDH: succinate dehydrogenase

SDHA (TCA; 2), SDHB (TCA; 2), SDHC (TCA; 2), SDHD (TCA; 2)

SUCLG: succinate-CoA ligase

SUCLG1 (TCA; 2), SUCLG2a (TCA; 2), SUCLG2b (TCA; 2)

SUCLA2: succinate-CoA ligase

SUCLA2 (TCA; 2)

CS: citrate synthase

CS (TCA; 2)

ACLY: ATP citrate synthase

ACLY (TCA; 2)

CLYBL: citrate lyase

CLYBL (TCA; 3)

ACO: aconitase

ACO1 (TCA; 2), ACO2 (TCA; 2)

IDH: isocitrate dehydrogenase (NADP+)

IDH1 (TCA; 2), IDH2 (TCA; 2)

IDH3: isocitrate dehydrogenase (NAD+)

IDH3A (TCA; 2), IDH3B (TCA; 2), IDH3G (TCA; 2)

OGDH: oxoglutarate dehydrogenase

OGDH (TCA; 2), OGDHL (TCA; 2)

DLD: dihydrolipoyl dehydrogenase

DLD (TCA; 2)

DLST: dihydrolipoyllysine-residue succinyltransferase

DLST (TCA; 3)

Abbreviations: LPT, long-term potentiation; TT, taste transduction; OT, olfactory transduction; TCA, tricarboxylic acid cycle

Table S2 - Difference in the number of interacting partners between the 1:1 orthologous and 3R-WGD-derived duplicated gene groups.

Molecular interaction network	# of interaction partners		*P*-value	
	1:1[b]	3R-WGD[c]	Student's *t*	Welch's *t*
LPT	2.51 ± 0.13 (*n*=37)	3.00 ± 0.27 (*n*=21)	0.0732	0.1132
TT	2.62 ± 0.23 (*n*=14)	2.75 ± 0.31 (*n*=8)	0.6460	0.6518
OT	2.40 ± 0.27 (*n*=10)	2.75 ± 0.28 (*n*=12)	0.3810	0.3750
TCA	2.05 ± 0.05 (*n*=19)	2.13 ± 0.13 (*n*=8)	0.5306	0.6058
Grand average[a]	2.41 ± 0.09 (*n*=71)	2.78 ± 0.16 (*n*=45)	*0.0321	*0.0470

*P<0.05

[a]Overlaps between genes that were involved in more than one network were controlled.

[b]Gene groups in which a 1:1 orthologous relationship between tetrapopds and teleost fishes was detected.

[c]Gene groups in which duplicated genes generated through 3R-WGD were detected.

Abbreviations: LPT, long-term potentiation; TT, taste transduction; OT, olfactory transduction; TCA, tricarboxylic acid cycle; 3R-WGD, third-round whole genome duplication
