# Supplementary material for: Temporal pattern of loss/persistence of duplicate genes involved in signal transduction and metabolic pathways after teleost-specific genome duplication
Source: BMC Evol Biol. 2009 Jun 5;9:127. doi: 10.1186/1471-2148-9-127 (PMC2702319; doi:10.1186/1471-2148-9-127)
Supplement: Additional file 2 — Supplementary figures. This PDF file includes supplementary figures S1 – S68. [file 1471-2148-9-127-S2.pdf]

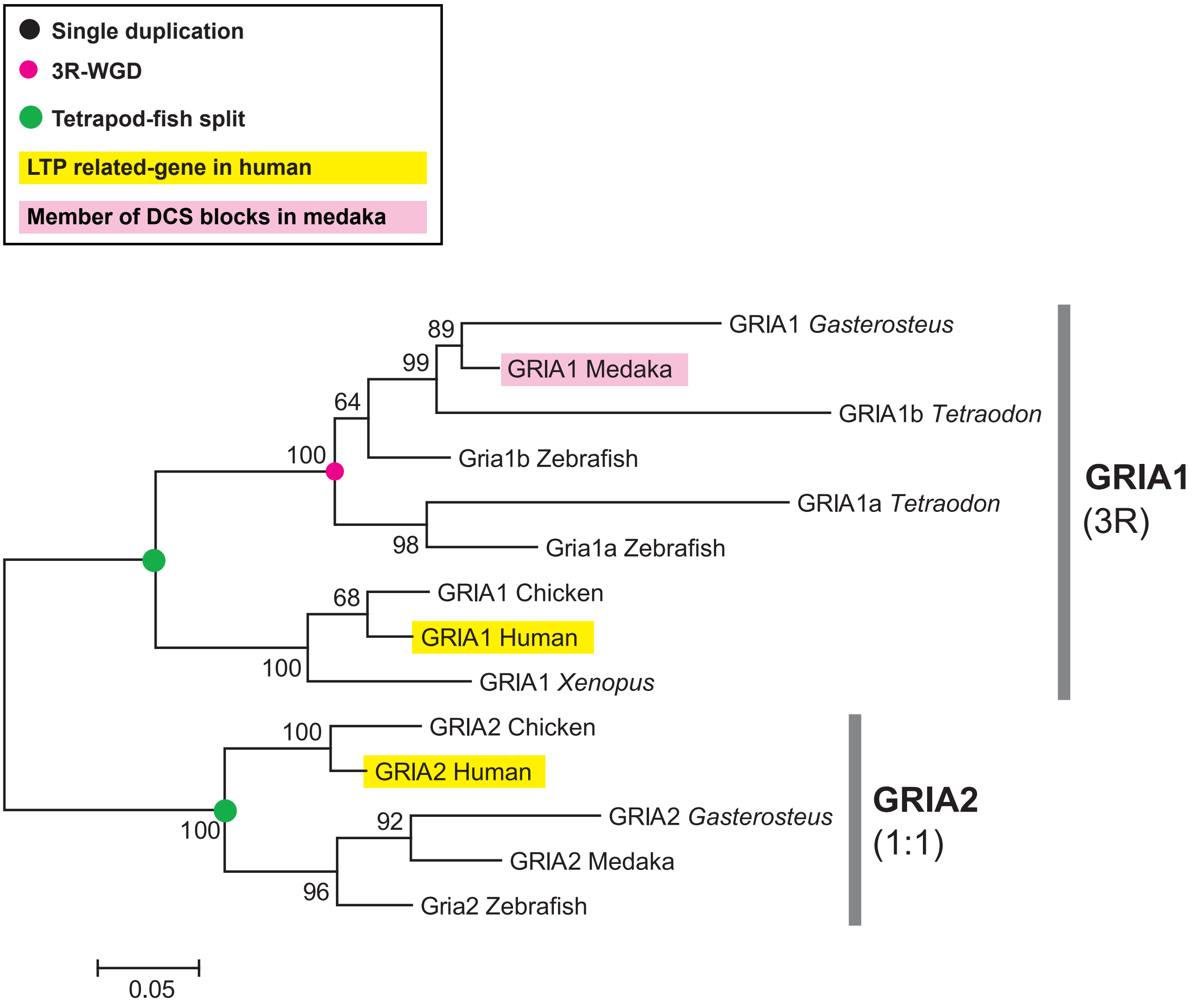

Fig. S1. A molecular phylogeny of AMPAR (glutamate receptor, ionotropic) inferred from maximum-likelihood analysis (606 amino acid sites were used; JTT+Γ). Numbers indicate approximate bootstrap values from 1,000 LR-ELW (the Expected-Likelihood Weights applied to Local Rearrangements of tree topology) tests that support for the nodes.

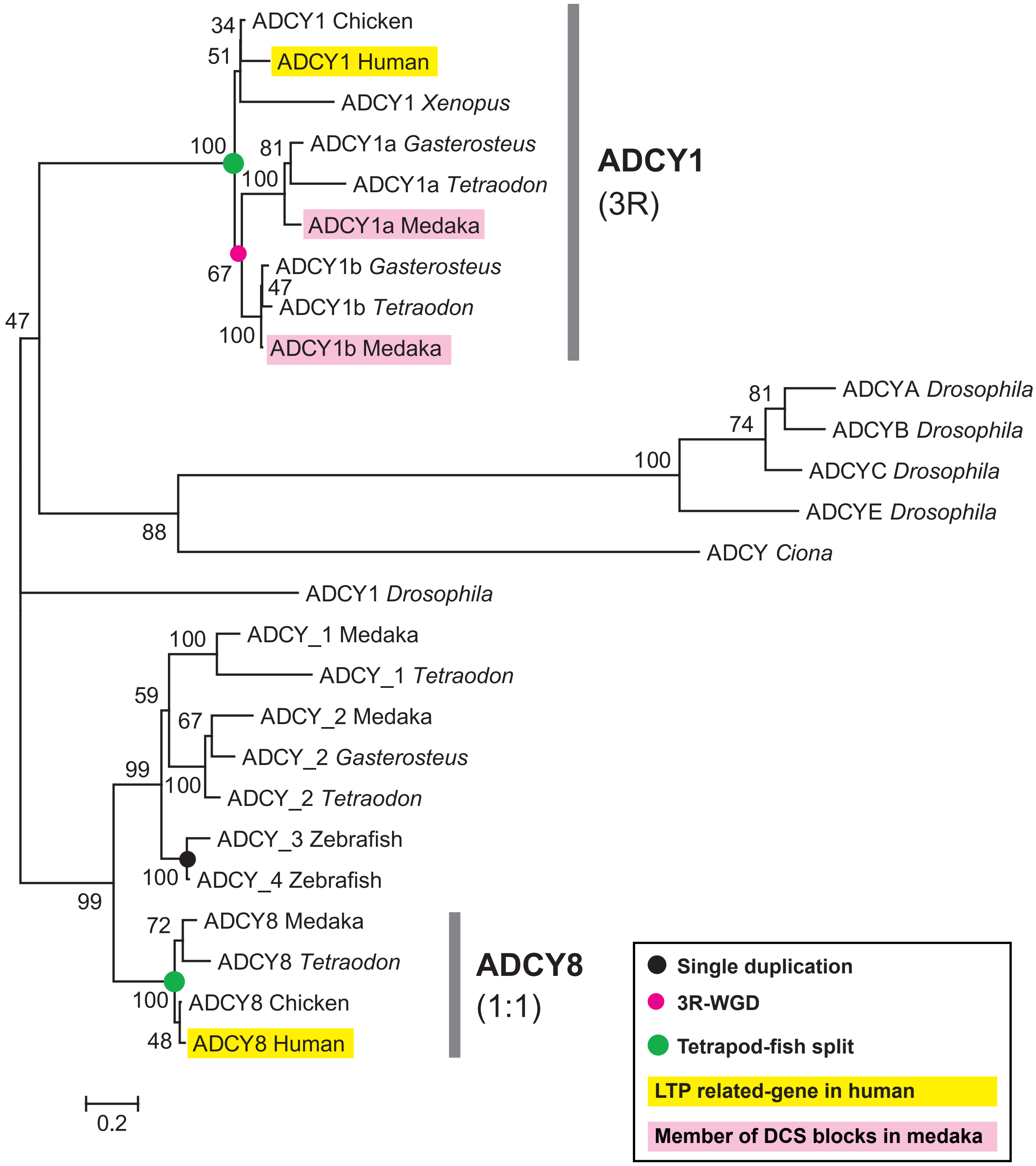

Fig. S2. A molecular phylogeny of AC1 and AC8 (adenylate cyclase 1 and 8, EC: 4.6.1.1) inferred from maximum-likelihood analysis (342 amino acid sites were used; JTT+Γ). Numbers indicate approximate bootstrap values from 1,000 LR-ELW (the Expected-Likelihood Weights applied to Local Rearrangements of tree topology) tests that support for the nodes.

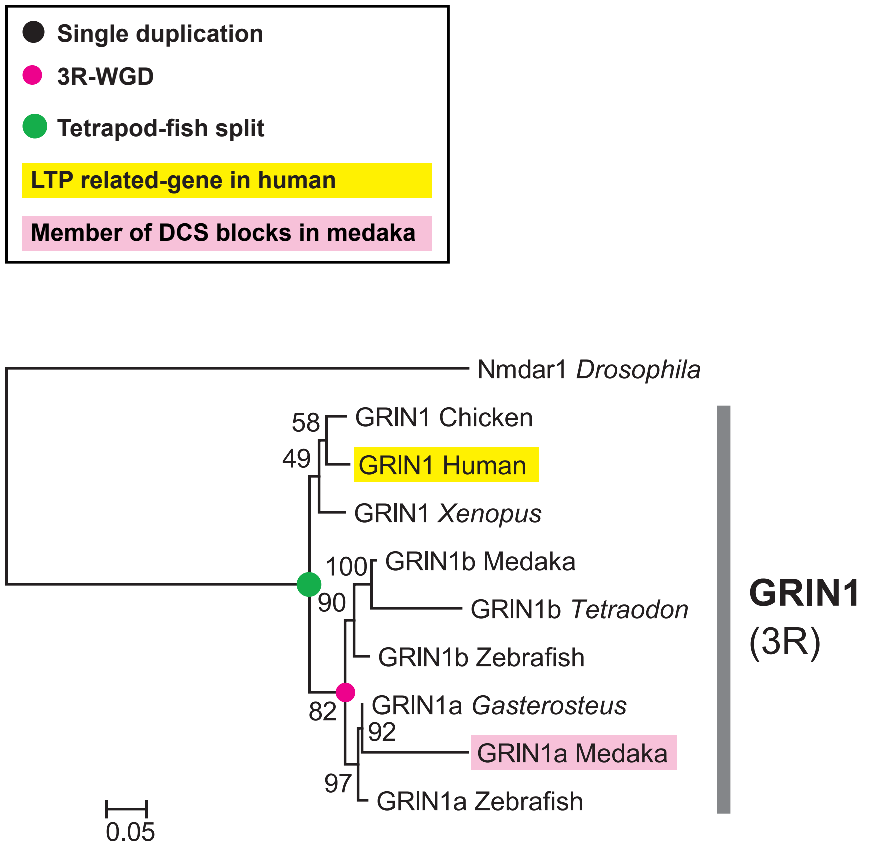

Fig. S3. A molecular phylogeny of NMDAR (glutamate receptor, ionotropic, N-methyl D-aspartate) inferred from maximum-likelihood analysis (752 amino acid sites were used; WAG+Γ). Numbers indicate approximate bootstrap values from 1,000 LR-ELW (the Expected-Likelihood Weights applied to Local Rearrangements of tree topology) tests that support for the nodes.

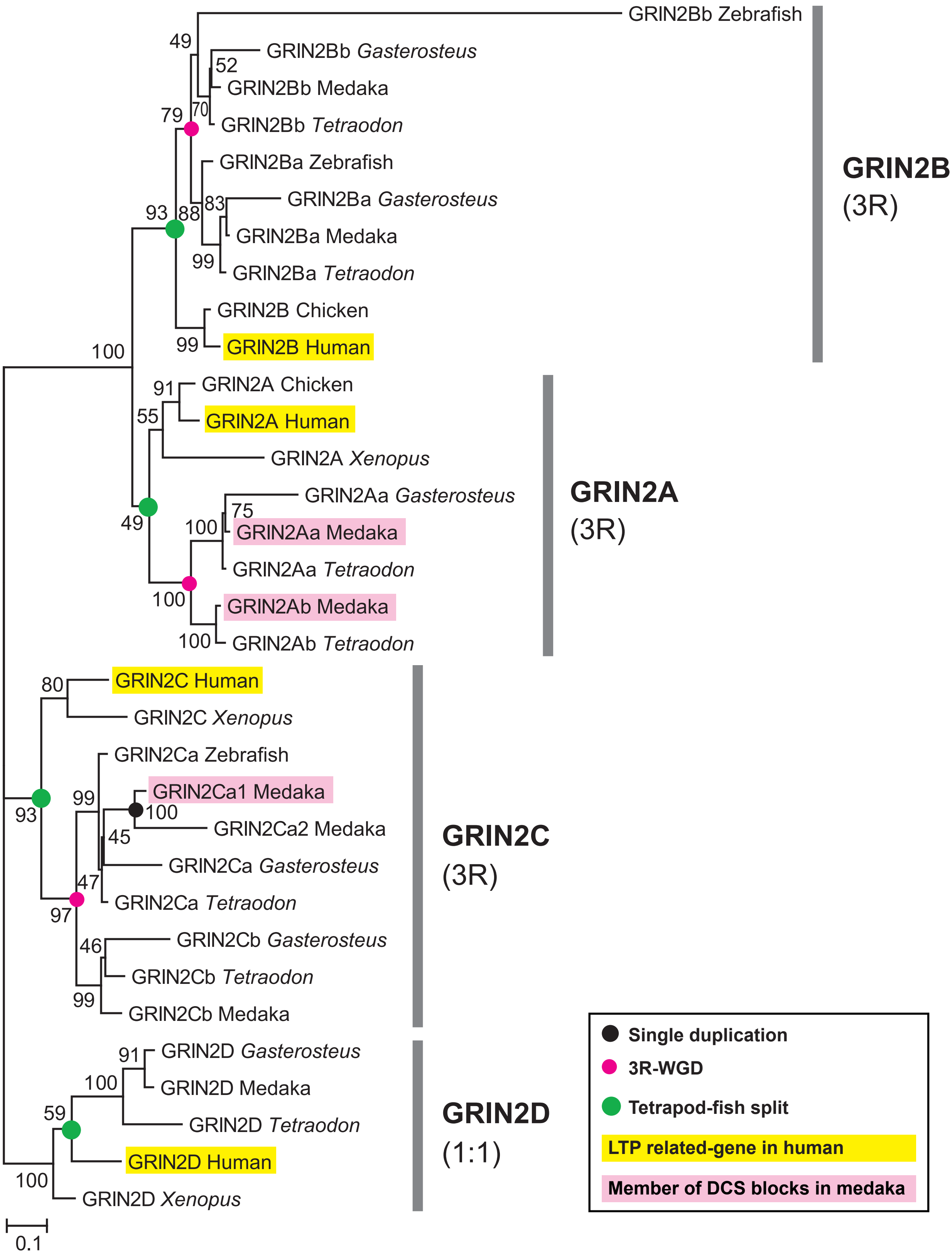

Fig. S4. A molecular phylogeny of NMDAR (glutamate receptor, ionotropic, N-methyl D-aspartate) inferred from maximum-likelihood analysis (321 amino acid sites were used; JTT+Γ). Numbers indicate approximate bootstrap values from 1,000 LR-ELW (the Expected-Likelihood Weights applied to Local Rearrangements of tree topology) tests that support for the nodes.

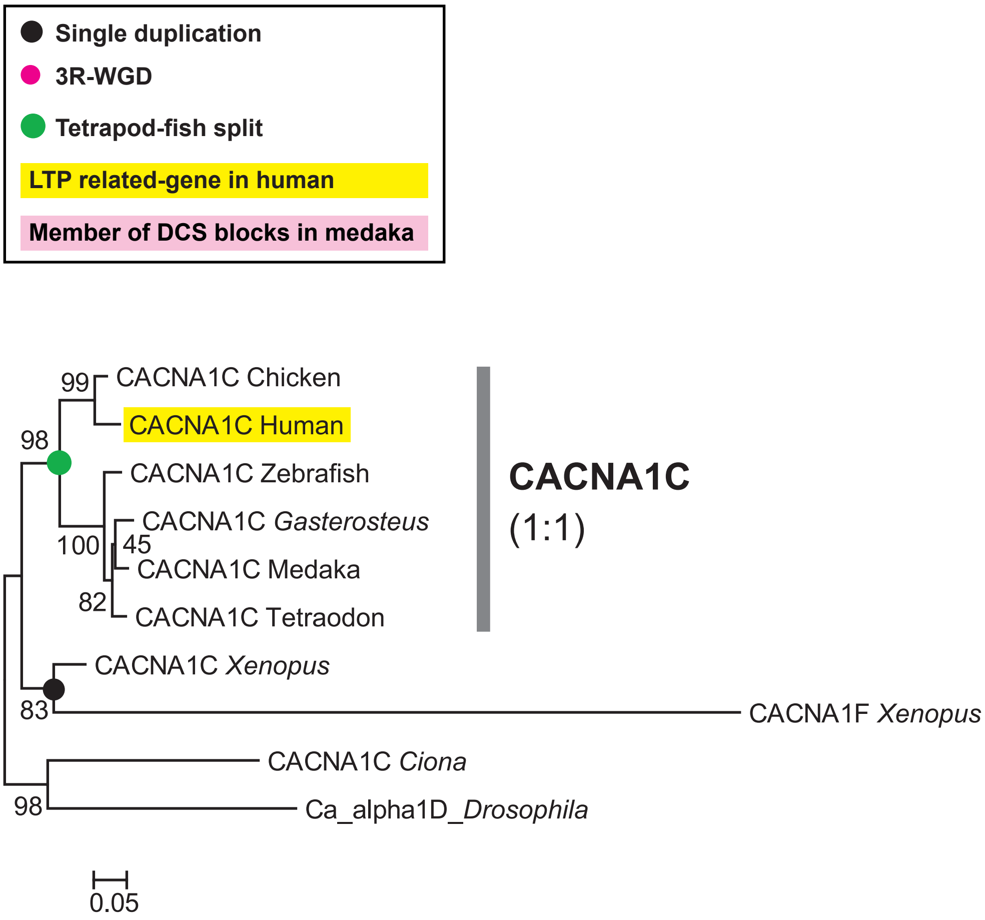

Fig. S5. A molecular phylogeny of VDCC (or CACNA1C, calcium channel, voltage-dependent, L type, alpha 1C subunit), inferred from maximum-likelihood analysis (530 amino acid sites were used; Blosum62+Γ). Numbers indicate approximate bootstrap values from 1,000 LR-ELW (the Expected-Likelihood Weights applied to Local Rearrangements of tree topology) tests

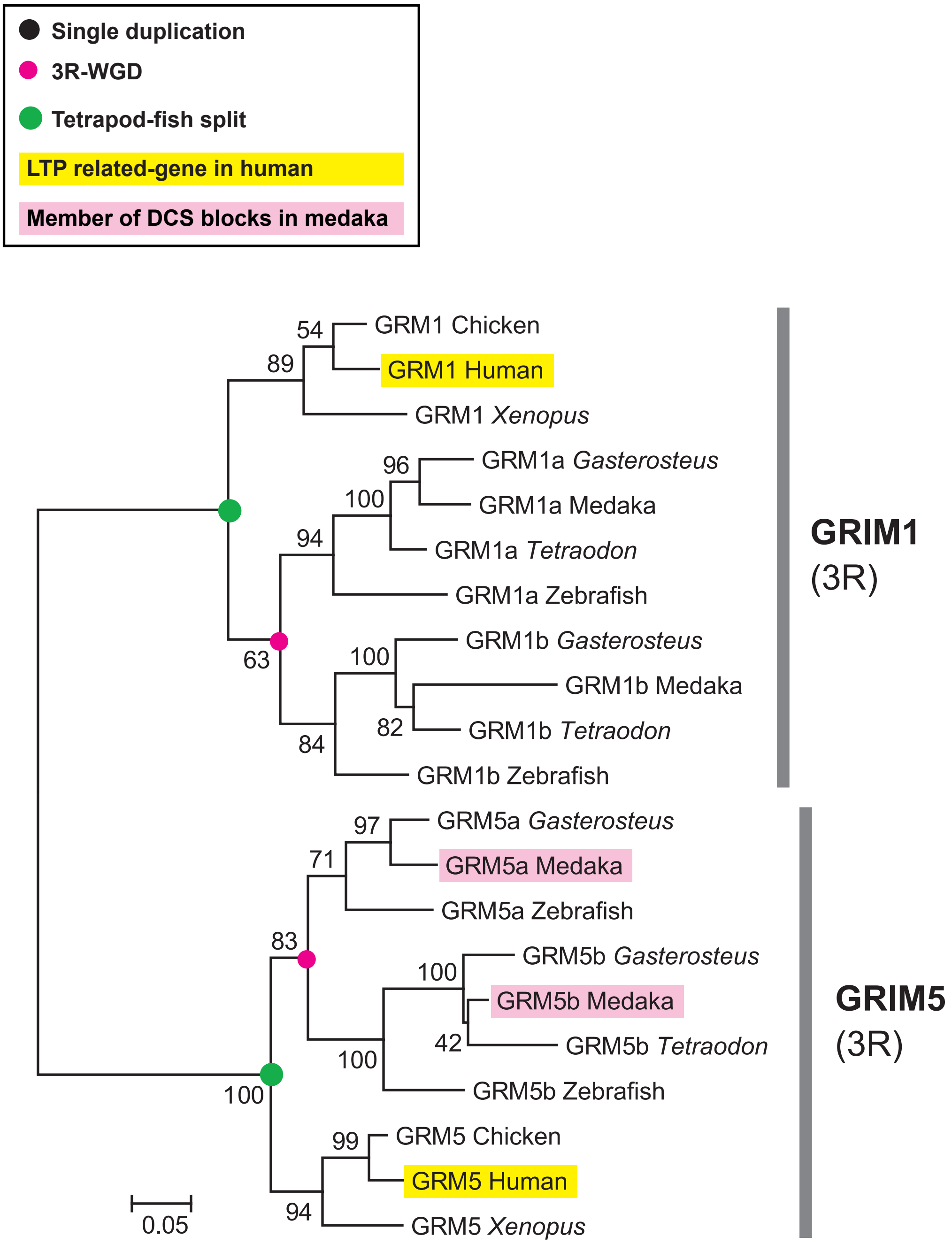

Fig. S6. A molecular phylogeny of mGluR (or GRM, glutamate receptor, metabotropic), inferred from maximum-likelihood analysis (529 amino acid sites were used; JTT+Γ). Numbers indicate approximate bootstrap values from 1,000 LR-ELW (the Expected-Likelihood Weights applied to Local Rearrangements of tree topology) tests that support for the nodes.

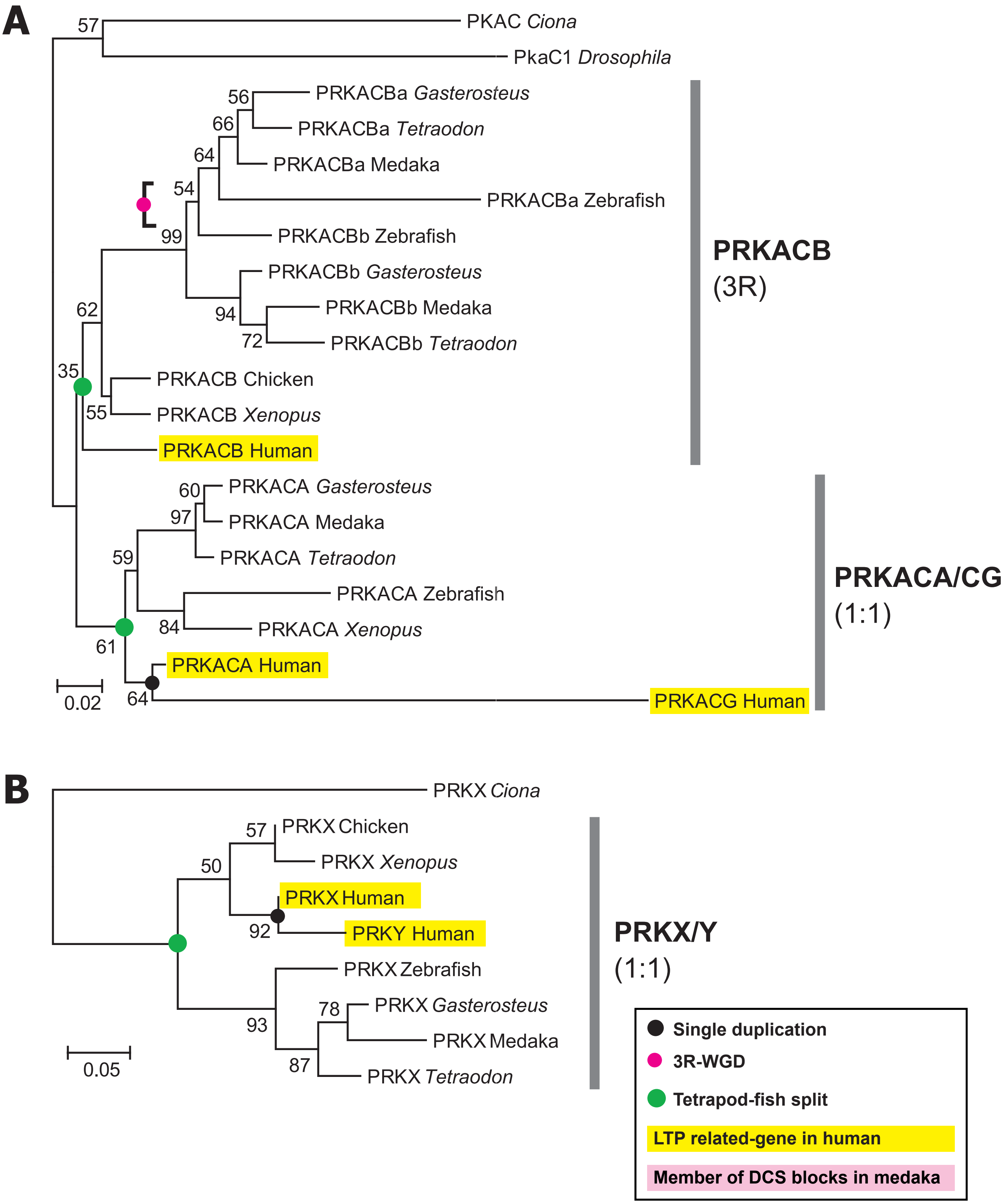

Fig. S7. A molecular phylogeny of PKA (protein kinase), inferred from maximum-likelihood analysis (panel A: 295 amino acid sites were used with JTT+Γ; panel B: 213 amino acid sites were used with JTT+Γ). Numbers indicate approximate bootstrap values from 1,000 LR-ELW (the Expected-Likelihood Weights applied to Local Rearrangements of tree topology) tests that support for the nodes.

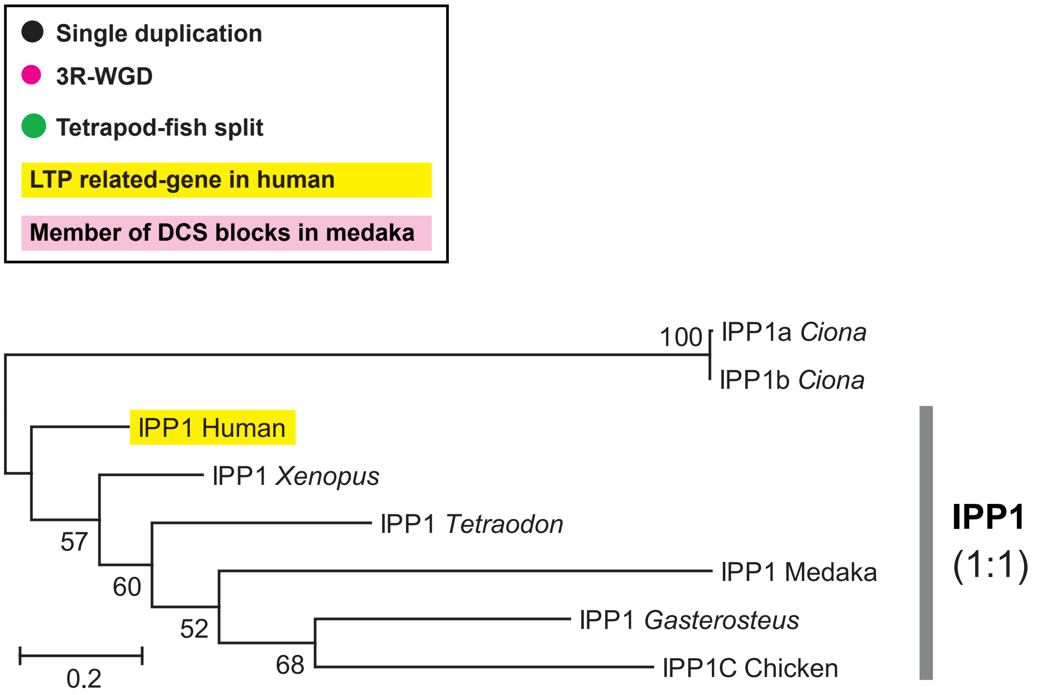

Fig. S8. A molecular phylogeny of IPP1 (protein phosphatase 1, regulatory [inhibitor] subunit 1A), inferred from maximum-likelihood analysis (307 nucleotide sites were used; TrN+I). Numbers indicate approximate bootstrap values from 1,000 LR-ELW (the Expected-Likelihood Weights applied to Local Rearrangements of tree topology) tests that support for the nodes.

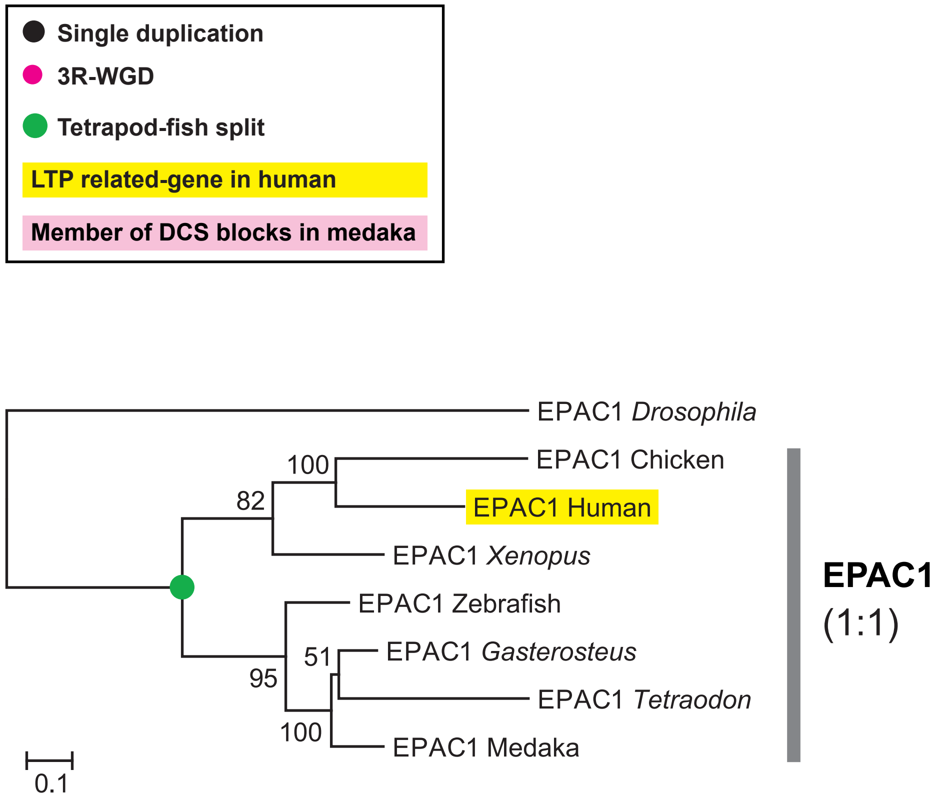

Fig. S9. A molecular phylogeny of EPAC1 (Rap guanine nucleotide exchange factor 3), inferred from maximum-likelihood analysis (701 amino acid sites were used; JTT+Γ). Numbers indicate approximate bootstrap values from 1,000 LR-ELW (the Expected-Likelihood Weights applied to Local Rearrangements of tree topology) tests that support for the nodes.

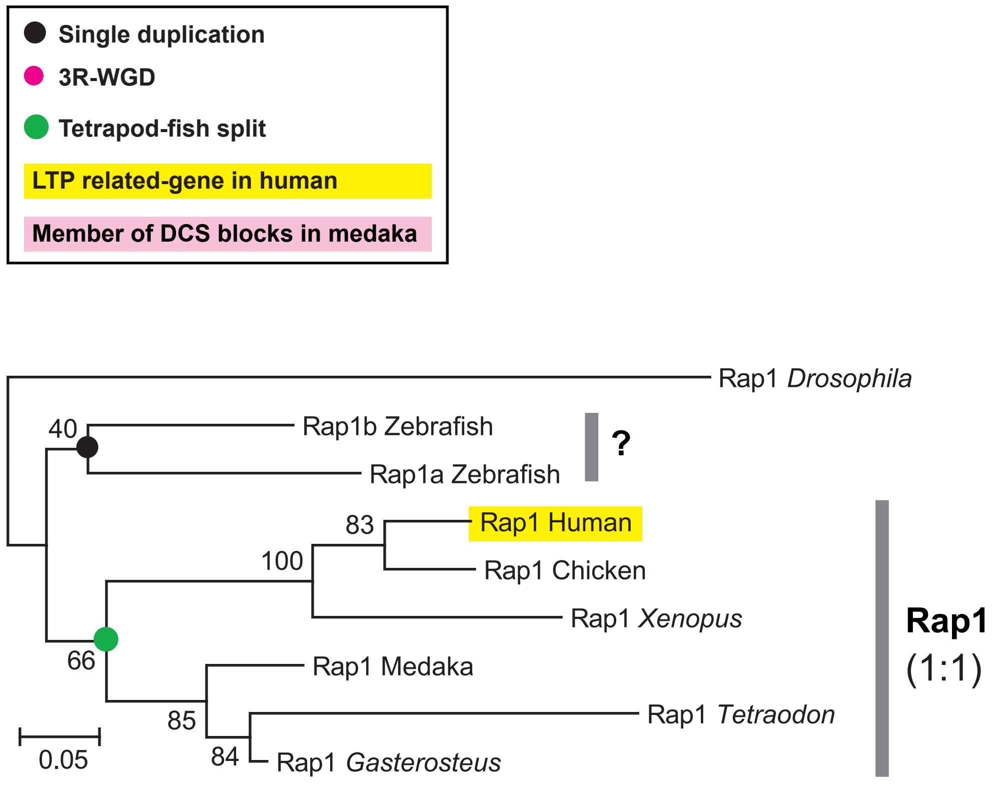

Fig. S10. A molecular phylogeny of Rap1 (or RAP1A, member of RAS oncogene family), inferred from maximum-likelihood analysis (548 nucleotide sites were used; GTR+I+Γ). Numbers indicate approximate bootstrap values from 1,000 LR-ELW (the Expected-Likelihood Weights applied to Local Rearrangements of tree topology) tests that support for the nodes.

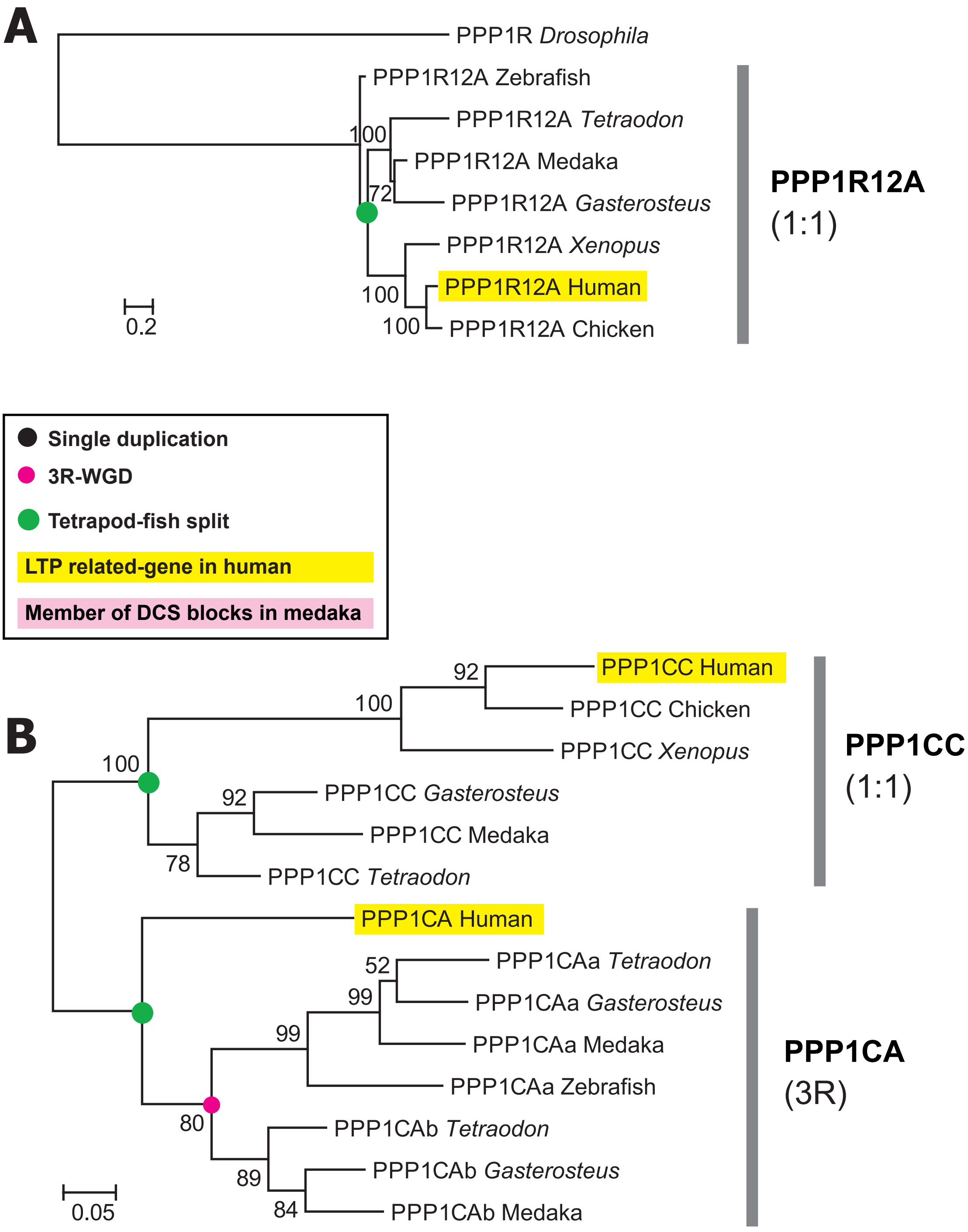

Fig. S11. A molecular phylogeny of PP1 (protein phosphatase 1), inferred from maximum-likelihood analysis (panel **A**: 807 amino acid sites were used with JTT+Γ; panel **B**: 909 nucleotide sites were used with TrN+I+Γ). Numbers indicate approximate bootstrap values from 1,000 LR-ELW (the Expected-Likelihood Weights applied to Local Rearrangements of tree topology) tests that support for the nodes.

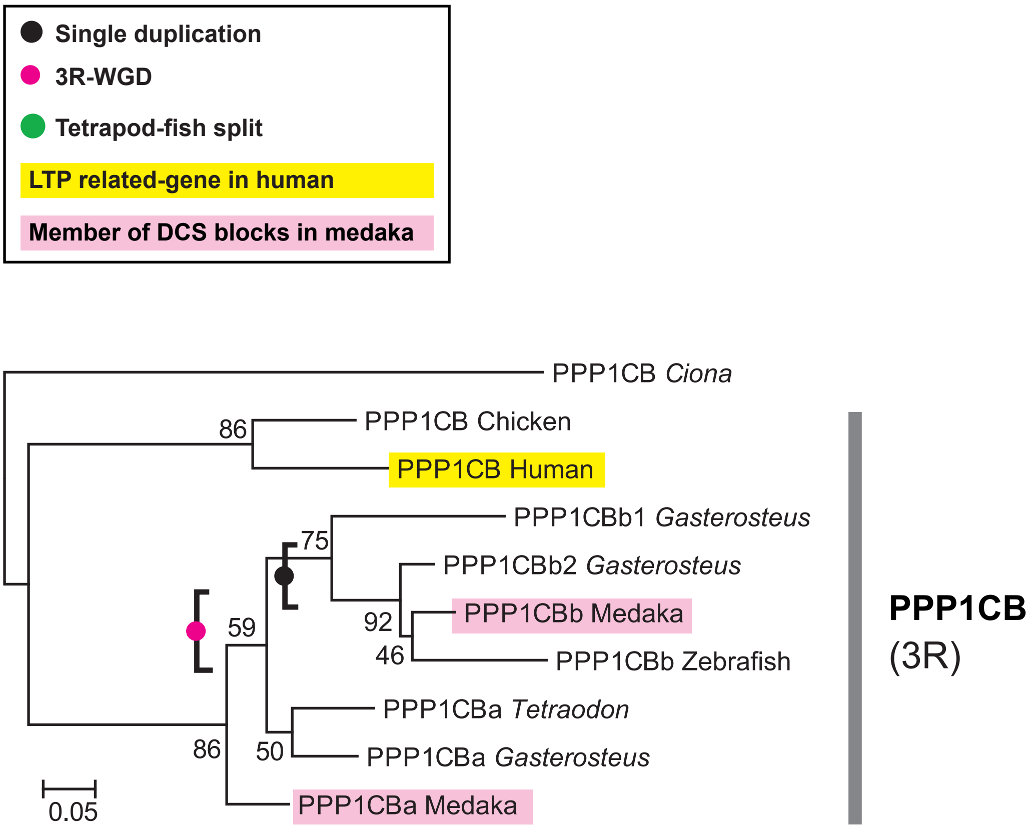

Fig. S12. A molecular phylogeny of PP1 (protein phosphatase 1), inferred from maximum-likelihood analysis (699 nucleotide sites were used; TrN+I+Γ). Numbers indicate approximate bootstrap values from 1,000 LR-ELW (the Expected-Likelihood Weights applied to Local Rearrangements of tree topology) tests that support for the nodes.

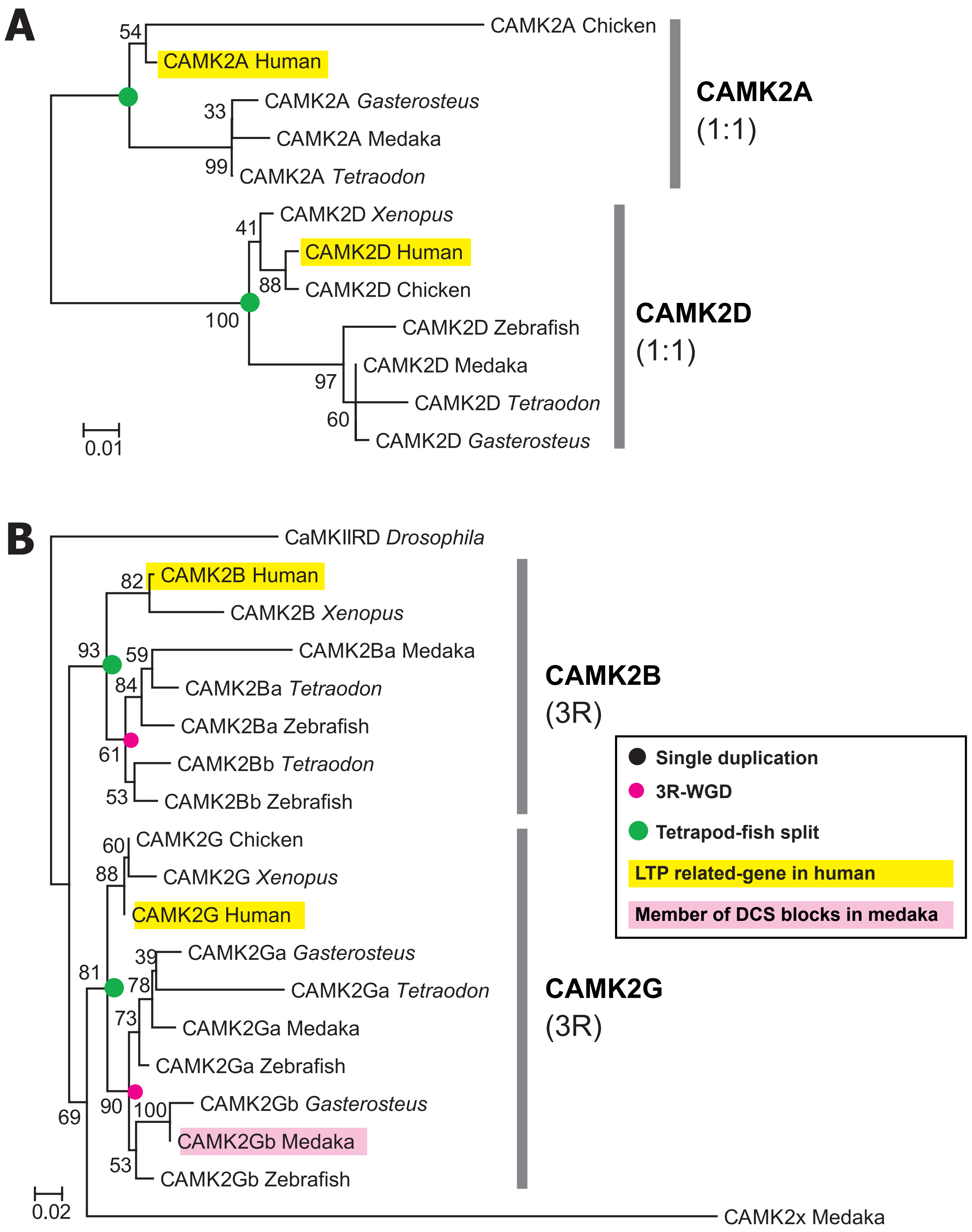

Fig. S13. A molecular phylogeny of CAMK2 (calcium/calmodulin-dependent protein kinase 2), inferred from maximum-likelihood analysis (panel **A**: 289 amino acid sites were used with JTT+Γ; panel **B**: 317 amino acid sites were used with JTT+Γ). Numbers indicate approximate bootstrap values from 1,000 LR-ELW (the Expected-Likelihood Weights applied to Local Rearrangements of tree topology) tests that support for the nodes.

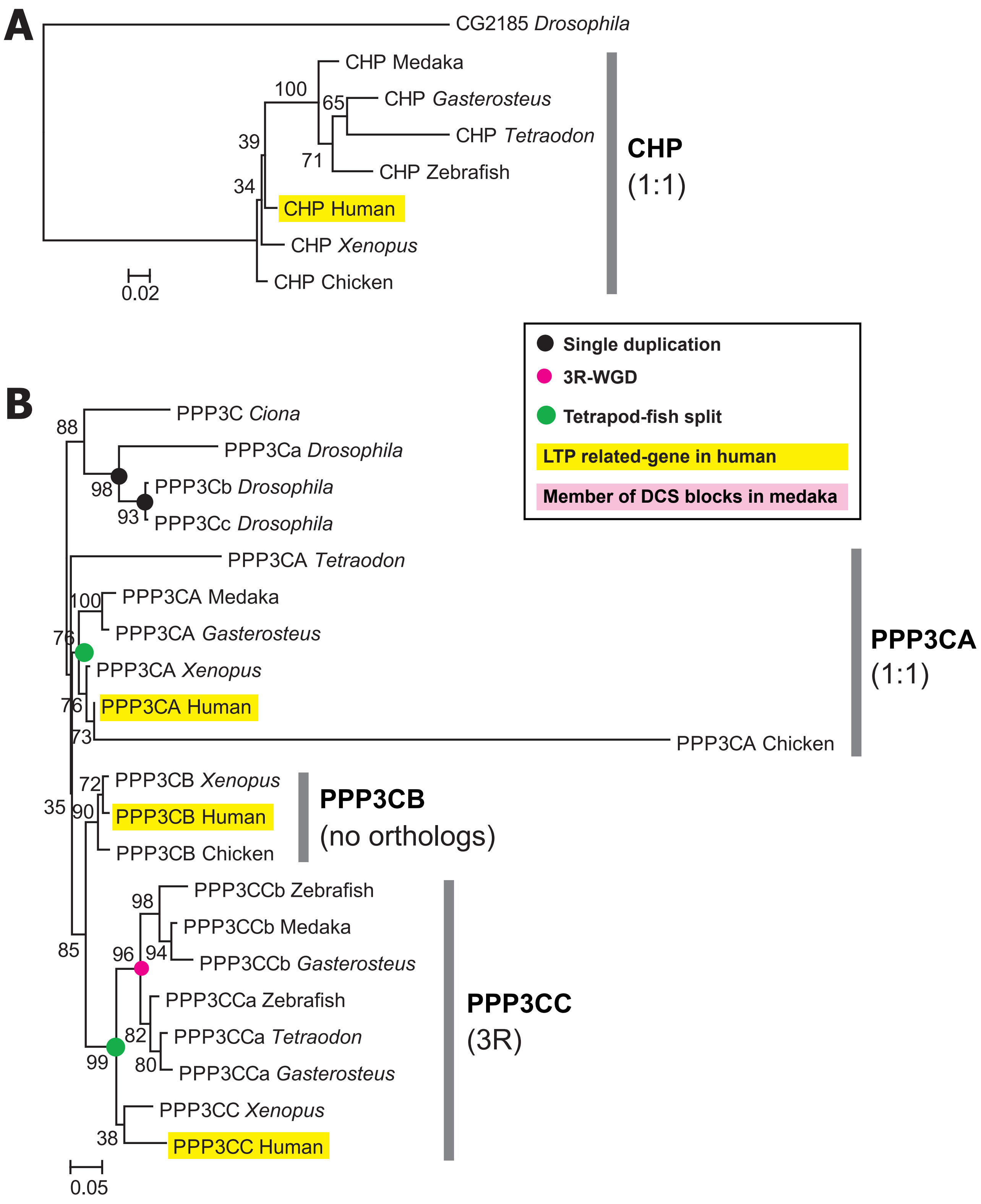

Fig. S14. A molecular phylogeny of CaN (calcium binding protein P22 and protein phosphatase 3), inferred from maximum-likelihood analysis (panel **A**: 186 amino acid sites were used with JTT+Γ; panel **B**: 352 amino acid sites were used with JTT+Γ). Numbers indicate approximate bootstrap values from 1,000 LR-ELW (the Expected-Likelihood Weights applied to Local Rearrangements of tree topology) tests that support for the nodes.

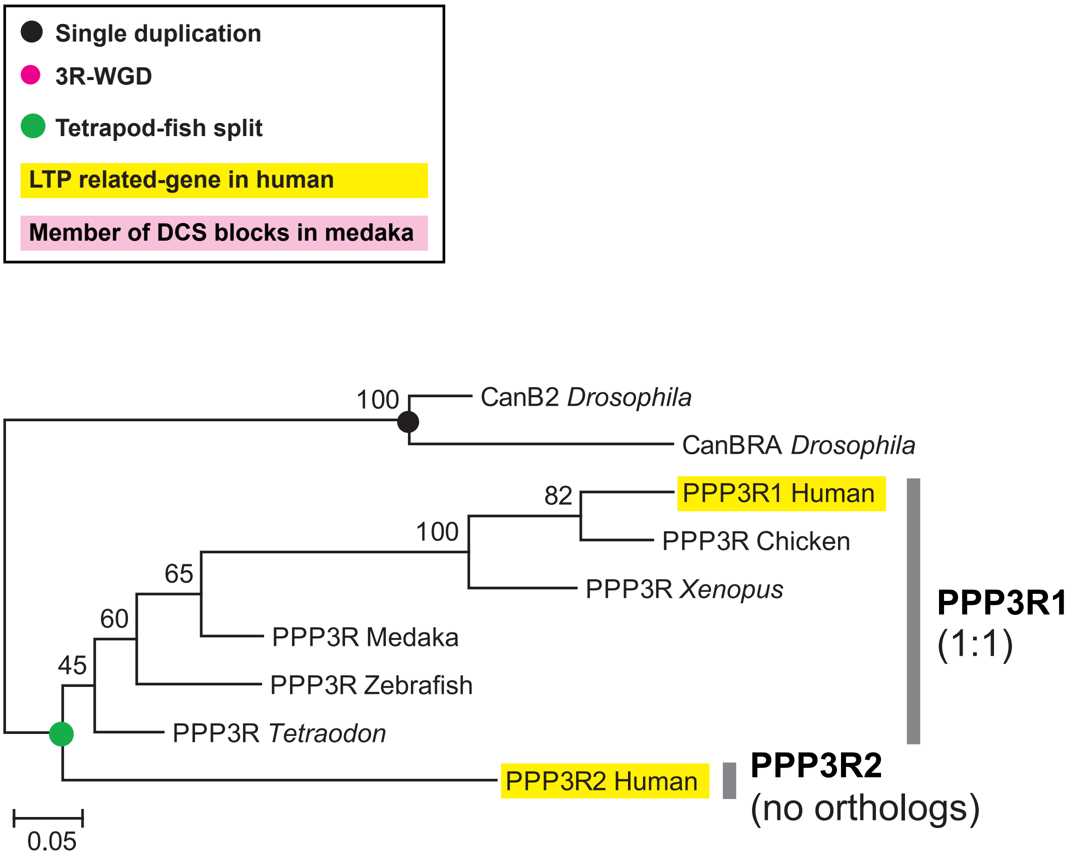

Fig. S15. A molecular phylogeny of CaN (calcium binding protein P22 and protein phosphatase 3), inferred from maximum-likelihood analysis (494 nucleotide sites were used; TrN+I+Γ). Numbers indicate approximate bootstrap values from 1,000 LR-ELW (the Expected-Likelihood Weights applied to Local Rearrangements of tree topology) tests that support for the nodes.

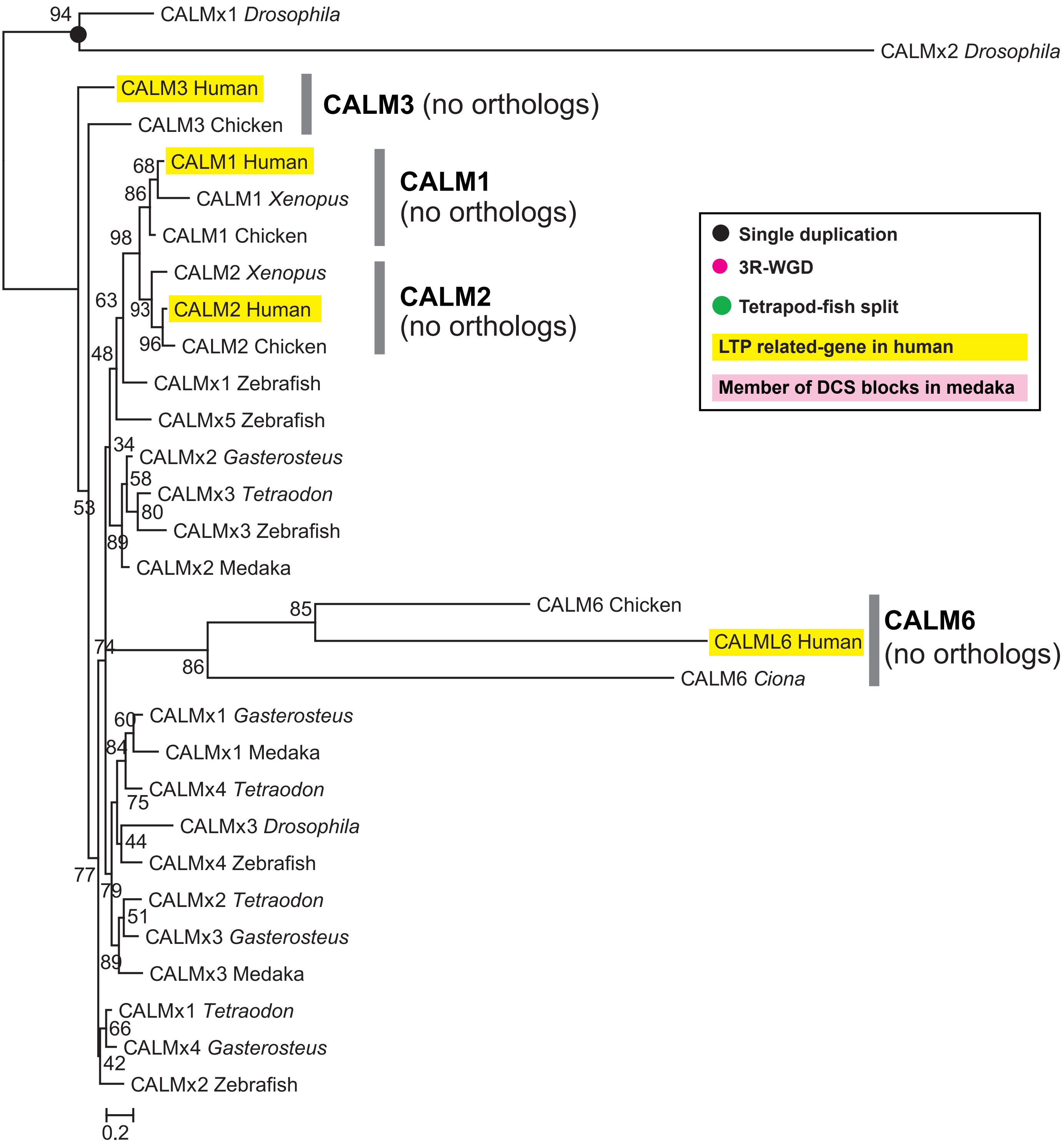

Fig. S16. A molecular phylogeny of CAM (calmodulin), inferred from maximum-likelihood analysis (408 nucleotide sites were used; TrN+Γ). Numbers indicate approximate bootstrap values from 1,000 LR-ELW (the Expected-Likelihood Weights applied to Local Rearrangements of tree topology) tests that support for the nodes.

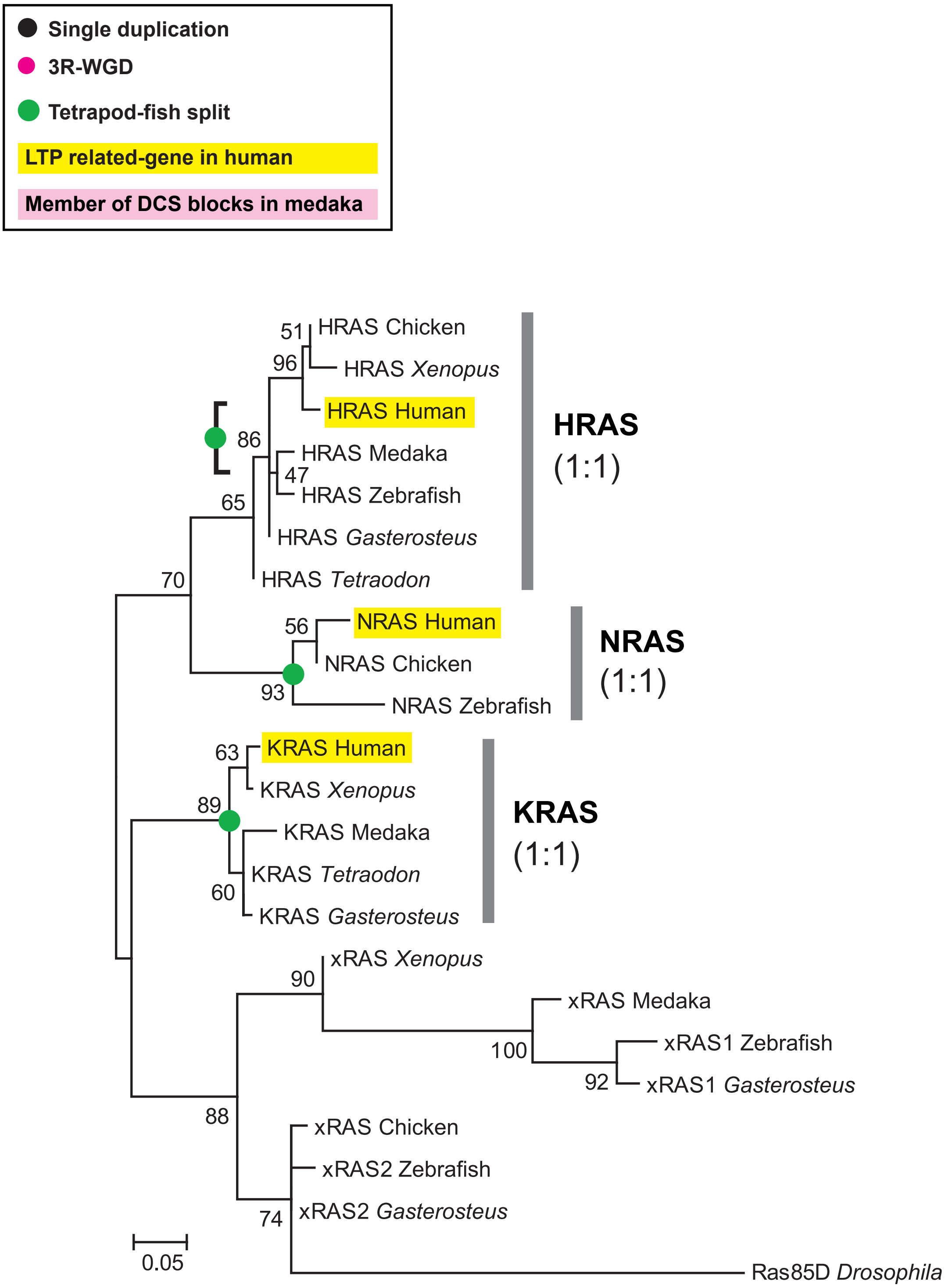

Fig. S17. A molecular phylogeny of Ras, inferred from maximum-likelihood analysis (182 amino acid sites were used; JTT+Γ). Numbers indicate approximate bootstrap values from 1,000 LR-ELW (the Expected-Likelihood Weights applied to Local Rearrangements of tree topology) tests that support for the nodes.

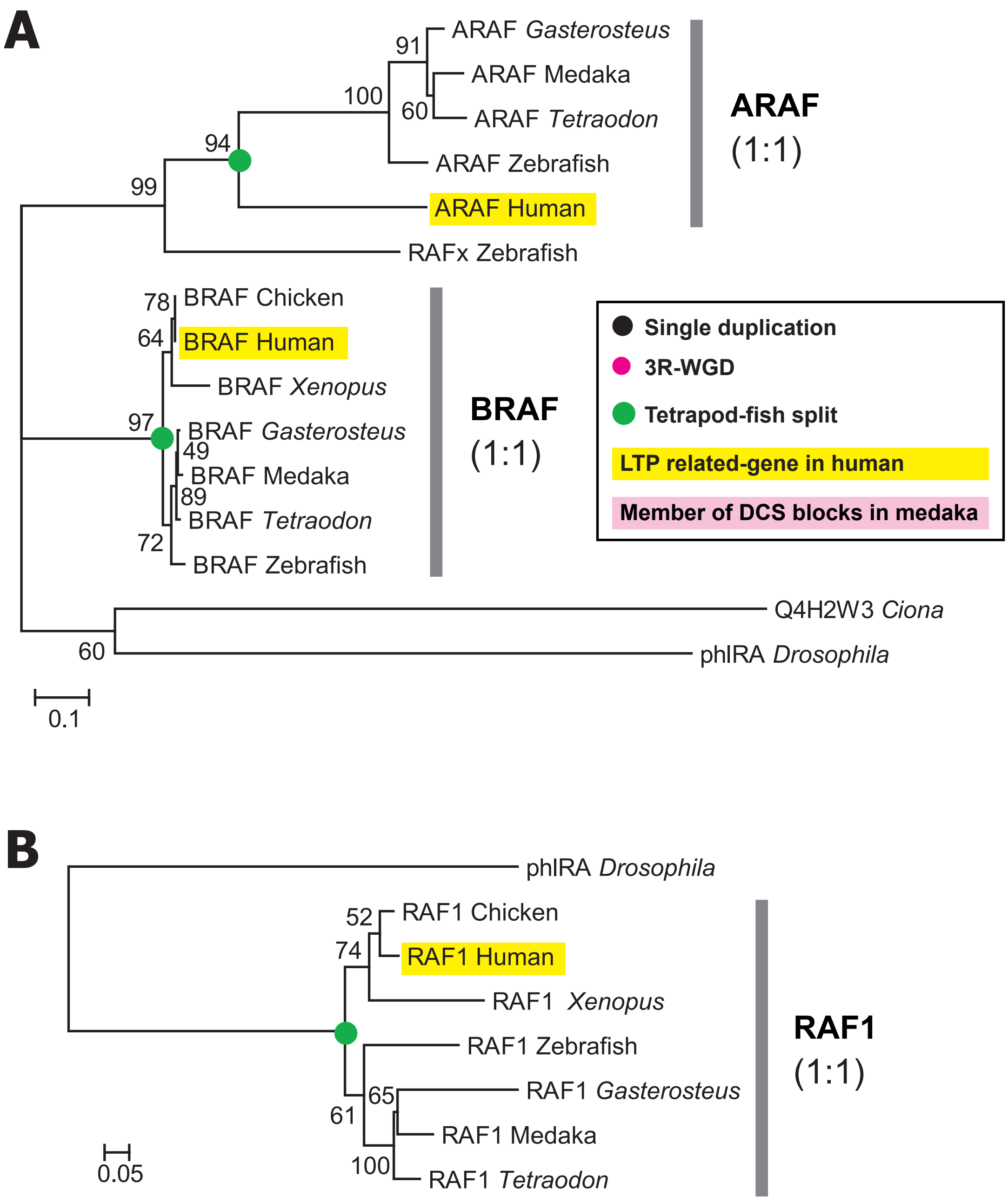

Fig. S18. A molecular phylogeny of Raf, inferred from maximum-likelihood analysis (panel **A**: 410 amino acid sites were used with JTT+Γ; panel **B**: 584 amino acid sites were used with JTT+Γ). Numbers indicate approximate bootstrap values from 1,000 LR-ELW (the Expected-Likelihood Weights applied to Local Rearrangements of tree topology) tests that support for the nodes.

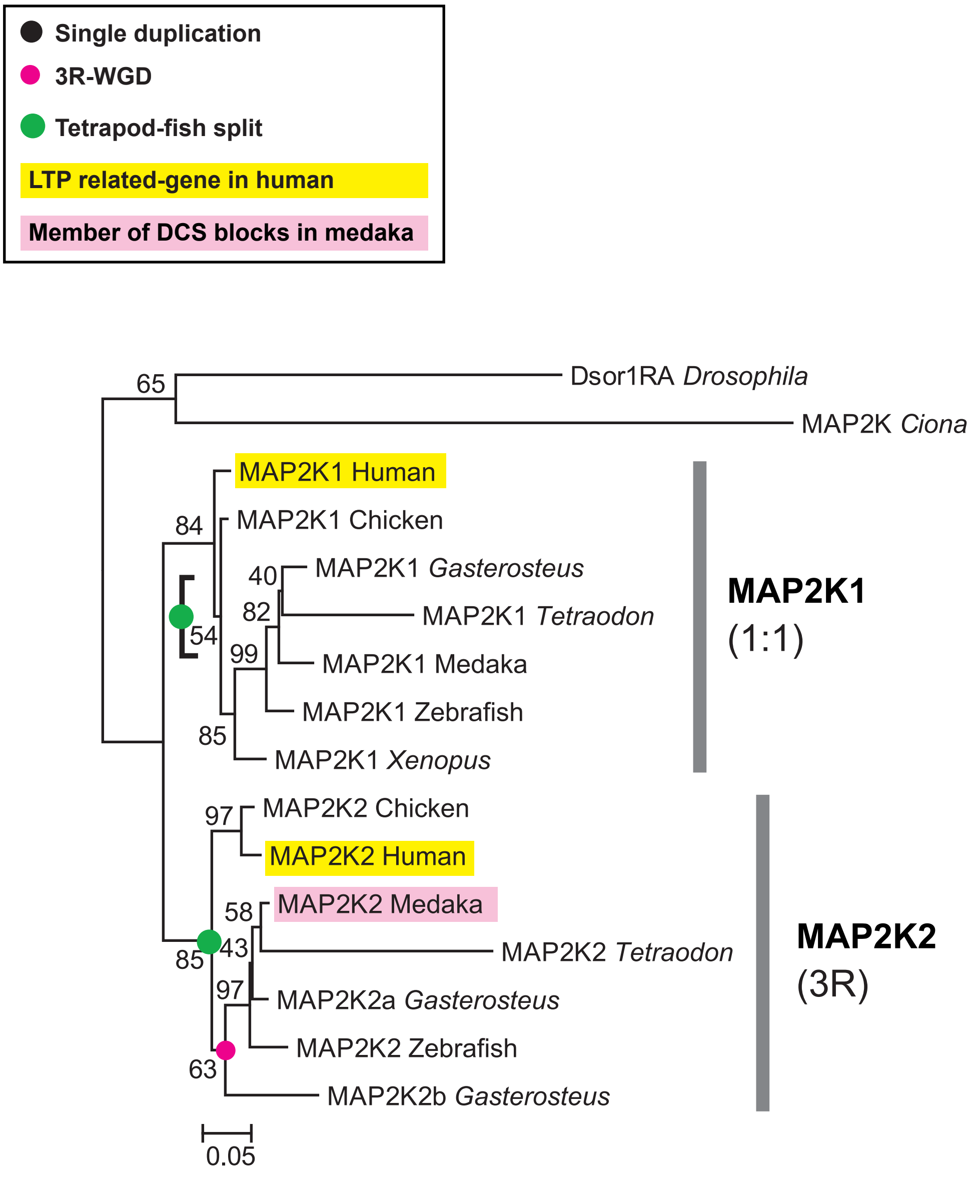

Fig. S19. A molecular phylogeny of MEK1/2 (mitogen-activated protein kinase kinase), inferred from maximum-likelihood analysis (339 amino acid sites were used; JTT+Γ). Numbers indicate approximate bootstrap values from 1,000 LR-ELW (the Expected-Likelihood Weights applied to Local Rearrangements of tree topology) tests that support for the nodes.

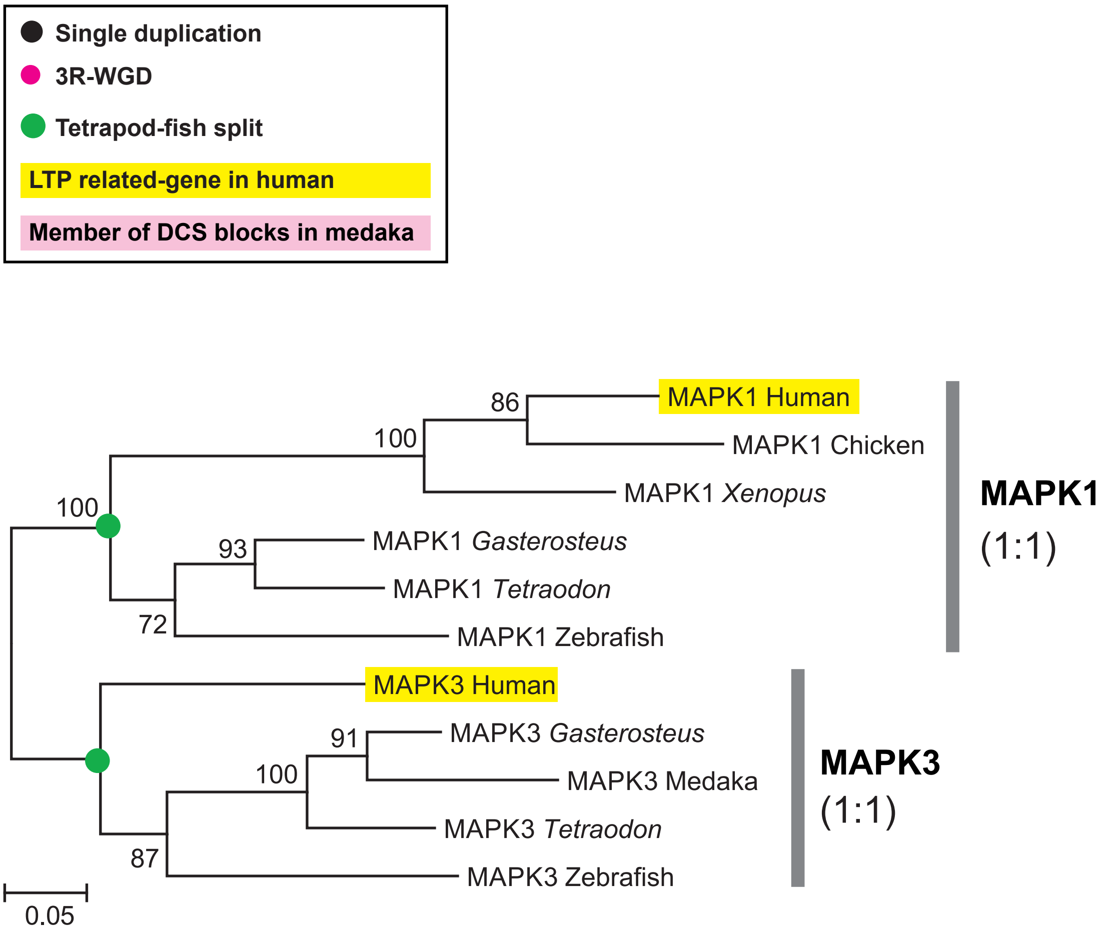

Fig. S20. A molecular phylogeny of ERK1/2 (mitogen-activated protein kinase), inferred from maximum-likelihood analysis (961 nucleotide sites were used; TrN+I+Γ). Numbers indicate approximate bootstrap values from 1,000 LR-ELW (the Expected-Likelihood Weights applied to Local Rearrangements of tree topology) tests that support for the nodes.

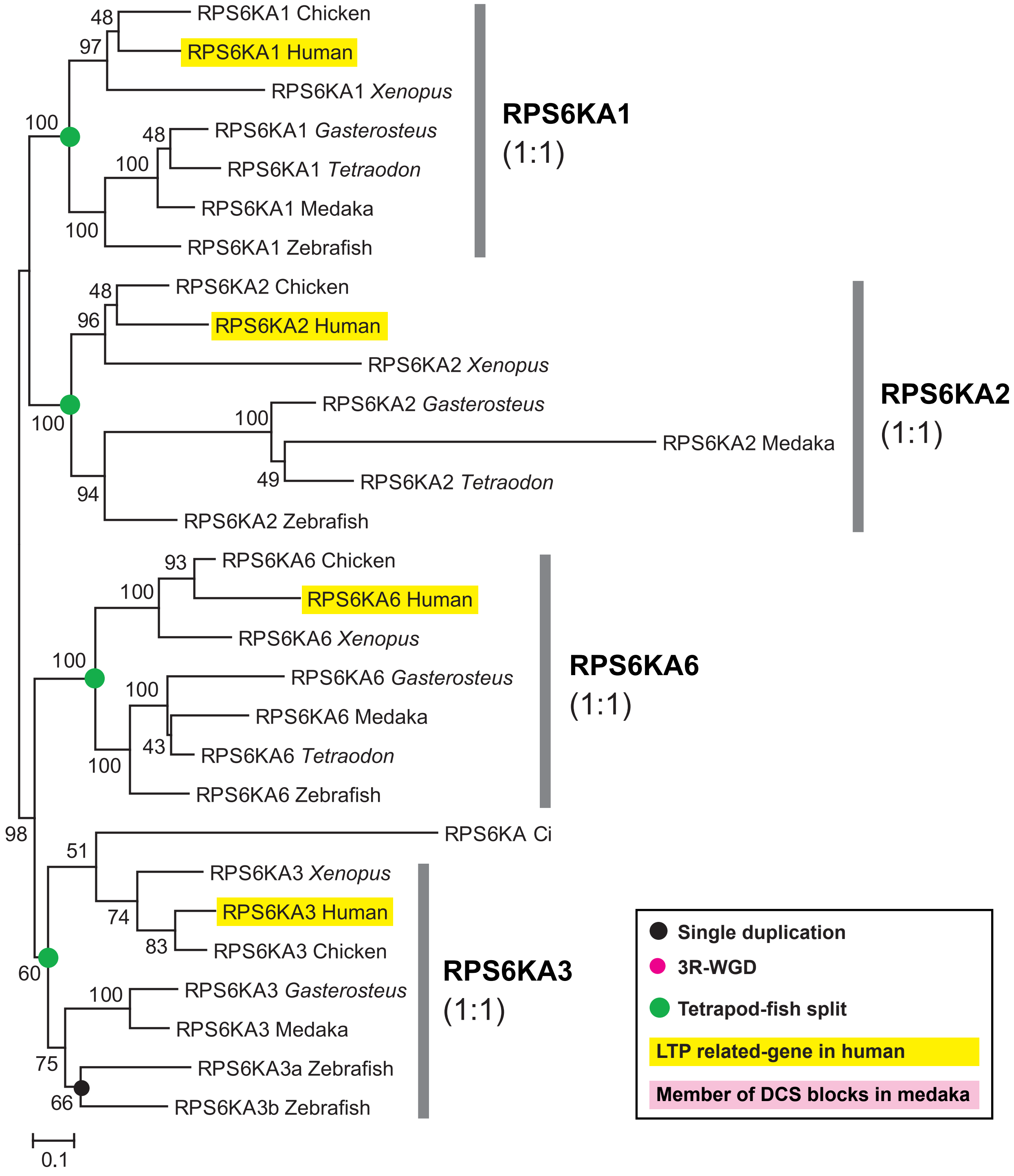

Fig. S21. A molecular phylogeny of Rsk (or RPS6KA, ribosomal protein S6 kinase), inferred from maximum-likelihood analysis (1087 nucleotide sites were used; GTR+Γ). Numbers indicate approximate bootstrap values from 1,000 LR-ELW (the Expected-Likelihood Weights applied to Local Rearrangements of tree topology) tests that support for the nodes.

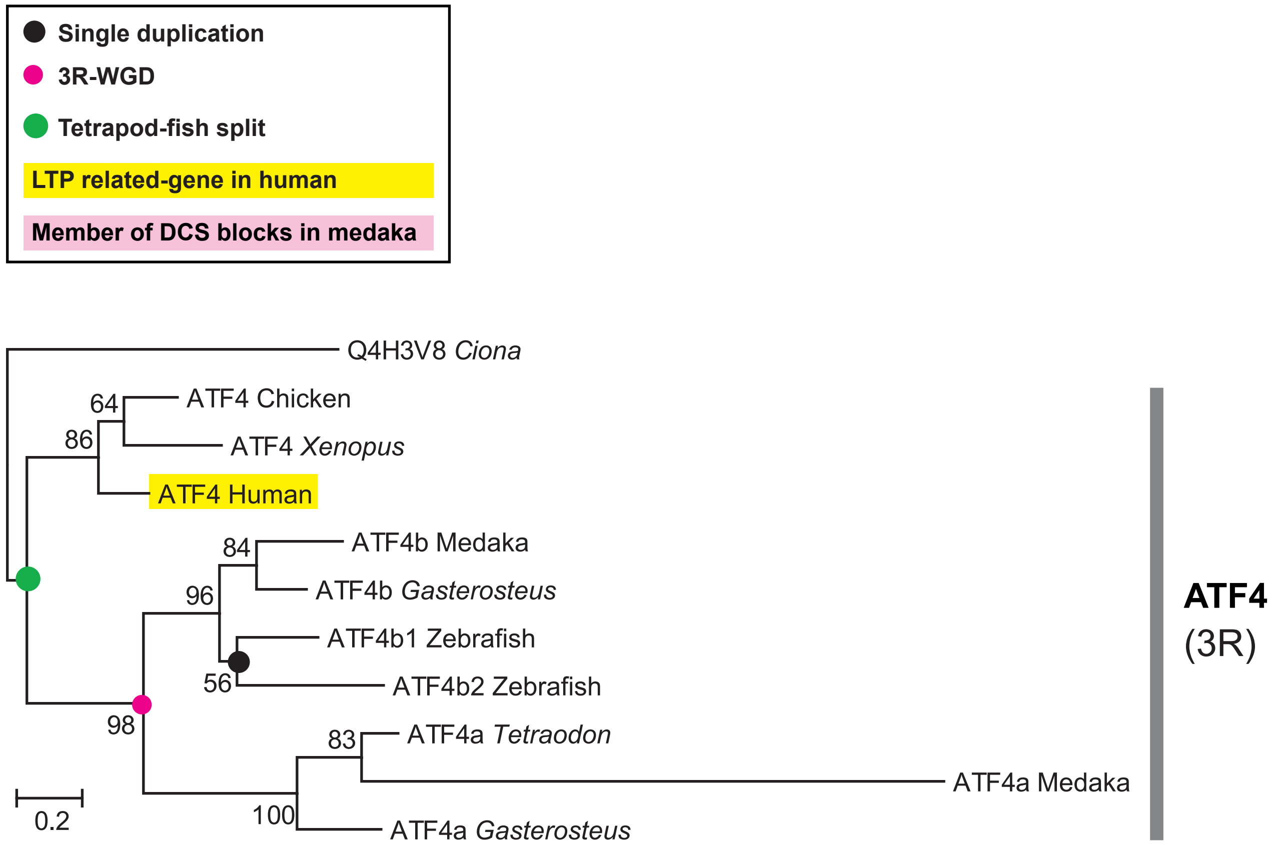

Fig. S22. A molecular phylogeny of CREB (or ATF4, activating transcription factor 4), inferred from maximum-likelihood analysis (724 nucleotide sites were used; TrN+Γ). Numbers indicate approximate bootstrap values from 1,000 LR-ELW (the Expected-Likelihood Weights applied to Local Rearrangements of tree topology) tests that support for the nodes.

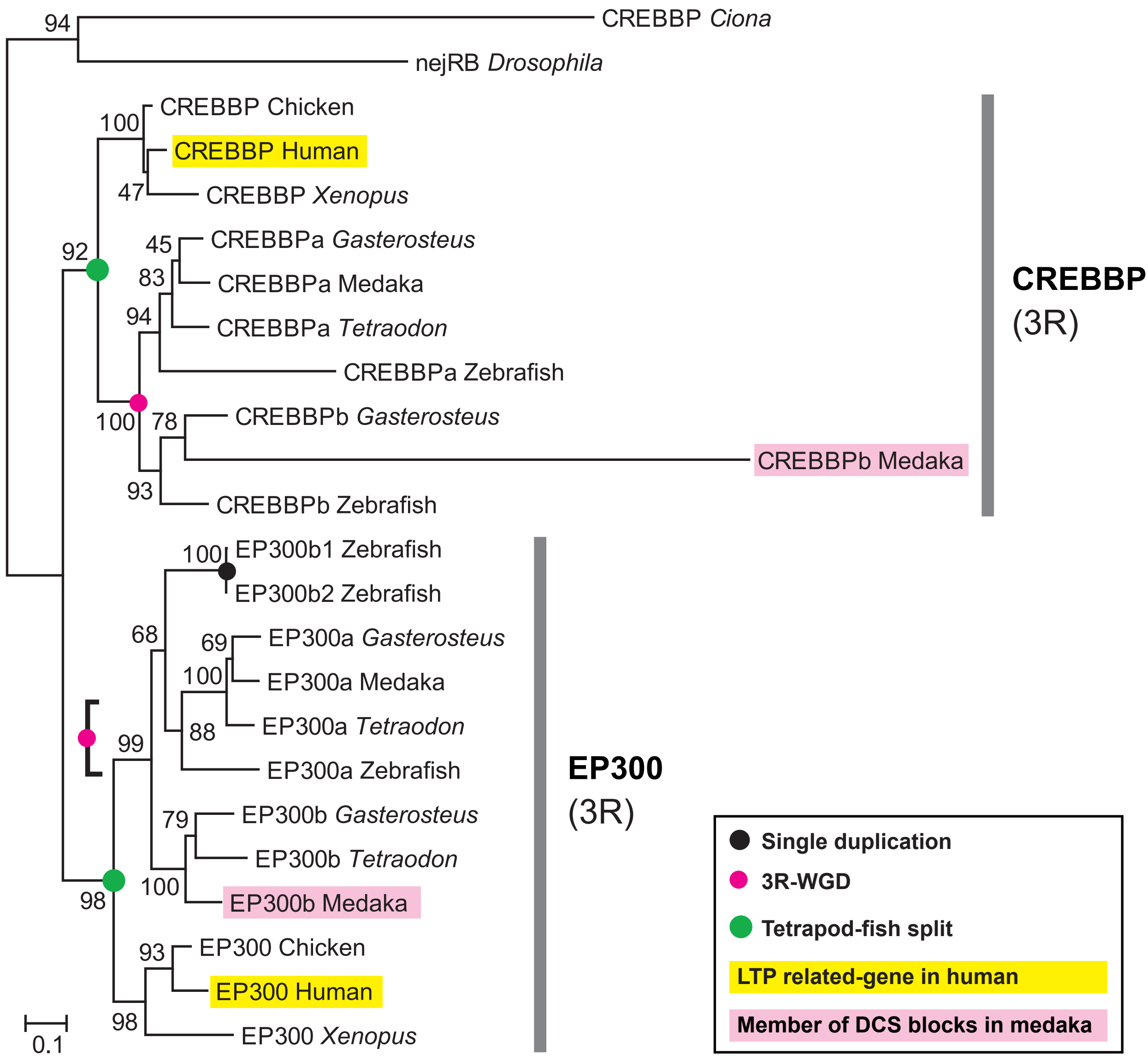

Fig. S23. A molecular phylogeny of CBP (or CREB binding protein), inferred from maximum-likelihood analysis (817 amino acid sites were used; JTT+Γ). Numbers indicate approximate bootstrap values from 1,000 LR-ELW (the Expected-Likelihood Weights applied to Local Rearrangements of tree topology) tests that support for the nodes.

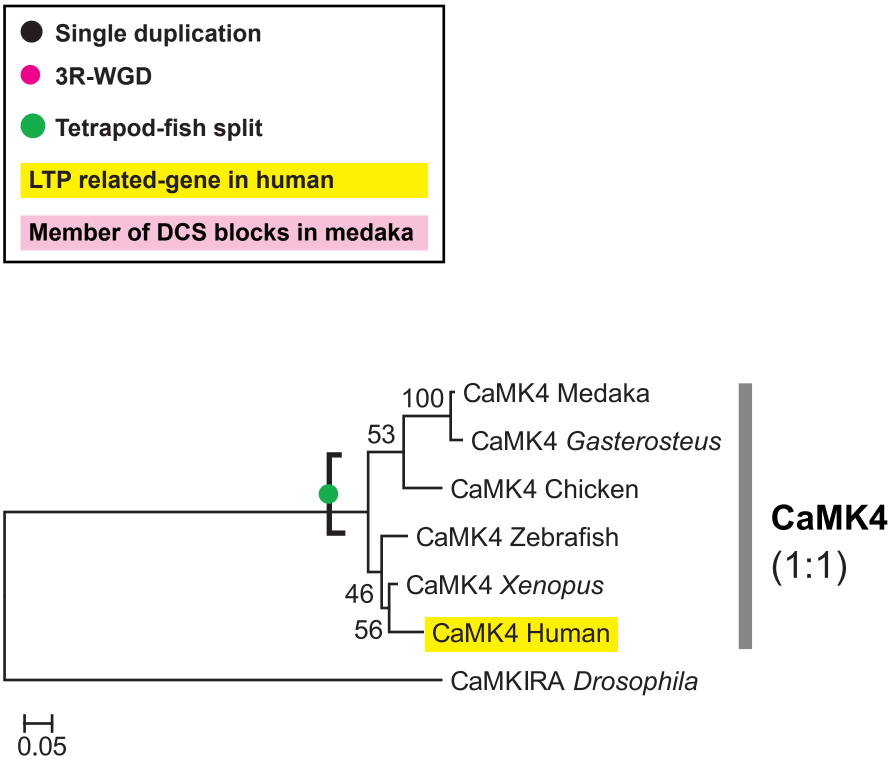

Fig. S24. A molecular phylogeny of CaMK4 (calcium/calmodulin-dependent protein kinase IV), inferred from maximum-likelihood analysis (829 nucleotide sites were used; HKY+I+Γ). Numbers indicate approximate bootstrap values from 1,000 LR-ELW (the Expected-Likelihood Weights applied to Local Rearrangements of tree topology) tests that support for the nodes.

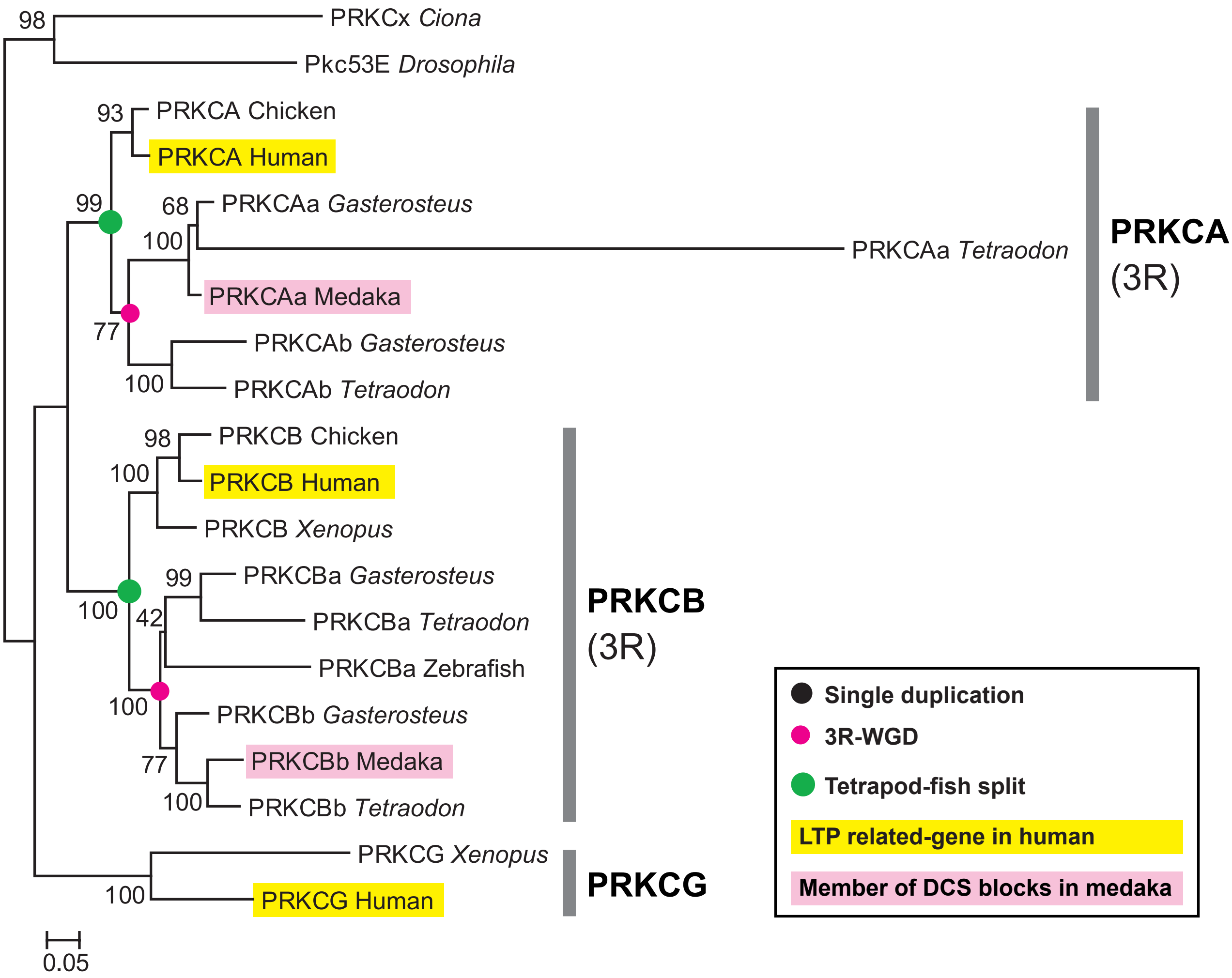

Fig. S25. A molecular phylogeny of PKC (protein kinase C), inferred from maximum-likelihood analysis (531 amino acid sites were used; JTT+Γ). Numbers indicate approximate bootstrap values from 1,000 LR-ELW (the Expected-Likelihood Weights applied to Local Rearrangements of tree topology) tests that support for the nodes.

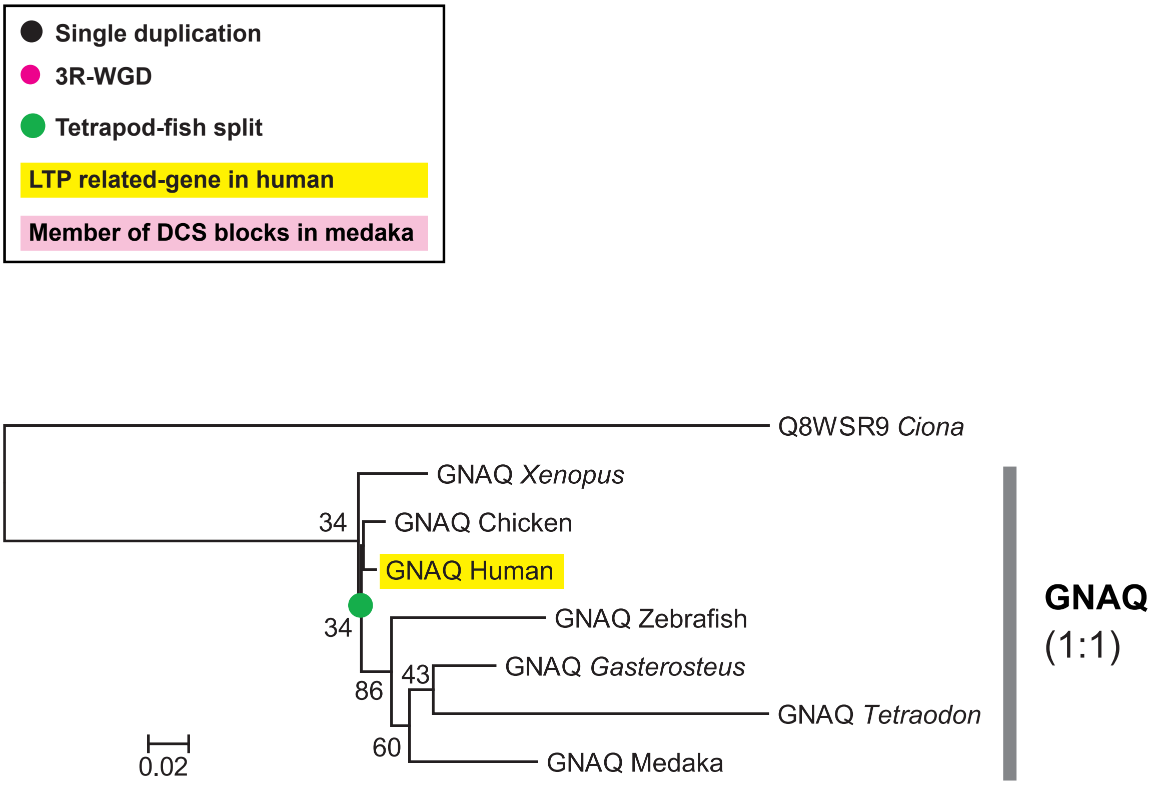

Fig. S26. A molecular phylogeny of Gq (or GNAQ, guanine nucleotide binding protein [G protein], q polypeptide), inferred from maximum-likelihood analysis (332 amino acid sites were used; JTT+Γ). Numbers indicate approximate bootstrap values from 1,000 LR-ELW (the Expected-Likelihood Weights applied to Local Rearrangements of tree topology) tests that support for the

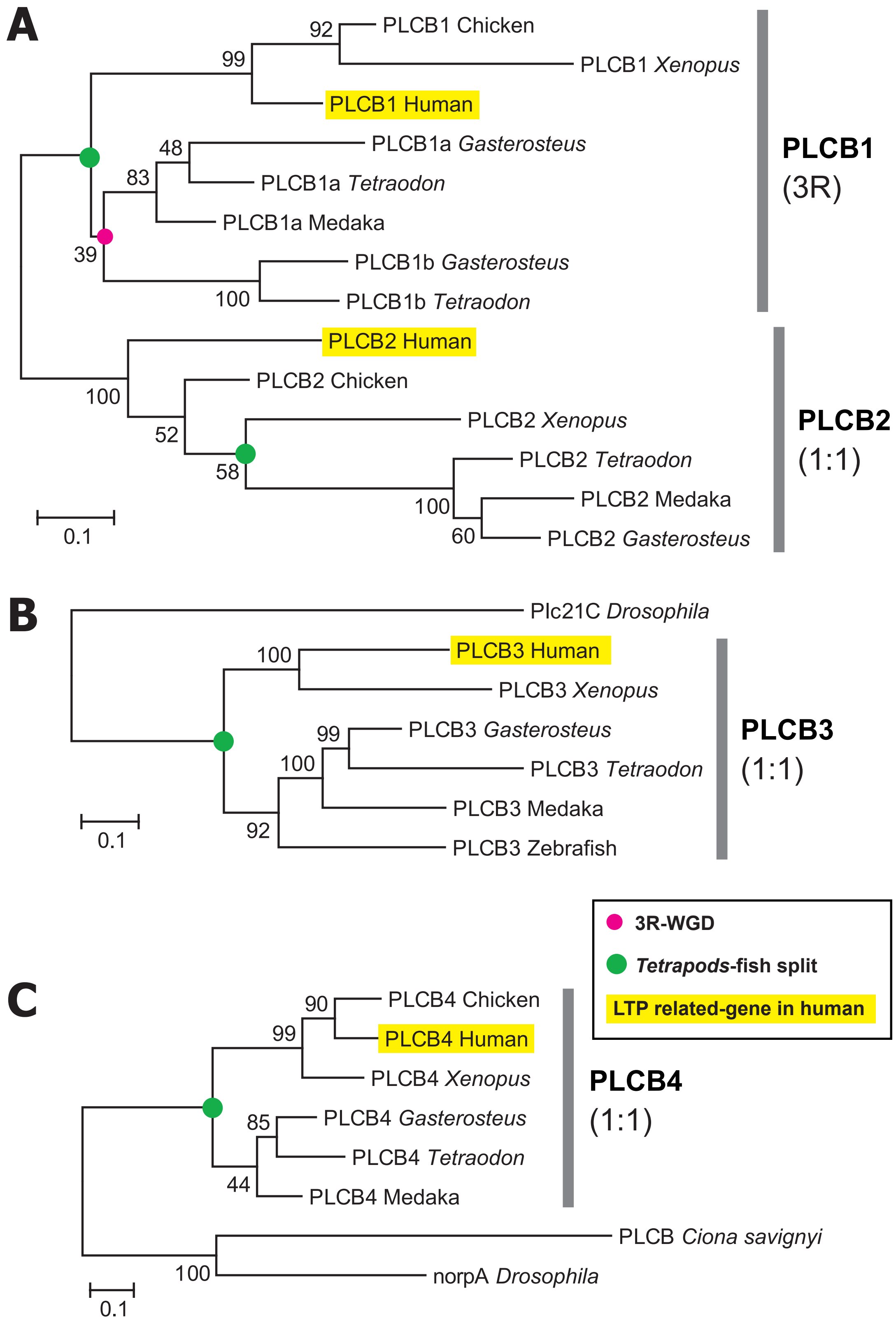

Fig. S27. A molecular phylogeny of PLCB (phospholipase C, beta), inferred from maximum-likelihood analysis (panel A: 394 nucleotide sites were used with HKY+I+Γ; panel B: 3116 nucleotide sites were used with GTR+I+Γ; panel C: 1066 nucleotide sites were used with GTR+Γ). Numbers indicate approximate bootstrap values from 1,000 LR-ELW (the Expected-Likelihood Weights applied to Local Rearrangements of tree topology) tests that support for the nodes. Conserved synteny around the PLCB1 loci among human and teleosts is shown in Fig. S64.

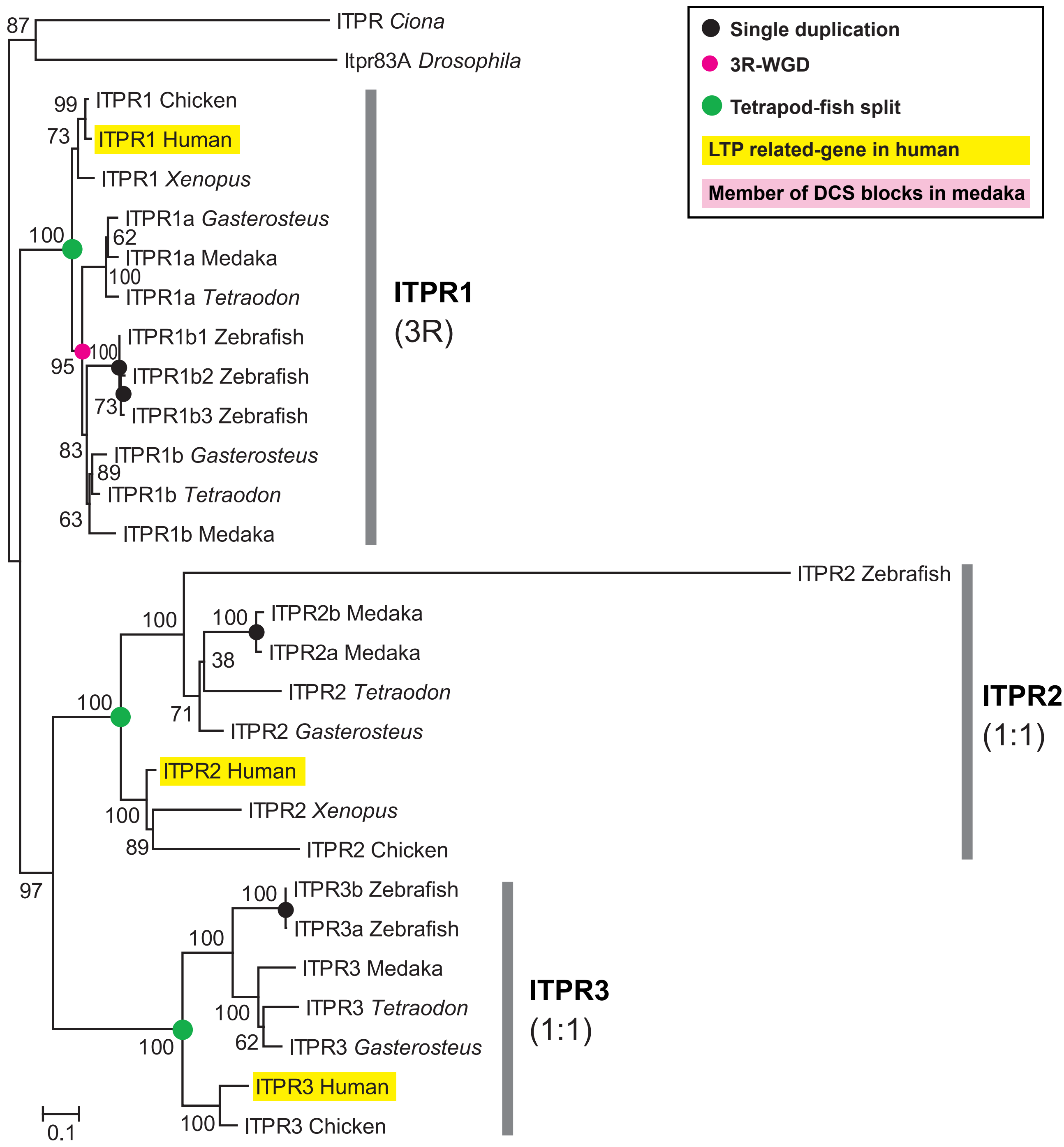

Fig. S28. A molecular phylogeny of IPR (or ITPR, inositol 1,4,5-triphosphate receptor), inferred from maximum-likelihood analysis (1022 amino acid sites were used; JTT+Γ). Numbers indicate approximate bootstrap values from 1,000 LR-ELW (the Expected-Likelihood Weights applied to Local Rearrangements of tree topology) tests that support for the nodes.

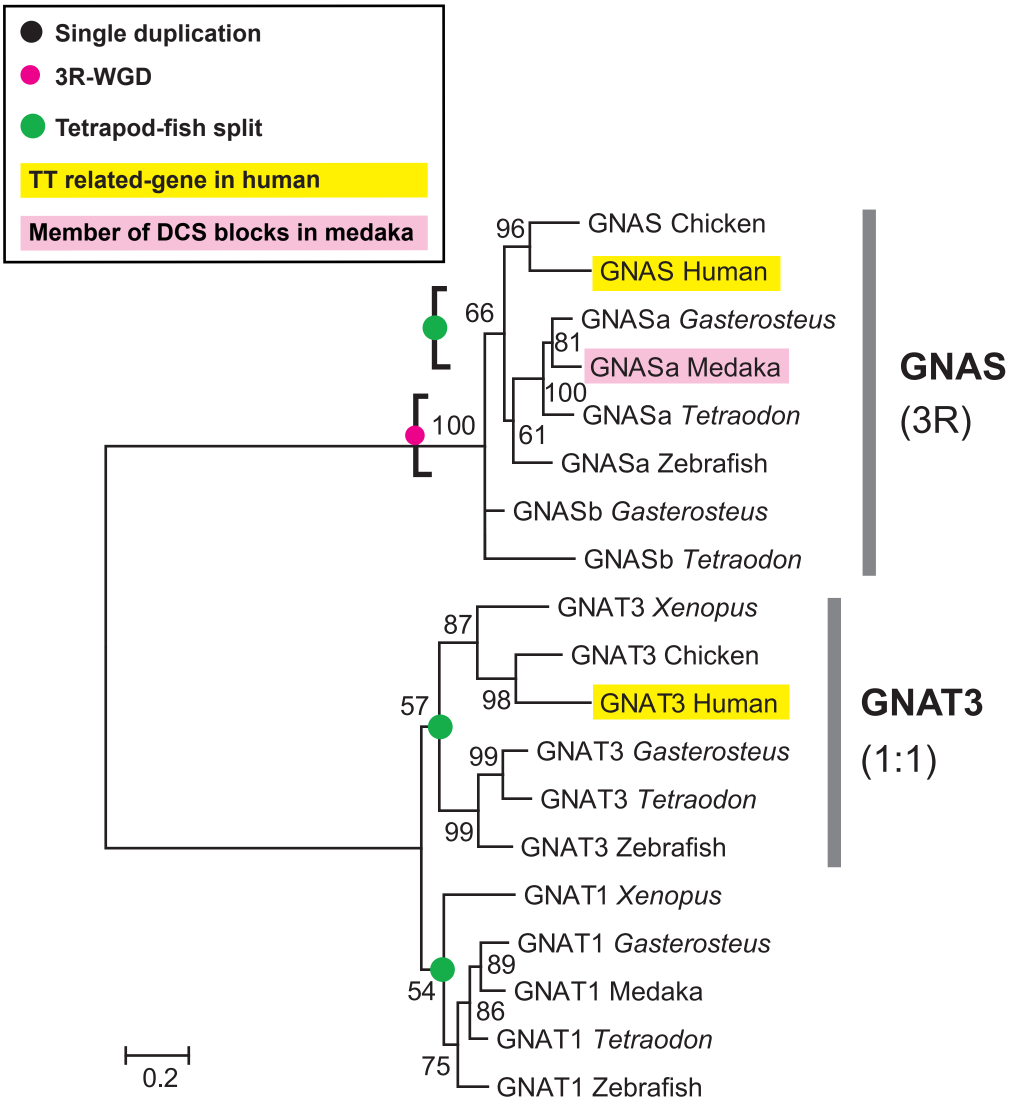

Fig. S29. A molecular phylogeny of Gα (guanine nucleotide binding protein, alpha transducing), inferred from maximum-likelihood analysis (744 amino acid sites were used; JTT+Γ). Numbers indicate approximate bootstrap values from 1,000 LR-ELW (the Expected-Likelihood Weights applied to Local Rearrangements of tree topology) tests that support for the nodes.

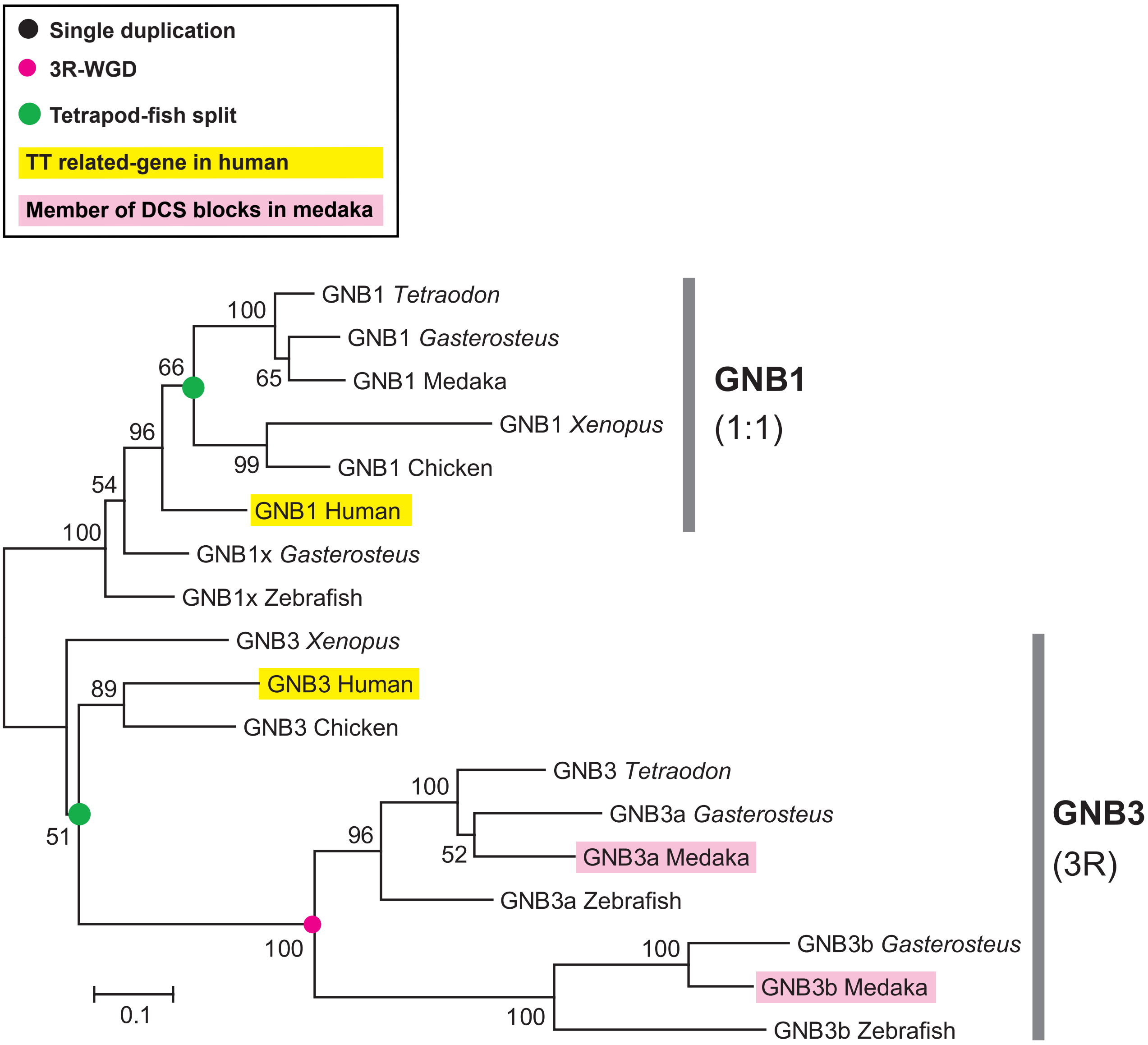

Fig. S30. A molecular phylogeny of GNB (guanine nucleotide binding protein, beta polypeptide), inferred from maximum-likelihood analysis (934 nucleotide sites were used; GTR+I+Γ). Numbers indicate approximate bootstrap values from 1,000 LR-ELW (the Expected-Likelihood Weights applied to Local Rearrangements of tree topology) tests that support for the nodes.

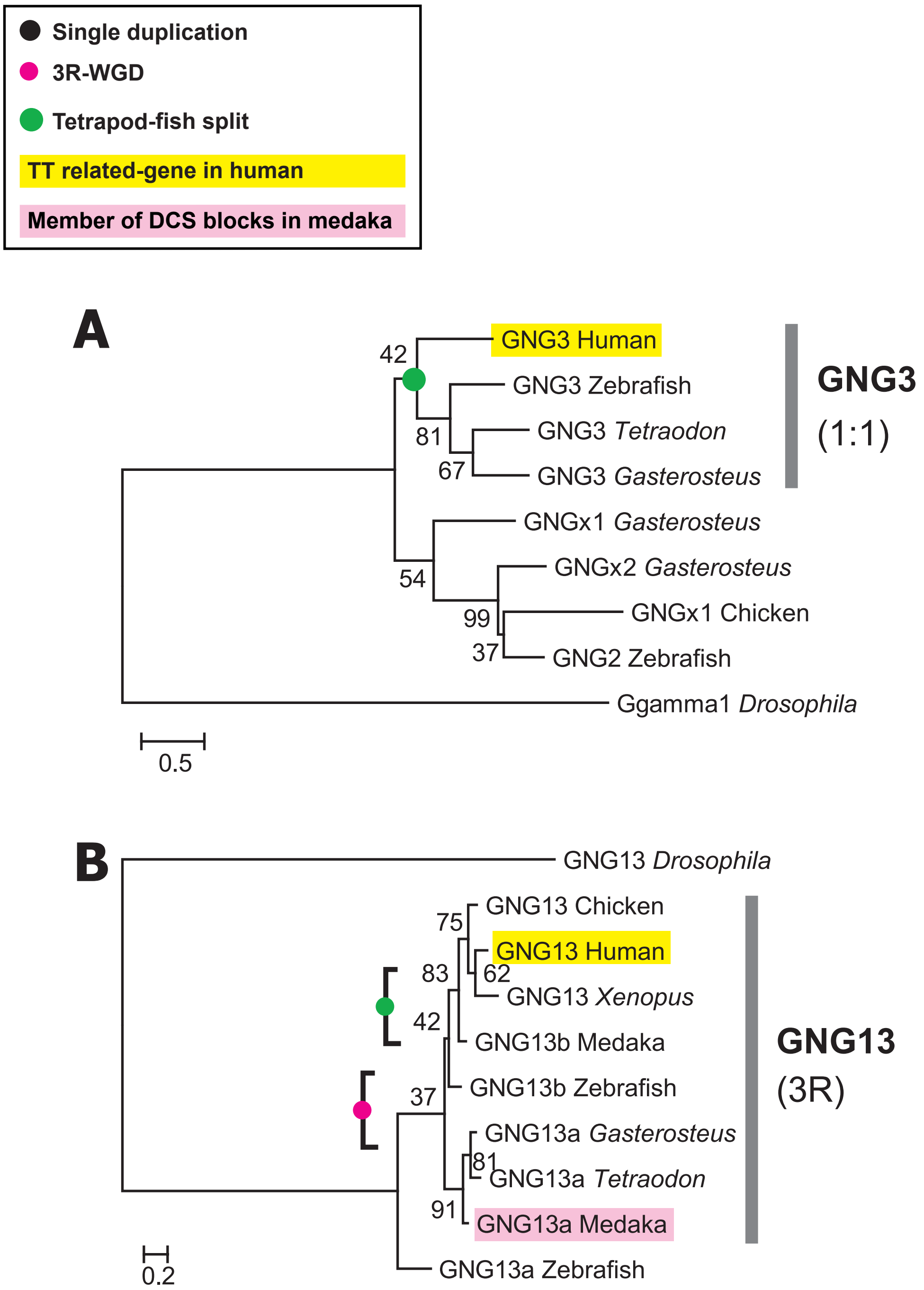

Fig. S31. A molecular phylogeny of GNG3 and 13 (guanine nucleotide binding protein, gamma polypeptide 3 and 13), inferred from maximum-likelihood analysis (panel **A**: 531 nucleotide sites were used with GTR+I+Γ; panel **B**: 204 nucleotide sites were used with TrN+Γ). Numbers indicate approximate bootstrap values from 1,000 LR-ELW (the Expected-Likelihood Weights applied to Local Rearrangements of tree topology) tests that support for the nodes.

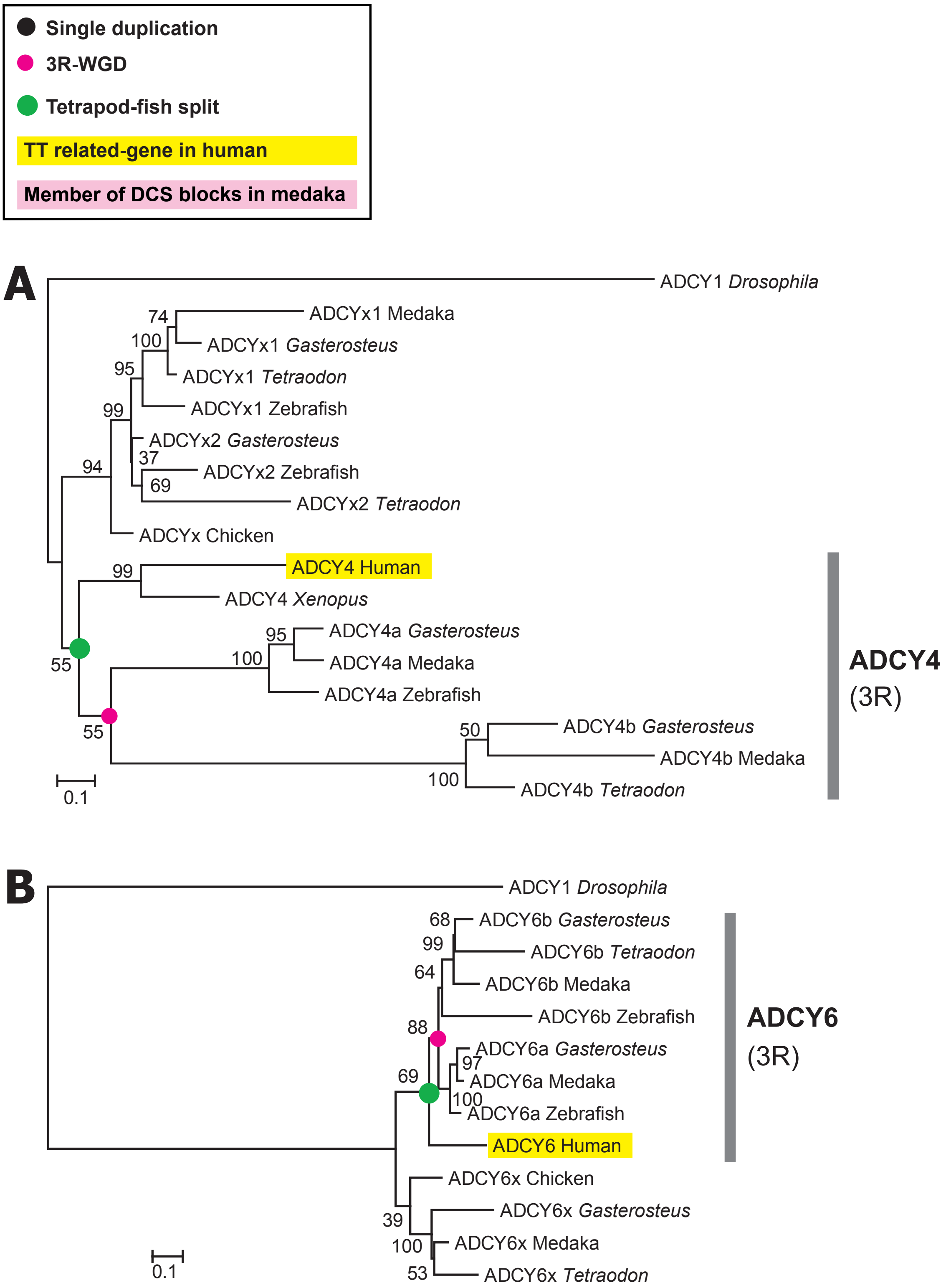

Fig. S32. A molecular phylogeny of AC (adenylate cyclase, EC:4.6.1.1), inferred from maximum-likelihood analysis (panel A: 571 amino acid sites were used with JTT+Γ; panel B: 680 amino acid sites were used with Blosum62+Γ). Numbers indicate approximate bootstrap values from 1,000 LR-ELW (the Expected-Likelihood Weights applied to Local Rearrangements of tree topology) tests that support for the nodes.

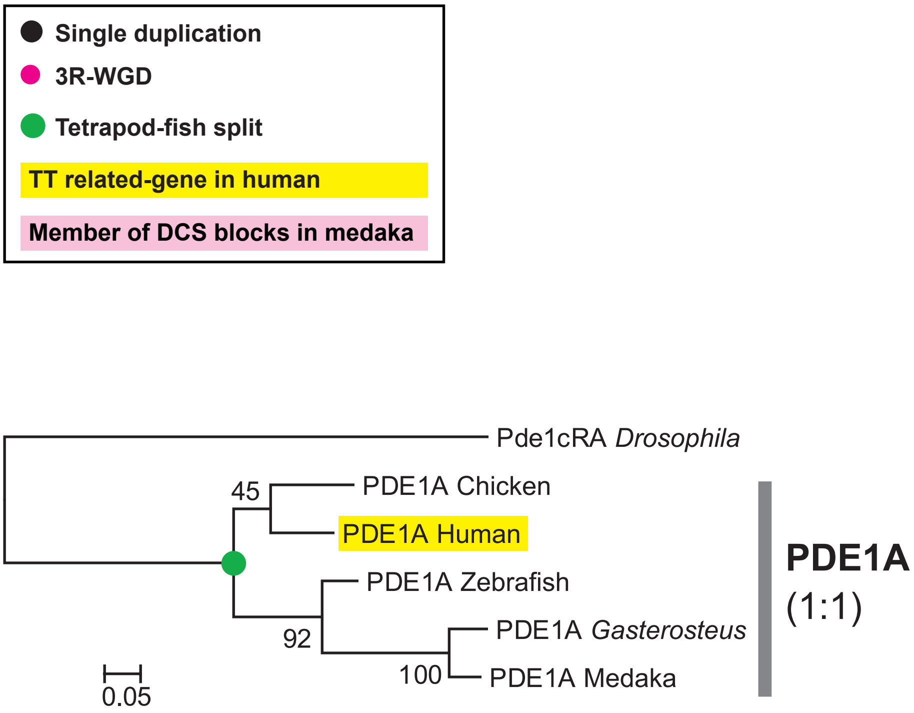

Fig. S33. A molecular phylogeny of PDE1A (phosphodiesterase 1A, calmodulin-dependent, EC:3.1.4.17), inferred from maximum-likelihood analysis (435 amino acid sites were used; JTT+Γ). Numbers indicate approximate bootstrap values from 1,000 LR-ELW (the Expected-Likelihood Weights applied to Local Rearrangements of tree topology) tests that support for the nodes.

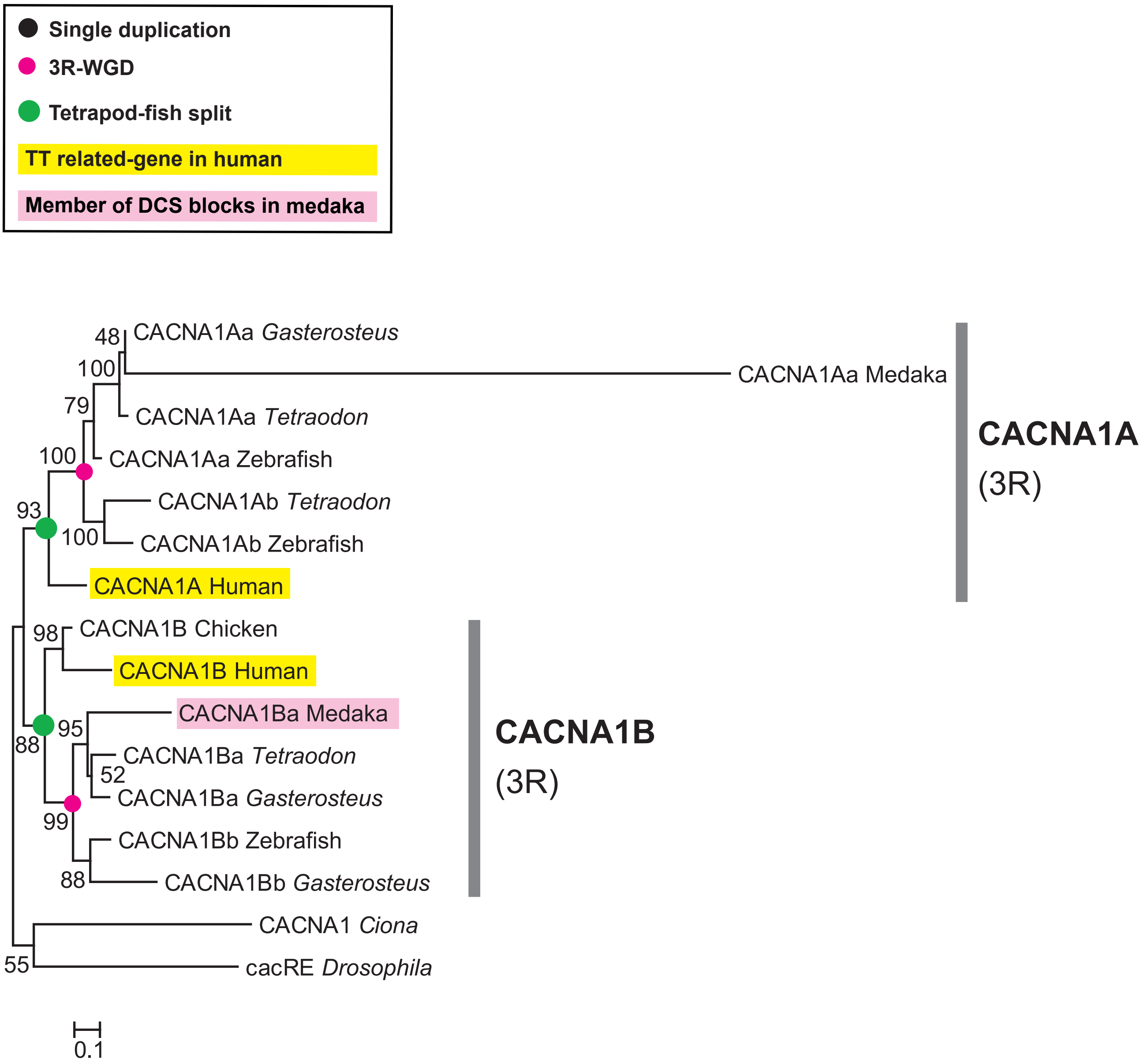

Fig. S34. A molecular phylogeny of CACN (calcium channel),, inferred from maximum-likelihood analysis (343 amino acid sites were used; JTT+Γ). Numbers indicate approximate bootstrap values from 1,000 LR-ELW (the Expected-Likelihood Weights applied to Local Rearrangements of tree topology) tests that support for the nodes.

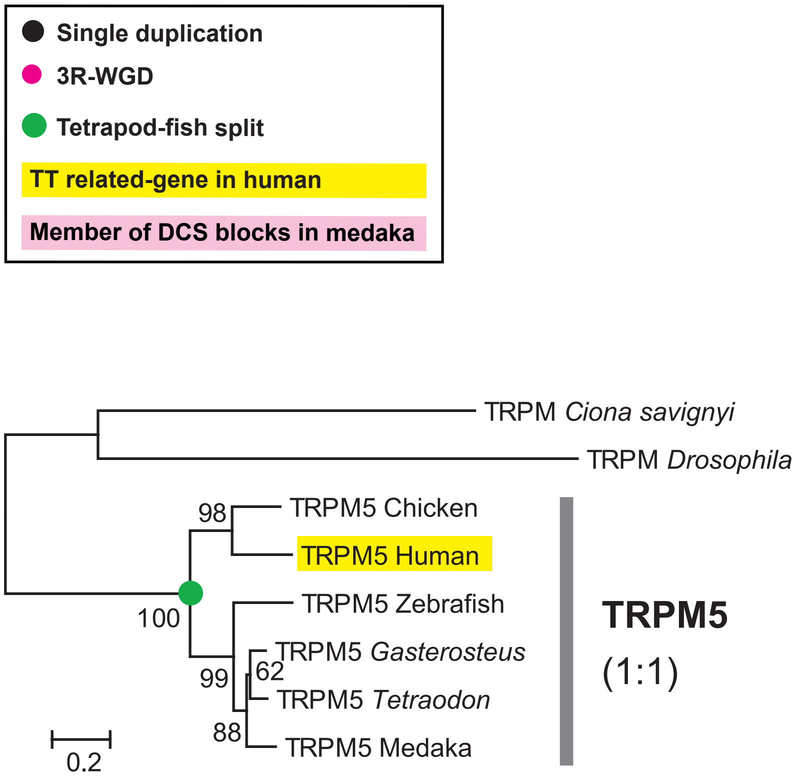

Fig. S35. A molecular phylogeny of TRPM5 (transient receptor potential cation channel, subfamily M, member 5), inferred from maximum-likelihood analysis (865 amino acid sites were used; JTT+Γ). Numbers indicate approximate bootstrap values from 1,000 LR-ELW (the Expected-Likelihood Weights applied to Local Rearrangements of tree topology) tests that support for the

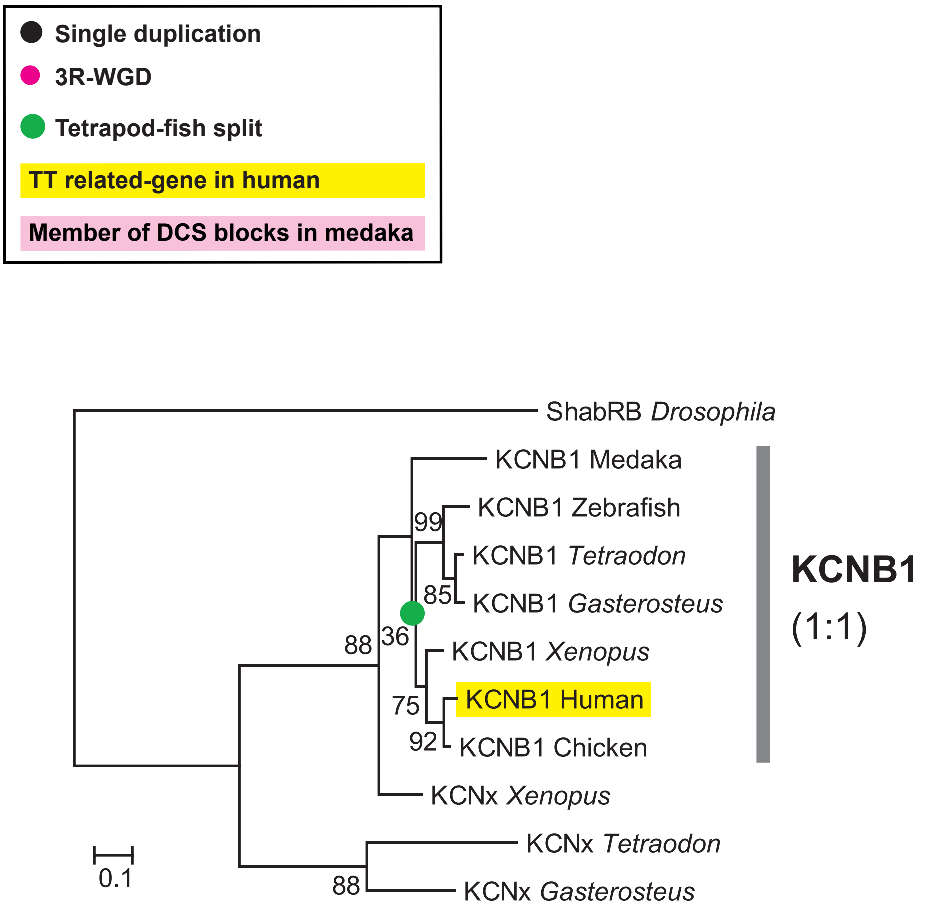

Fig. S36. A molecular phylogeny of KCN (or KCNB1, potassium voltage-gated channel, Shab-related subfamily, member 1), inferred from maximum-likelihood analysis (337 amino acid sites were used; JTT+Γ). Numbers indicate approximate bootstrap values from 1,000 LR-ELW (the Expected-Likelihood Weights applied to Local Rearrangements of tree topology) tests that support

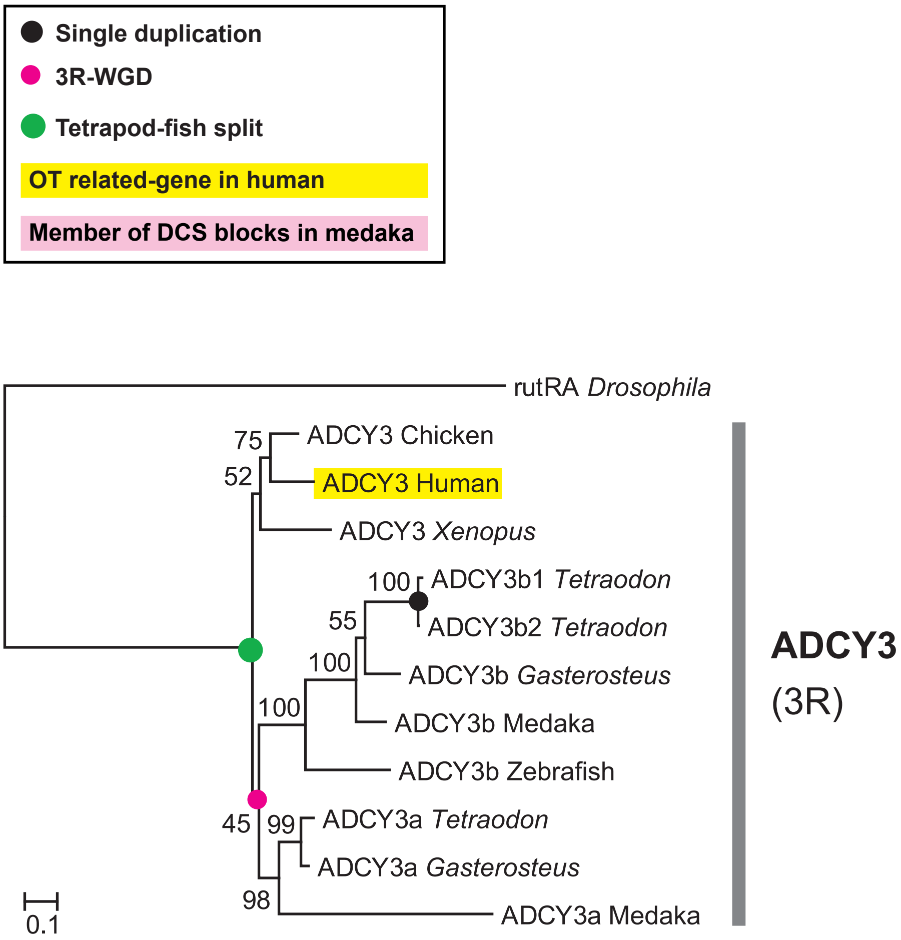

Fig. S37. A molecular phylogeny of AC3 (or ADCY3, adenylate cyclase 3, EC:4.6.1.1), inferred from maximum-likelihood analysis (549 amino acid sites were used; JTT+Γ). Numbers indicate approximate bootstrap values from 1,000 LR-ELW (the Expected-Likelihood Weights applied to Local Rearrangements of tree topology) tests that support for the nodes.

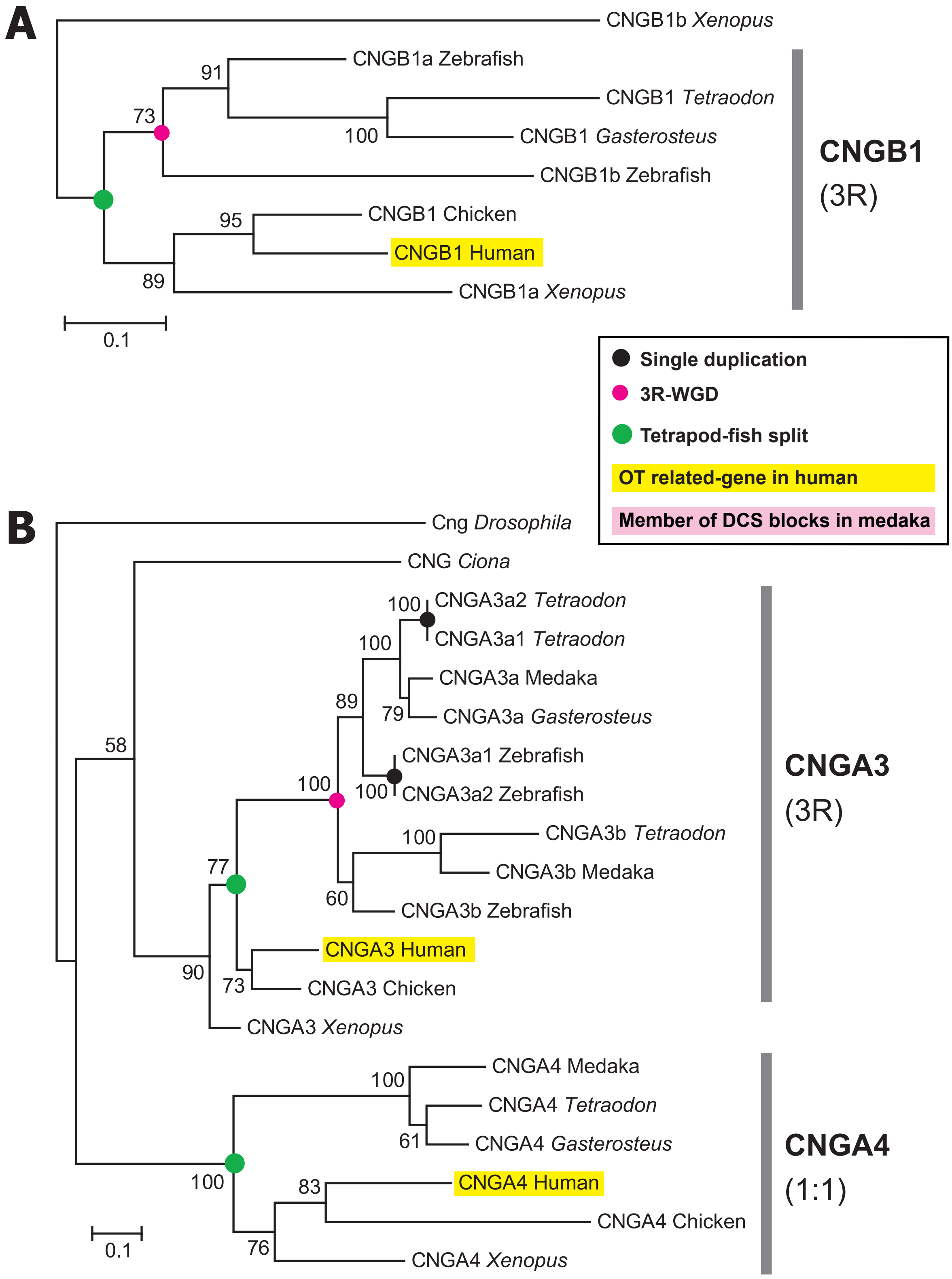

Fig. S38. A molecular phylogeny of CNGA and CNGB (cyclic nucleotide gated channel alpha and beta), inferred from maximum-likelihood analysis (panel **A**: 460 amino acid sites were used with WAG+Γ; panel **B**: 390 amino acid sites were used with JTT+Γ). Numbers indicate approximate bootstrap values from 1,000 LR-ELW (the Expected-Likelihood Weights applied to Local Rearrangements of tree topology) tests that support for the nodes. Conserved synteny around the CNGB1 loci among human and teleosts is shown in Fig. S65.

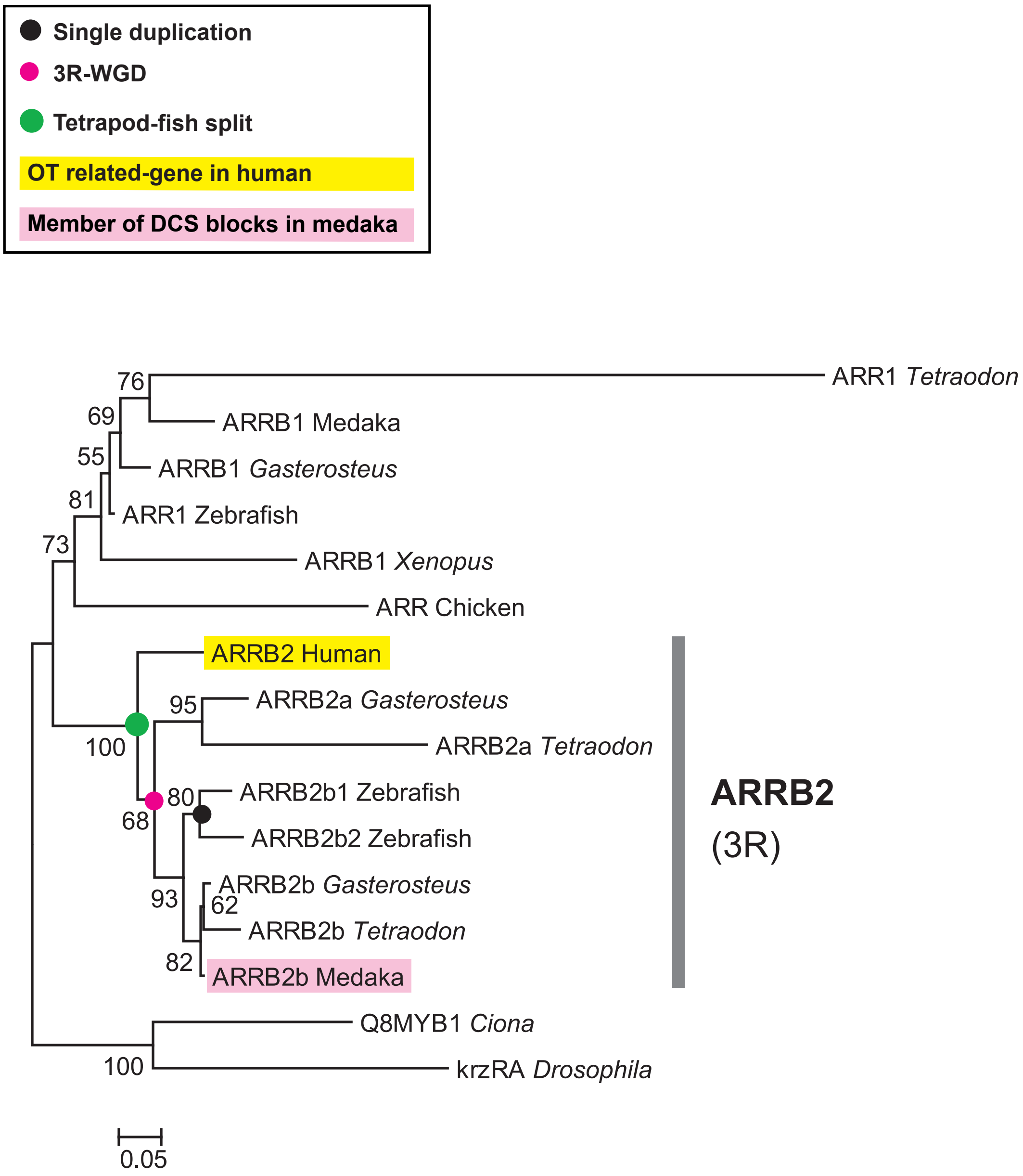

Fig. S39. A molecular phylogeny of ARRB2 (arrestin, beta 2), inferred from maximum-likelihood analysis (297 amino acid sites were used; JTT+Γ). Numbers indicate approximate bootstrap values from 1,000 LR-ELW (the Expected-Likelihood Weights applied to Local Rearrangements of tree topology) tests that support for the nodes.

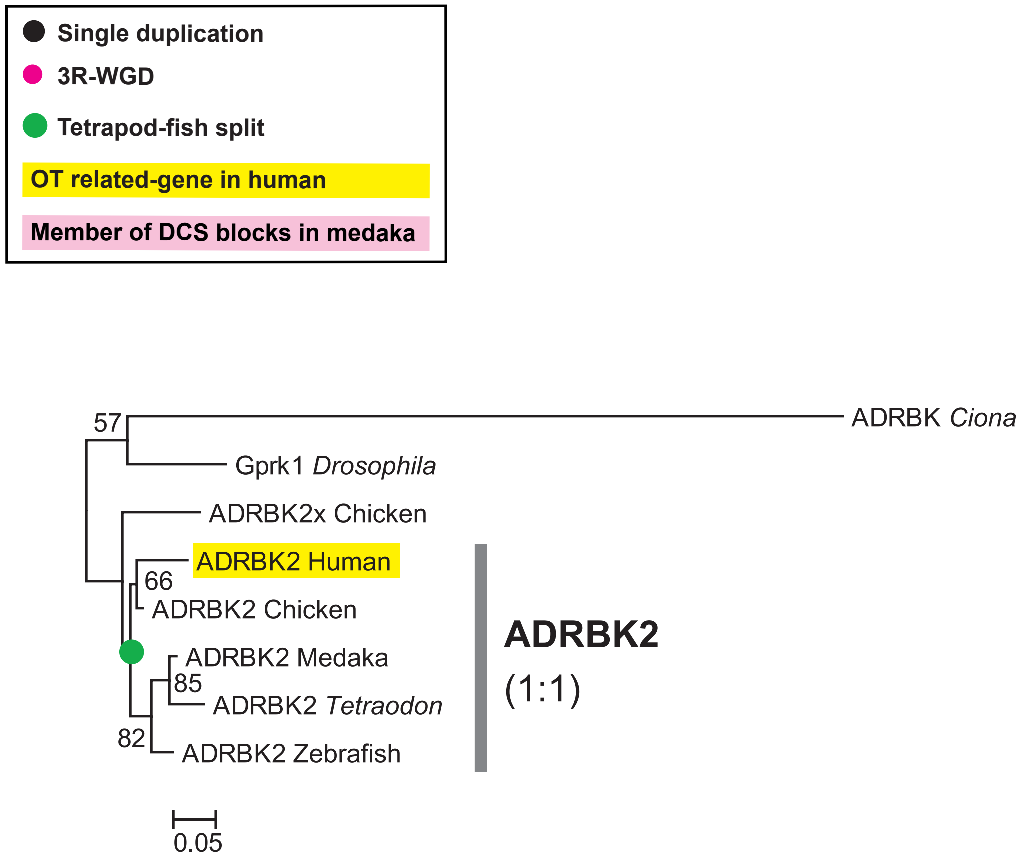

Fig. S40. A molecular phylogeny of GRK (or ADRBK2, adrenergic, beta, receptor kinase 2),, inferred from maximum-likelihood analysis (147 amino acid sites were used; JTT+Γ). Numbers indicate approximate bootstrap values from 1,000 LR-ELW (the Expected-Likelihood Weights applied to Local Rearrangements of tree topology) tests that support for the nodes.

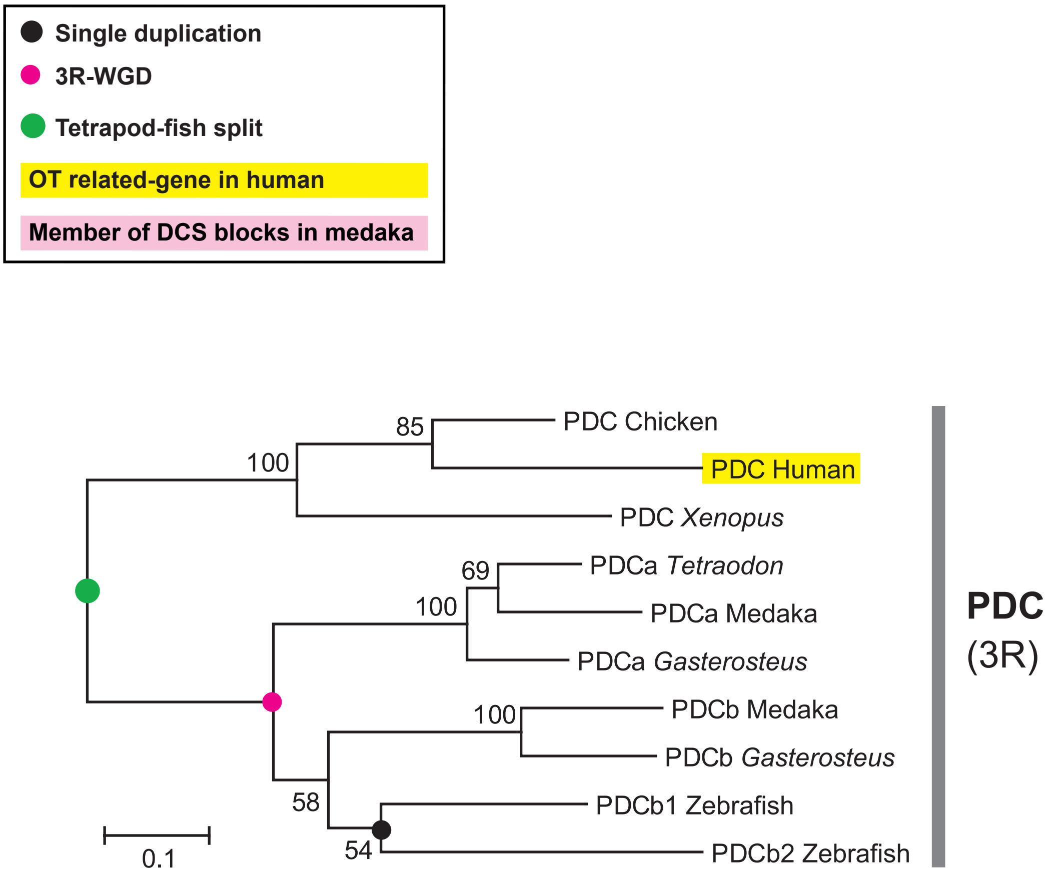

Fig. S41. A molecular phylogeny of PDC (or Phd, phosducin), inferred from maximum-likelihood analysis (638 nucleotide sites were used; TrN+I+Γ). Numbers indicate approximate bootstrap values from 1,000 LR-ELW (the Expected-Likelihood Weights applied to Local Rearrangements of tree topology) tests that support for the nodes. Conserved synteny around the PDC loci among human and teleosts is shown in Fig. S66.

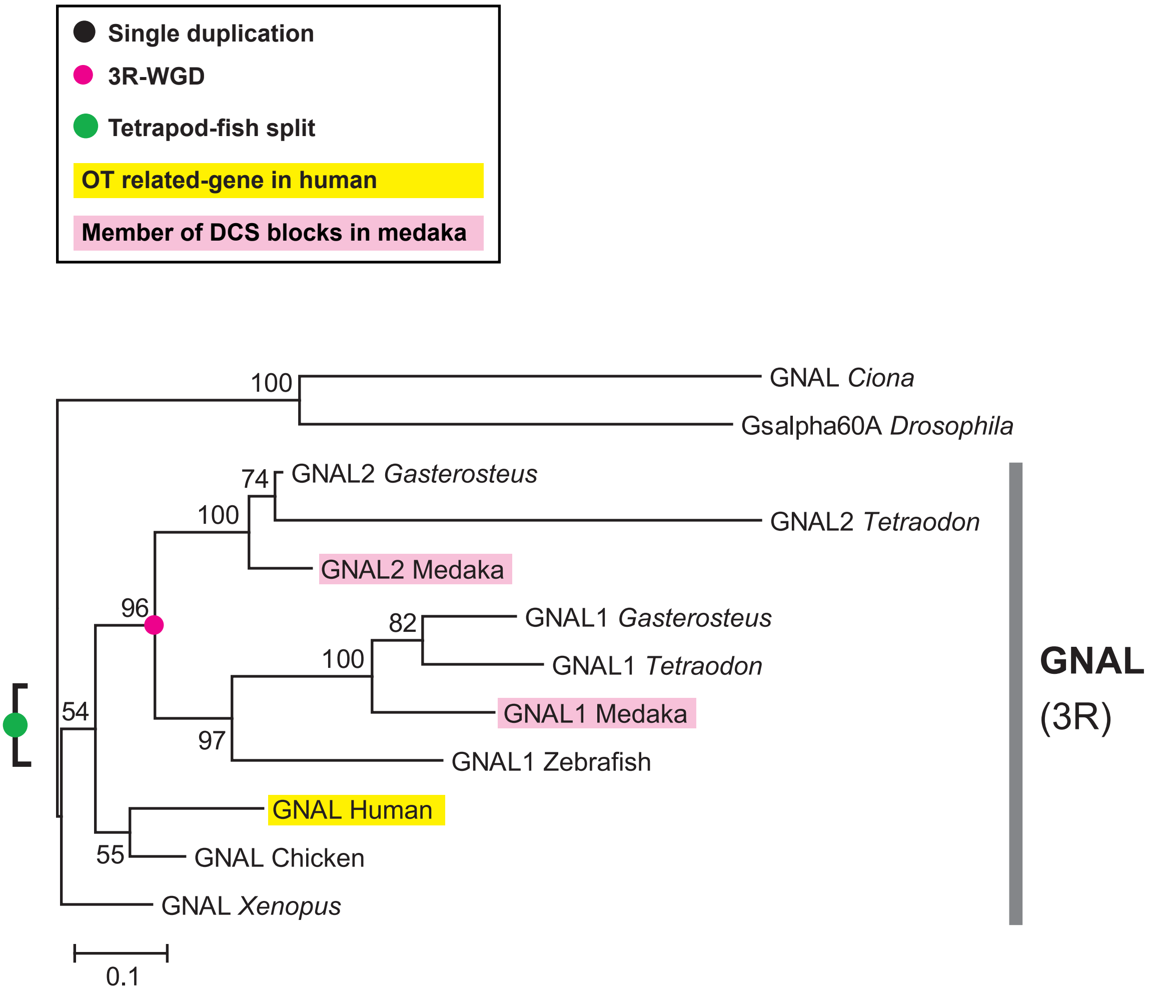

Fig. S42. A molecular phylogeny of GNAL(or Golf, guanine nucleotide binding protein [G protein], alpha activating activity polypeptide, olfactory type), inferred from maximum-likelihood analysis (790 nucleotide sites were used; TrN+Γ). Numbers indicate approximate bootstrap values from 1,000 LR-ELW (the Expected-Likelihood Weights applied to Local Rearrangements of tree topology) tests that support for the nodes.

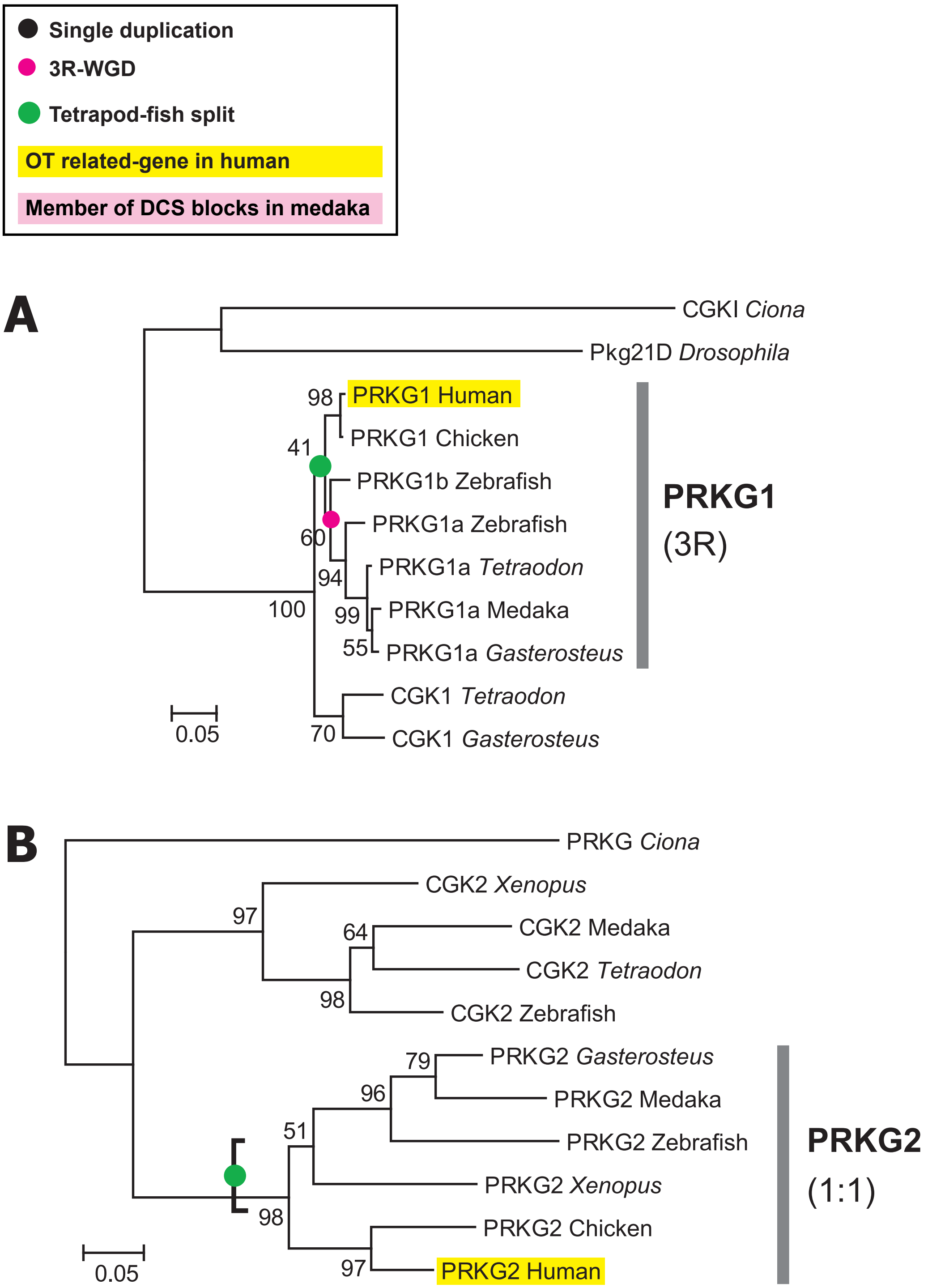

Fig. S43. A molecular phylogeny of PKG (protein kinase, cGMP-dependent, type I and type II), inferred from maximum-likelihood analysis (panel **A**: 327 amino acid sites were used with JTT+Γ; panel **B**: 291 amino acid sites were used with JTT+Γ). Numbers indicate approximate bootstrap values from 1,000 LR-ELW (the Expected-Likelihood Weights applied to Local Rearrangements of tree topology) tests that support for the nodes. Conserved synteny around the PRKG1 loci among human and teleosts is shown in Fig. S67.

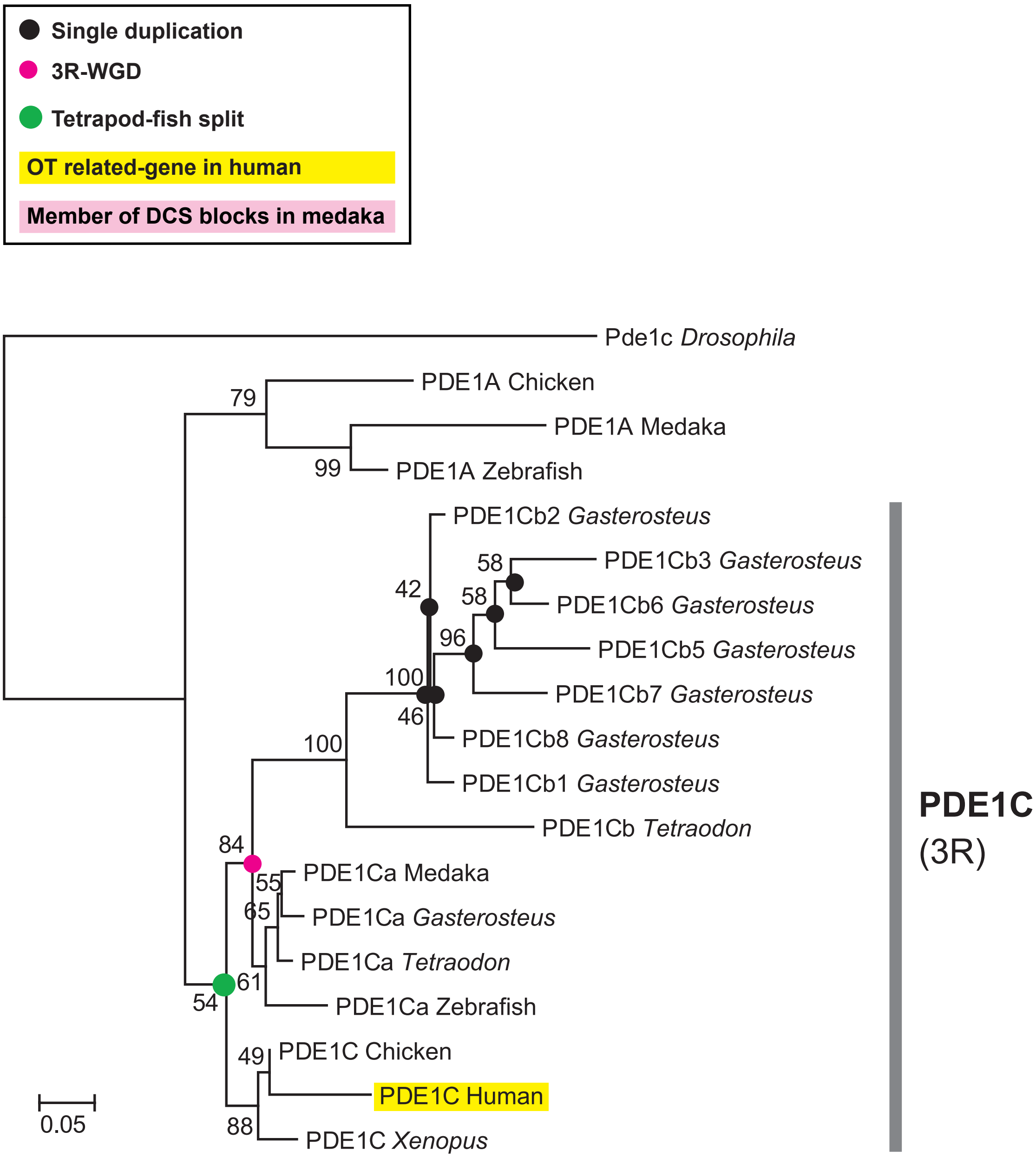

Fig. S44. A molecular phylogeny of PDE1C phosphodiesterase 1C, calmodulin-dependent 70kDa, EC:3.1.4.17), inferred from maximum-likelihood analysis (311 amino acid sites were used; JTT+Γ). Numbers indicate approximate bootstrap values from 1,000 LR-ELW (the Expected-Likelihood Weights applied to Local Rearrangements of tree topology) tests that support for the nodes.

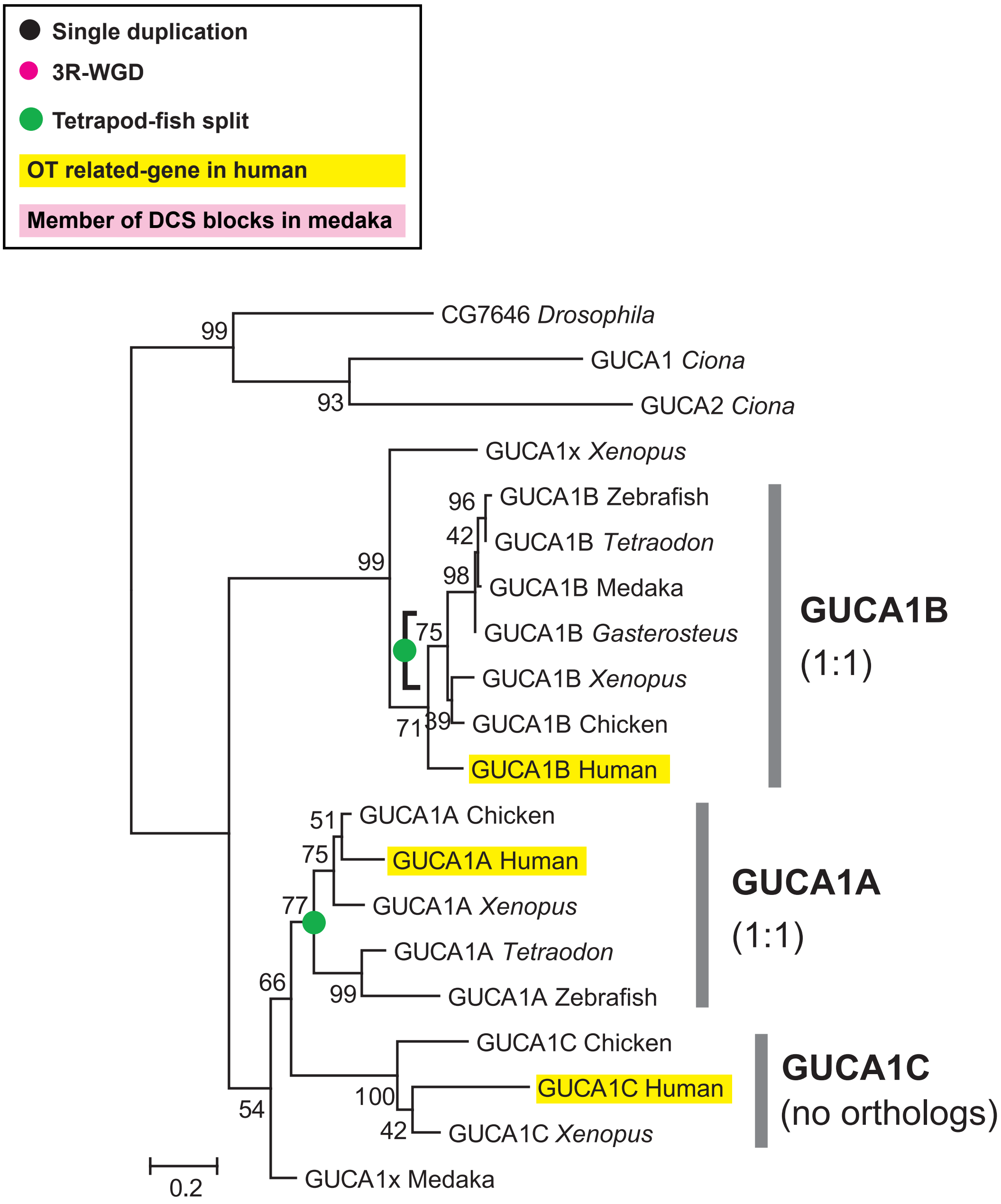

Fig. S45. A molecular phylogeny of GCAP (or GUCA, guanylate cyclase activator), inferred from maximum-likelihood analysis (139 amino acid sites were used; JTT+Γ). Numbers indicate approximate bootstrap values from 1,000 LR-ELW (the Expected-Likelihood Weights applied to Local Rearrangements of tree topology) tests that support for the nodes.

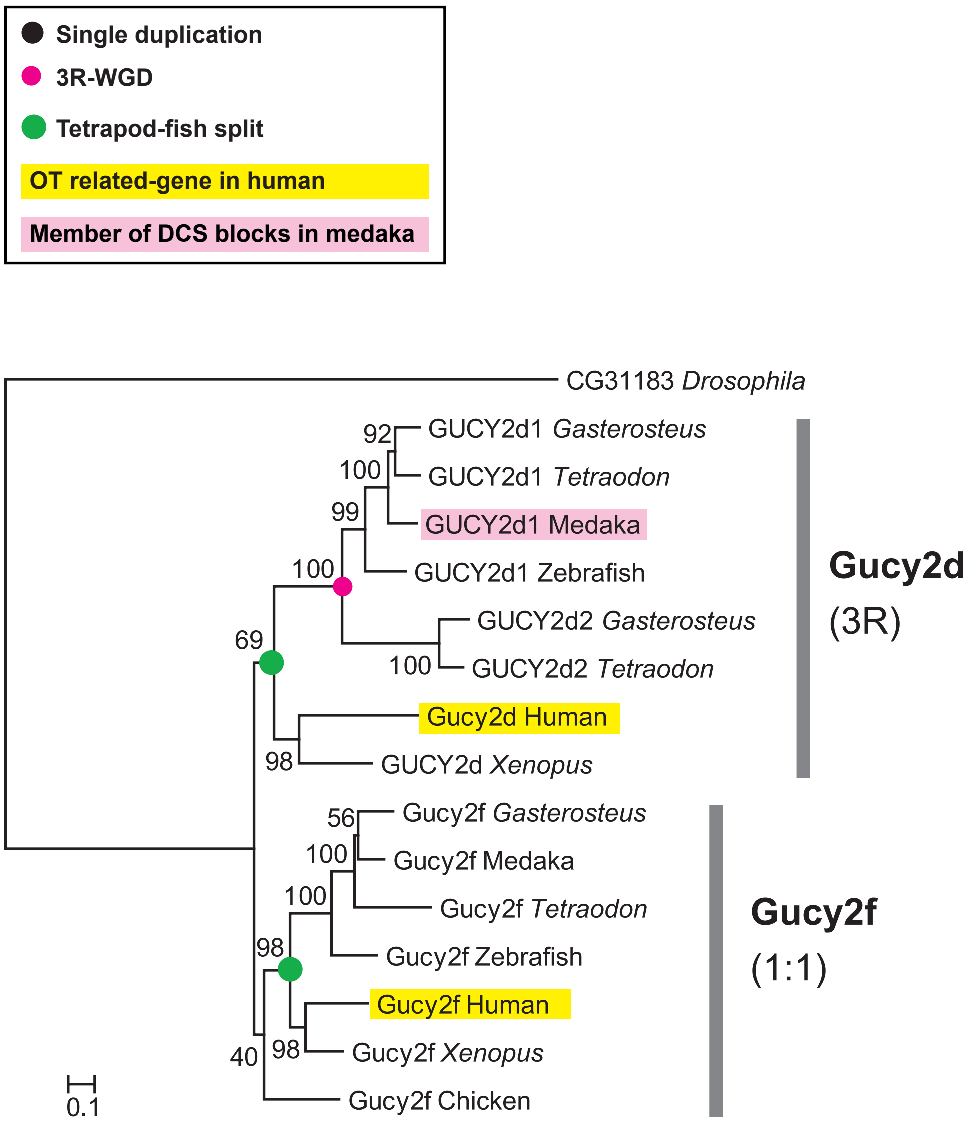

Fig. S46. A molecular phylogeny of pGC (or Gucy, guanylate cyclase), inferred from maximum-likelihood analysis (829 amino acid sites were used; JTT+Γ). Numbers indicate approximate bootstrap values from 1,000 LR-ELW (the Expected-Likelihood Weights applied to Local Rearrangements of tree topology) tests that support for the nodes.

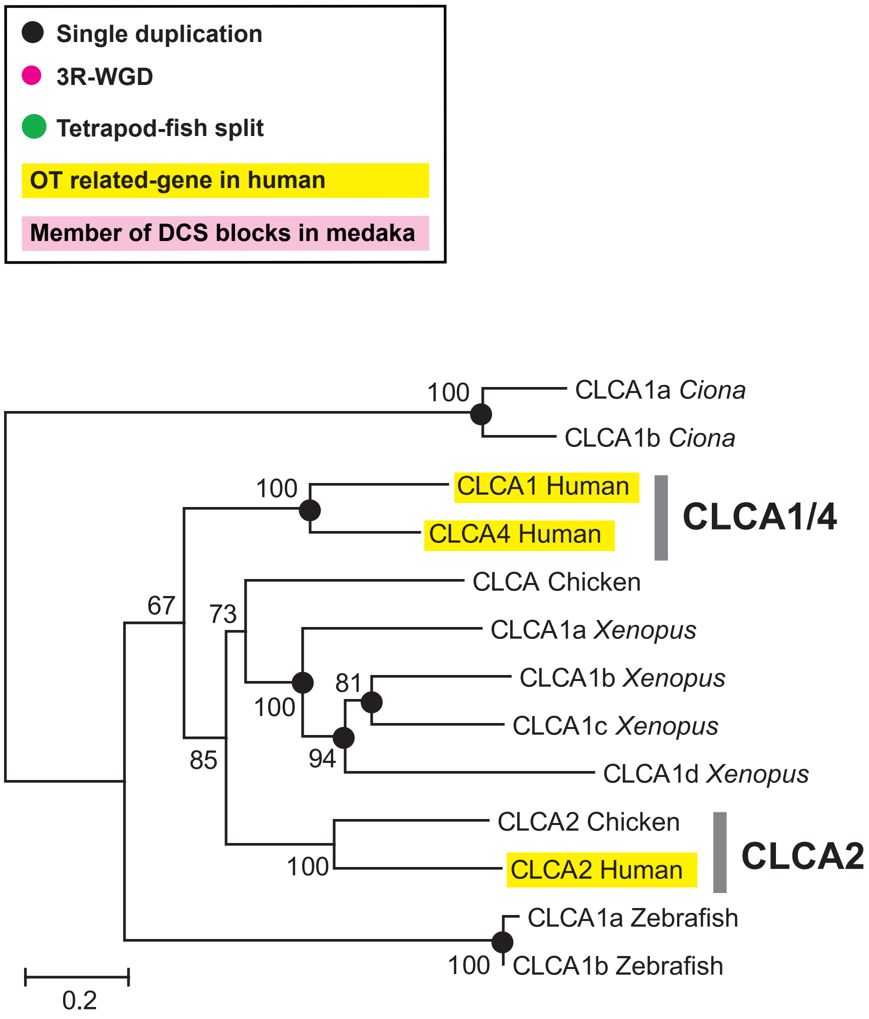

Fig. S47. A molecular phylogeny of CLCA (chloride channel, calcium activated), inferred from maximum-likelihood analysis (536 amino acid sites were used; WAG+Γ). Numbers indicate approximate bootstrap values from 1,000 LR-ELW (the Expected-Likelihood Weights applied to Local Rearrangements of tree topology) tests that support for the nodes.

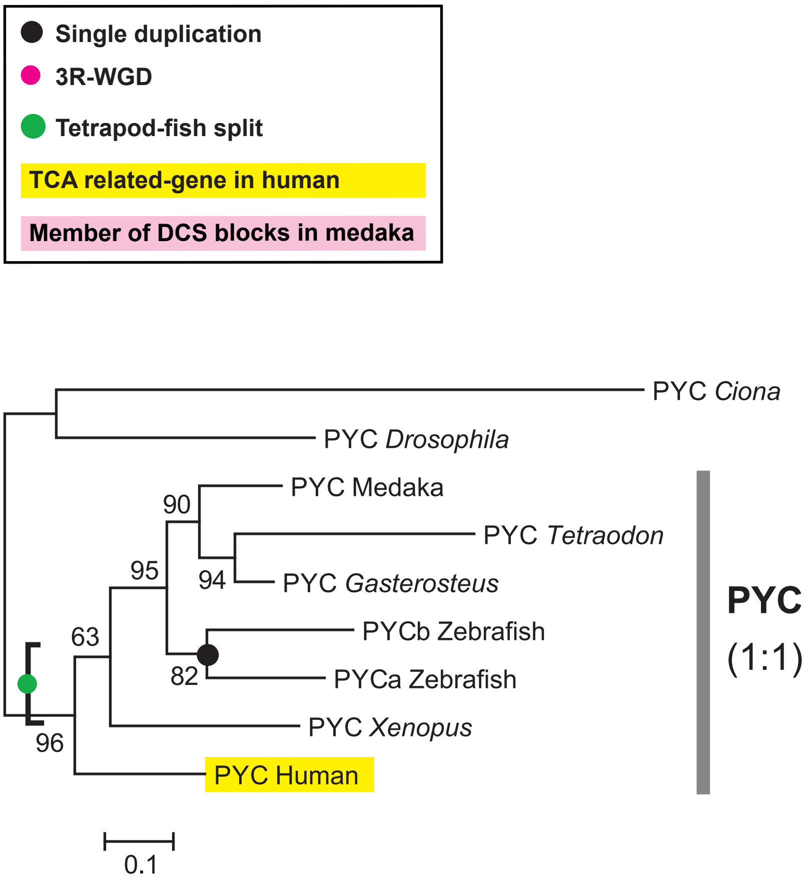

Fig. S48. A molecular phylogeny of PYC (pyruvate carboxylase, E.C. 6.4.1.1), inferred from maximum-likelihood analysis (1290 nucleotide sites were used; GTR+I+Γ). Numbers indicate approximate bootstrap values from 1,000 LR-ELW (the Expected-Likelihood Weights applied to Local Rearrangements of tree topology) tests that support for the nodes.

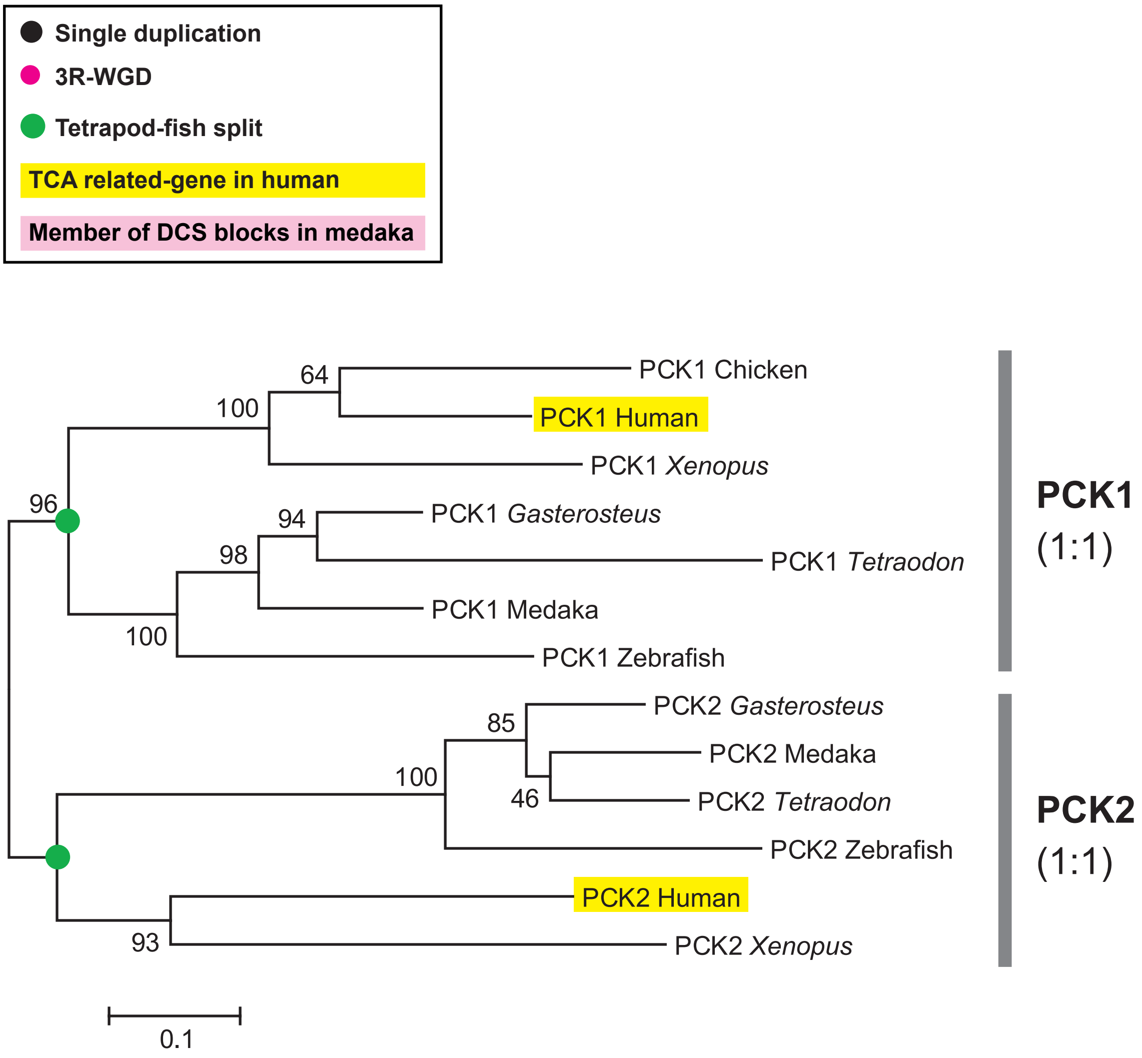

Fig. S49. A molecular phylogeny of PCK(phosphoenolpyruvate carboxykinase, soluble, E.C. 4.1.1.32), inferred from maximum-likelihood analysis (1810 nucleotide sites were used; GTR+I+Γ). Numbers indicate approximate bootstrap values from 1,000 LR-ELW (the Expected-Likelihood Weights applied to Local Rearrangements of tree topology) tests that support for the

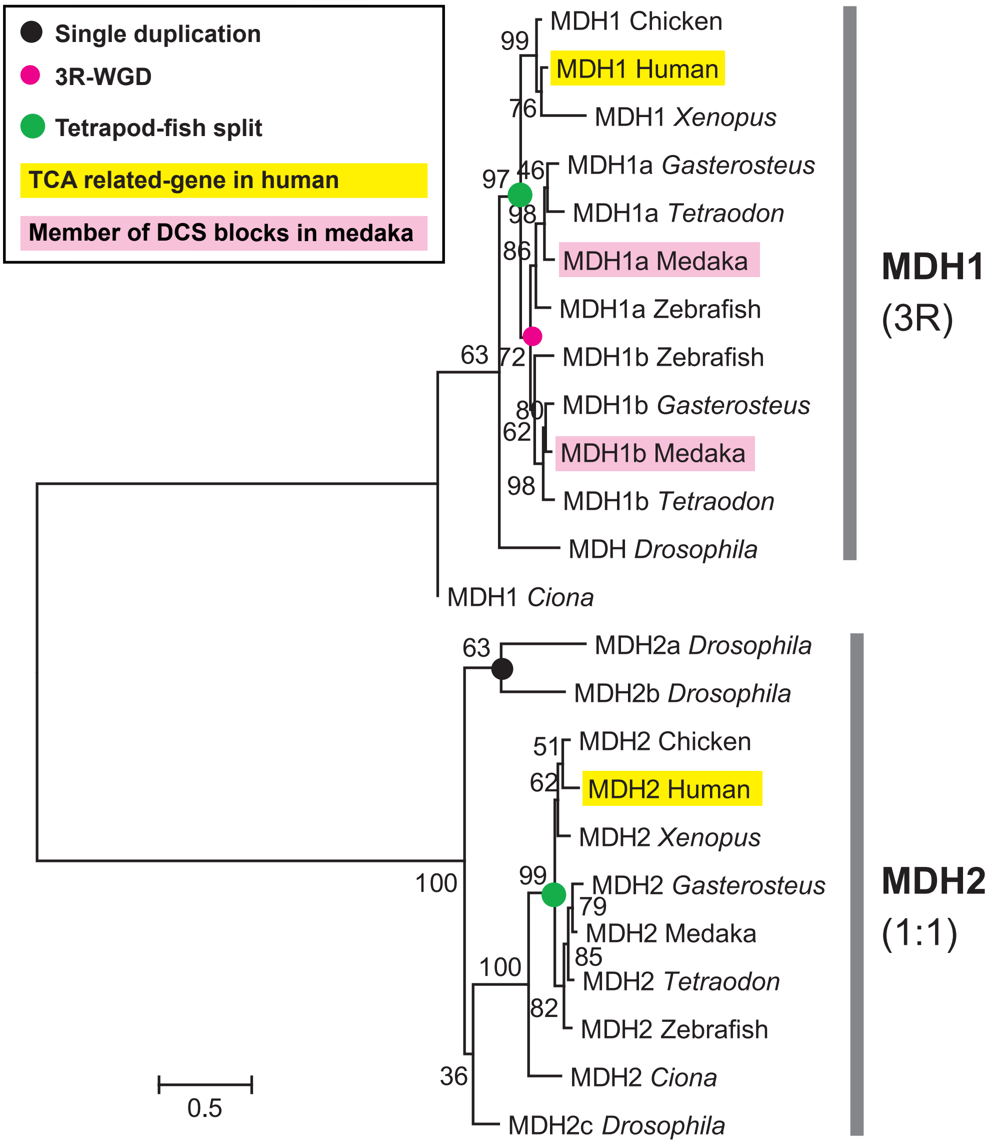

Fig. S50. A molecular phylogeny of MDH (malate dehydrogenase 1, NAD, soluble, EC:1.1.1.37),, inferred from maximum-likelihood analysis (245 amino acid sites were used; WAG+Γ). Numbers indicate approximate bootstrap values from 1,000 LR-ELW (the Expected-Likelihood Weights applied to Local Rearrangements of tree topology) tests that support for the nodes.

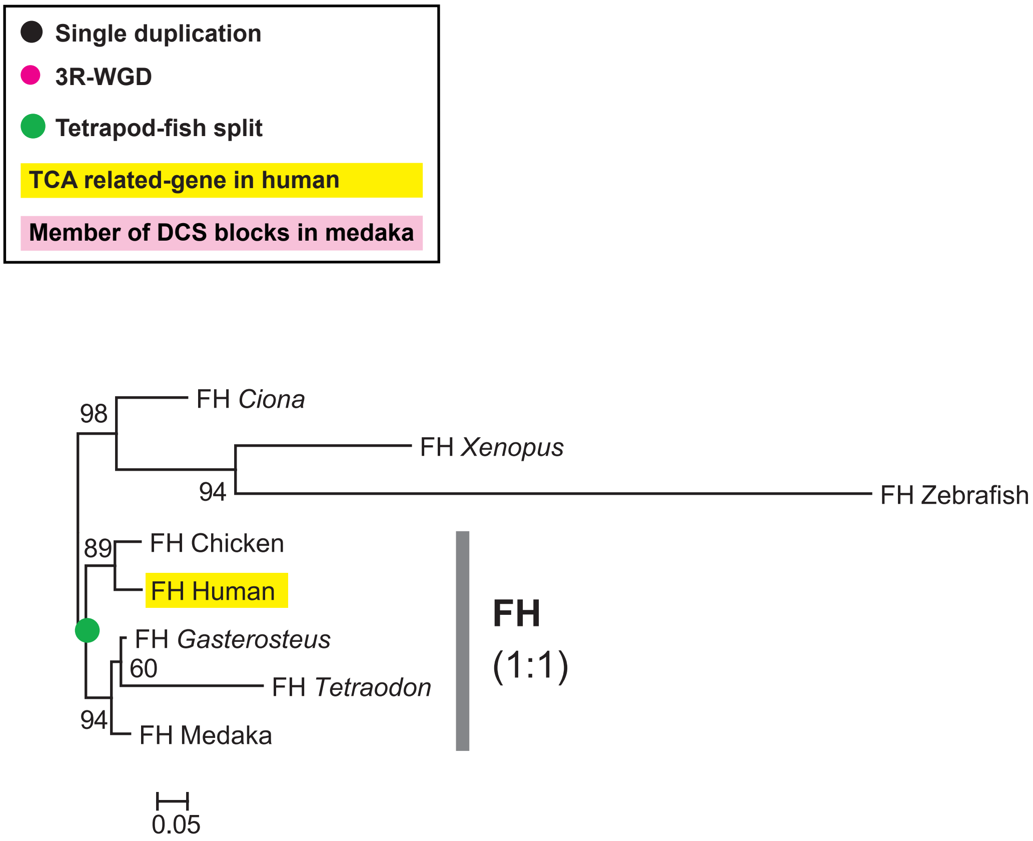

Fig. S51. A molecular phylogeny of FH (fumarate hydratase, E.C. 4.2.1.2), inferred from maximum-likelihood analysis (361 amino acid sites were used; WAG+Γ). Numbers indicate approximate bootstrap values from 1,000 LR-ELW (the Expected-Likelihood Weights applied to Local Rearrangements of tree topology) tests that support for the nodes.

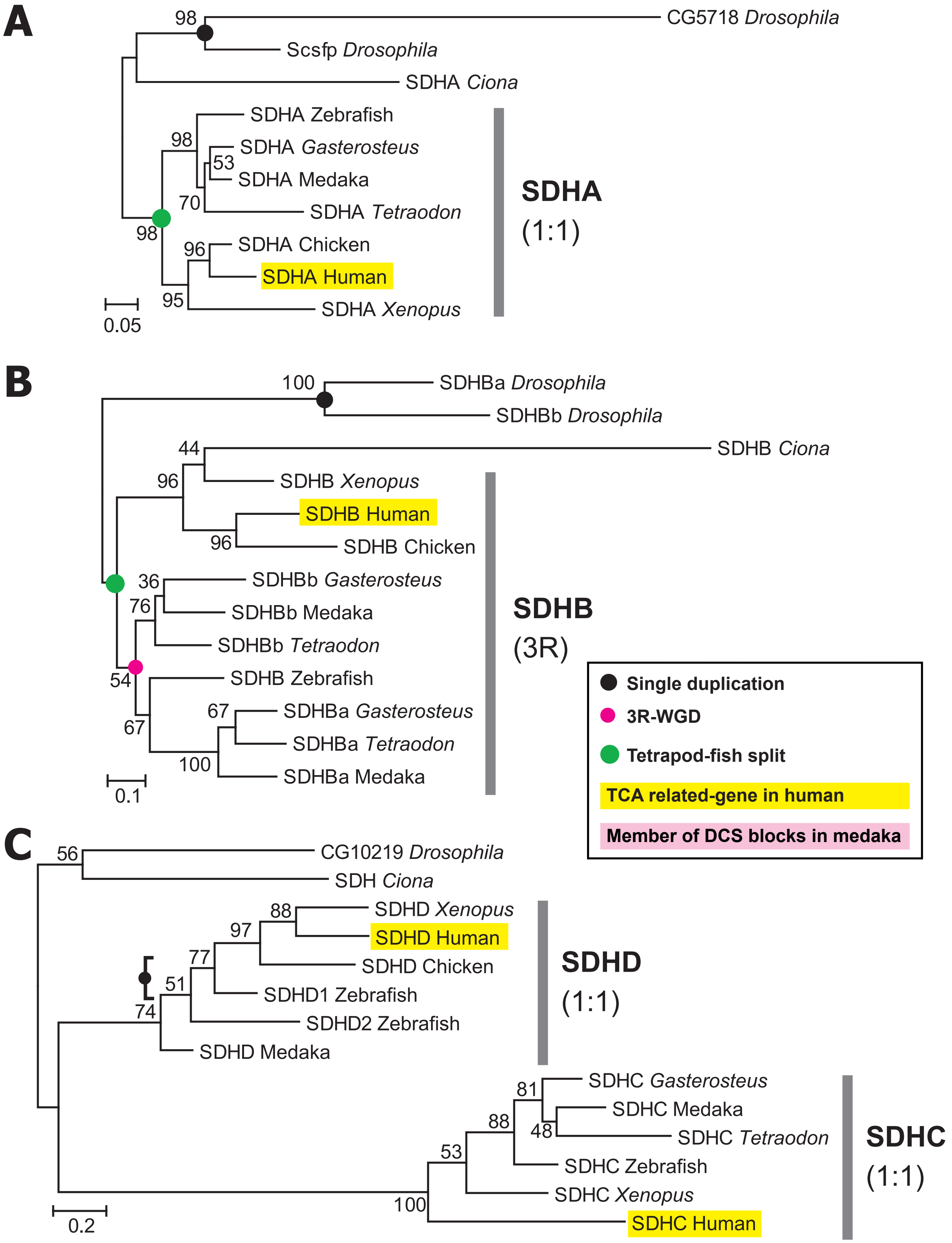

Fig. S52. A molecular phylogeny of SDH (succinate dehydrogenase complex, E.C. 1.3.5.1), inferred from maximum-likelihood analysis (panel A: 635 amino acid sites were used with WAG+Γ; panel B: 744 nucleotide sites were used with TrN+I+Γ; panel C: 343 nucleotide sites were used with HKY+Γ). Numbers indicate approximate bootstrap values from 1,000 LR-ELW (the Expected-Likelihood Weights applied to Local Rearrangements of tree topology) tests that support for the nodes.

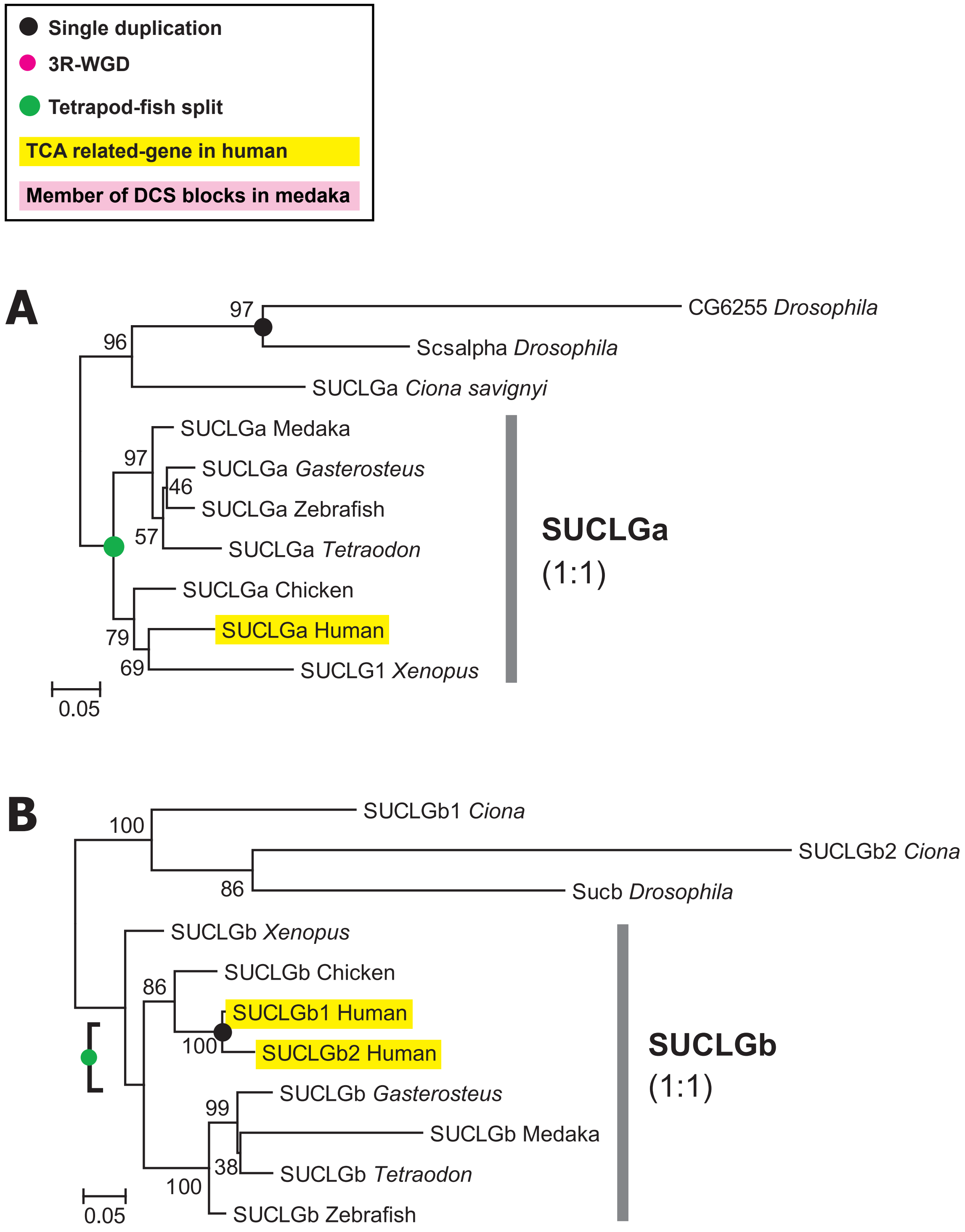

Fig. S53. A molecular phylogeny of SUCLG (succinate-CoA ligase, GDP-forming, E.C. 6.2.1.4), inferred from maximum-likelihood analysis (panel A: 298 amino acid sites were used with WAG+Γ; panel B: 345 amino acid sites were used with WAG+Γ). Numbers indicate approximate bootstrap values from 1,000 LR-ELW (the Expected-Likelihood Weights applied to Local Rearrangements of tree topology) tests that support for the nodes.

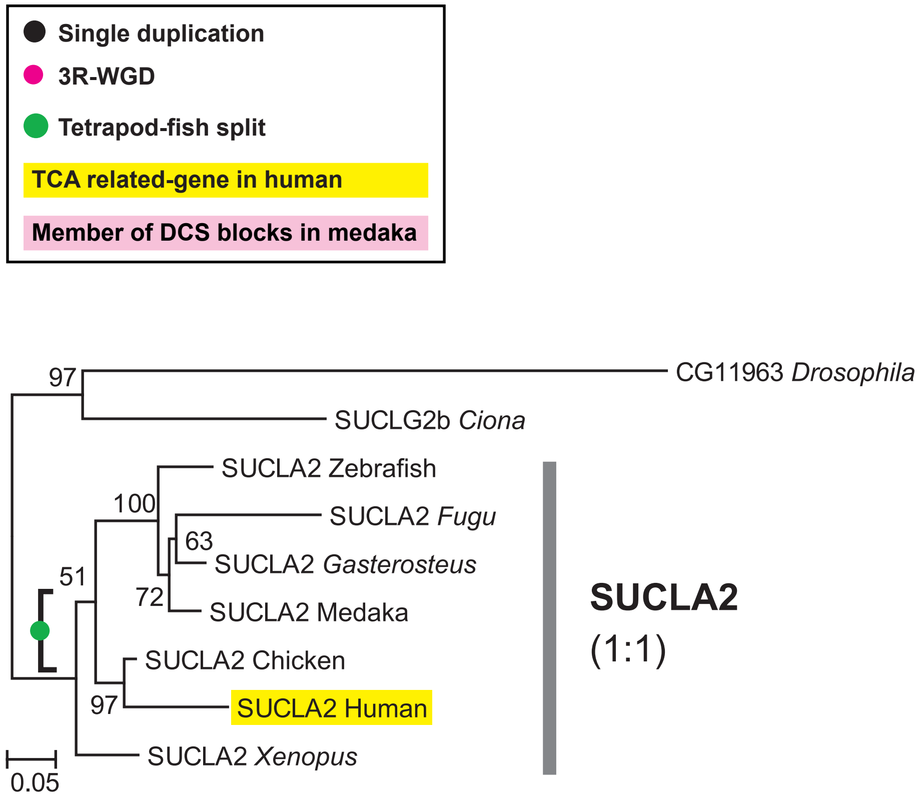

Fig. S54. A molecular phylogeny of SUCLA2 (succinate-CoA ligase, ADP-forming, E.C. 6.2.1.5), inferred from maximum-likelihood analysis (419 amino acid sites were used; WAG+Γ). Numbers indicate approximate bootstrap values from 1,000 LR-ELW (the Expected-Likelihood Weights applied to Local Rearrangements of tree topology) tests that support for the nodes.

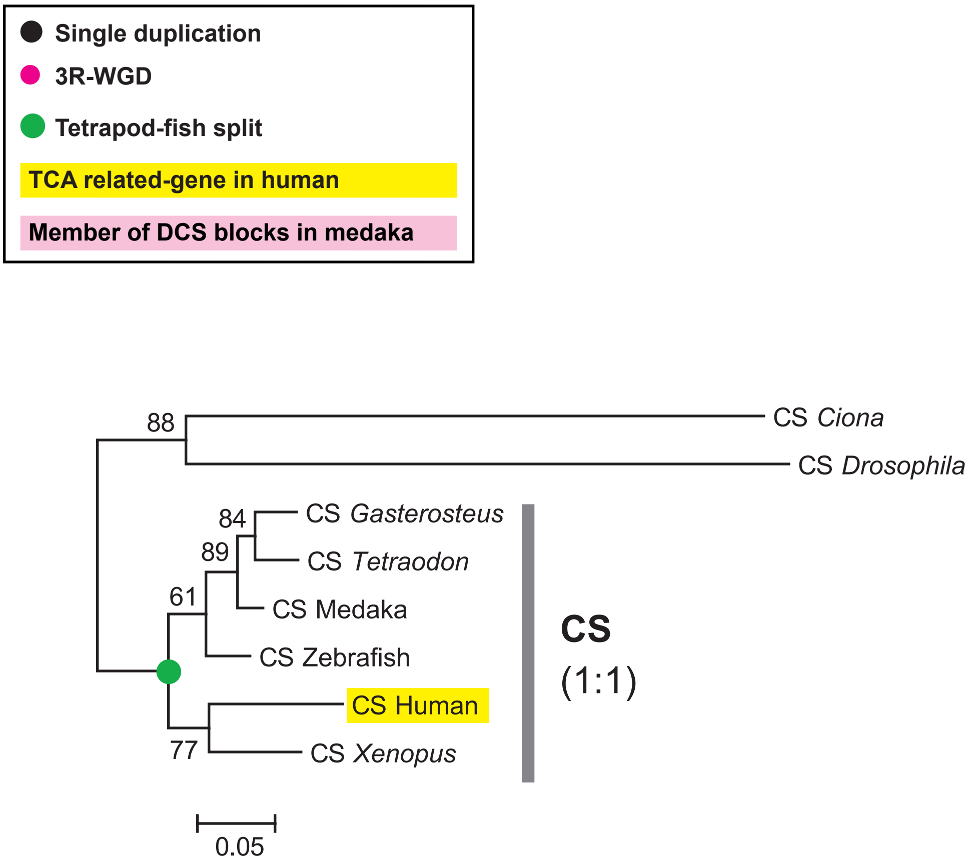

Fig. S55. A molecular phylogeny of CS (citrate synthase, E.C. 2.3.3.1), inferred from maximum-likelihood analysis (461amino acid sites were used; WAG+Γ). Numbers indicate approximate bootstrap values from 1,000 LR-ELW (the Expected-Likelihood Weights applied to Local Rearrangements of tree topology) tests that support for the nodes.

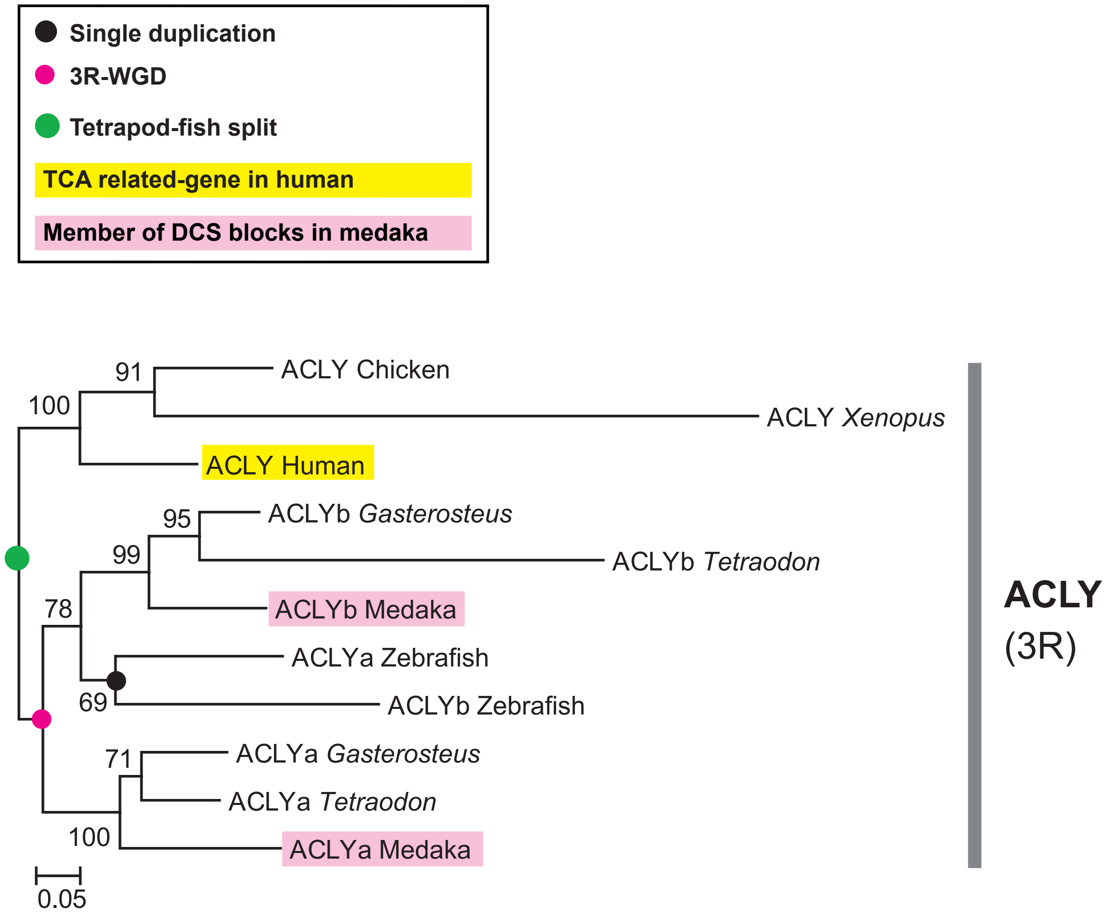

Fig. S56. A molecular phylogeny of ACLY (ATP citrate lyase, E.C. 2.3.3.8), inferred from maximum-likelihood analysis (1601 nucleotide sites were used; GTR+Γ). Numbers indicate approximate bootstrap values from 1,000 LR-ELW (the Expected-Likelihood Weights applied to Local Rearrangements of tree topology) tests that support for the nodes.

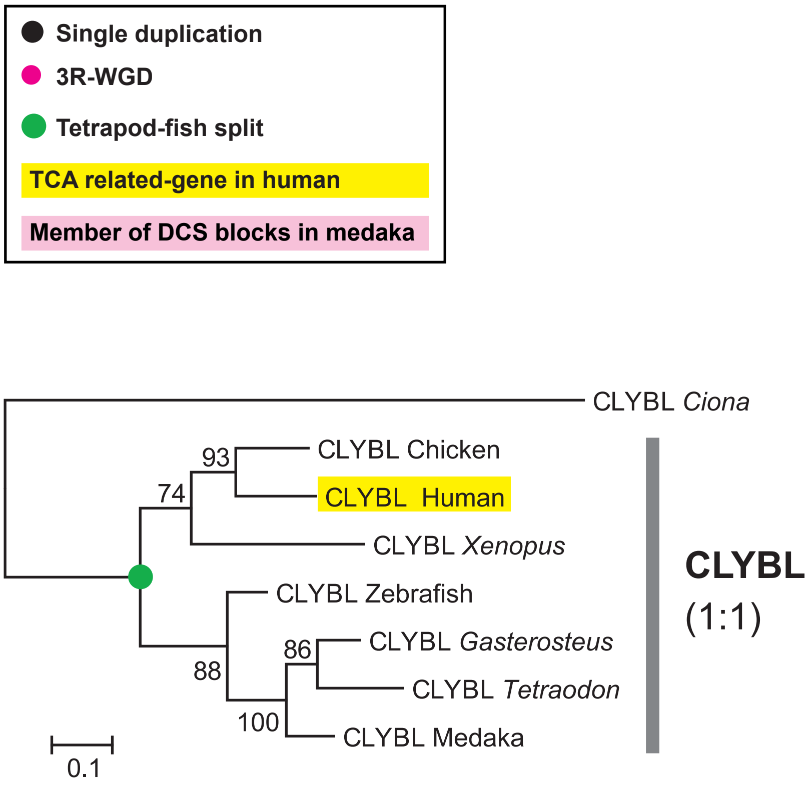

Fig. S57. A molecular phylogeny of CLYBL (citrate lyase beta like, E.C. 4.1.3.6), inferred from maximum-likelihood analysis (257 amino acid sites were used; JTT+Γ). Numbers indicate approximate bootstrap values from 1,000 LR-ELW (the Expected-Likelihood Weights applied to Local Rearrangements of tree topology) tests that support for the nodes.

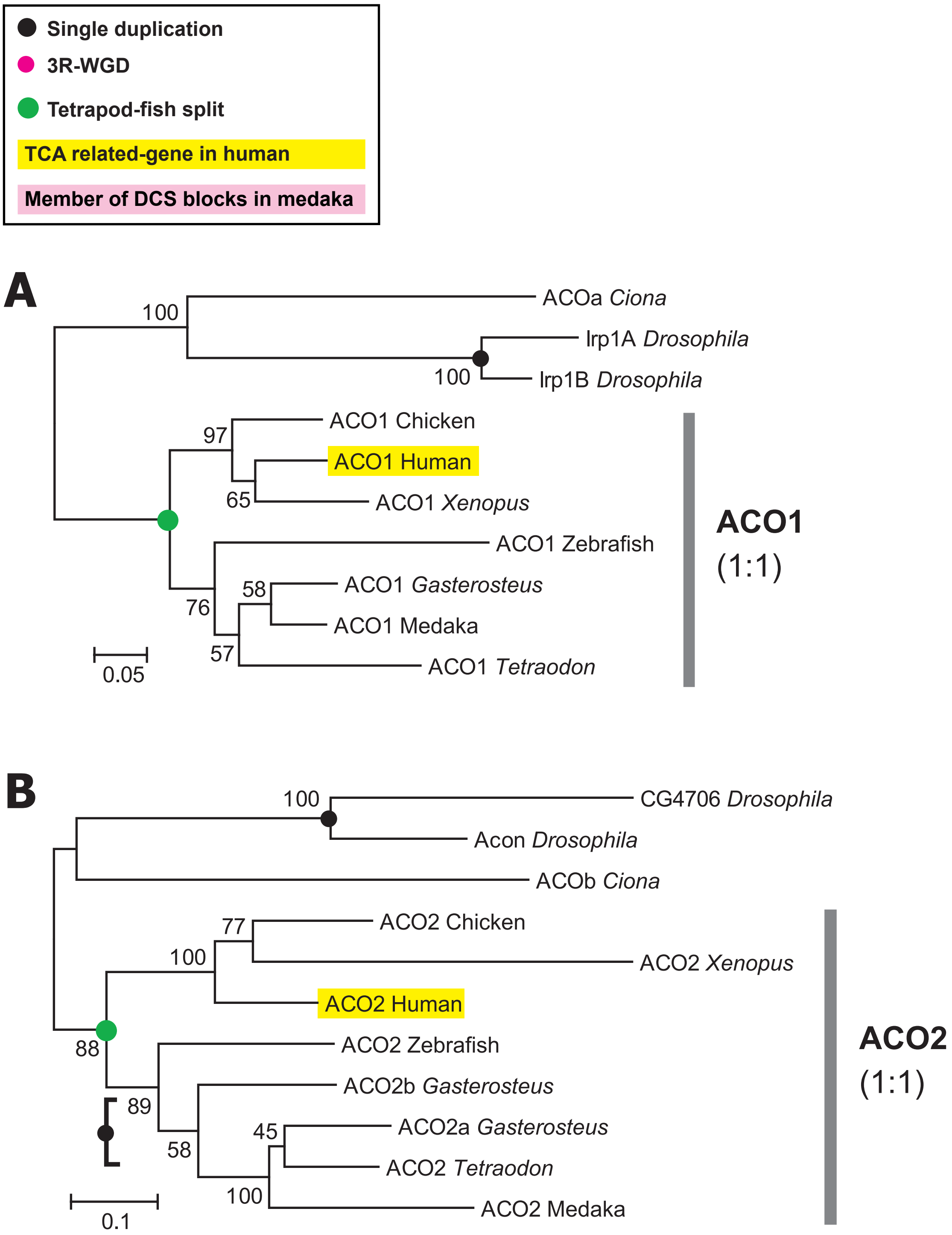

Fig. S58. A molecular phylogeny of ACO (aconitase, E.C. 4.2.1.3), inferred from maximum-likelihood analysis (panel **A**: 488 amino acid sites were used with WAG+Γ; panel **B**: 2184 nucleotide sites were used with GTR+I+Γ). Numbers indicate approximate bootstrap values from 1,000 LR-ELW (the Expected-Likelihood Weights applied to Local Rearrangements of tree topology) tests that support for the nodes. Conserved synteny around the ACO2 loci among human and teleosts is shown in Fig. S68.

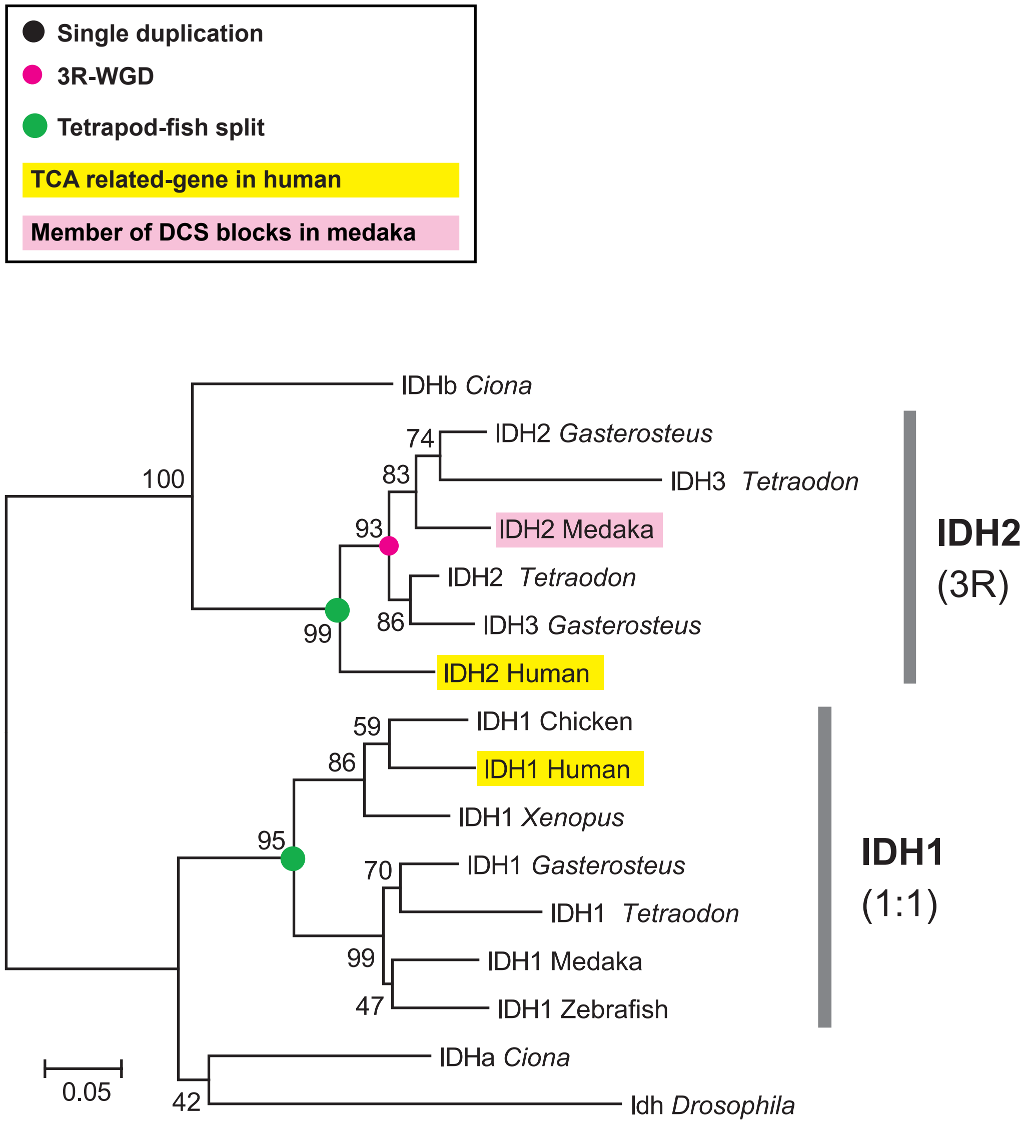

Fig. S59. A molecular phylogeny of E.C. 1.1.1.42 IDH1 (isocitrate dehydrogenase 1 [NADP+], soluble, E.C. 1.1.1.42), inferred from maximum-likelihood analysis (380 amino acid sites were used; WAG+Γ). Numbers indicate approximate bootstrap values from 1,000 LR-ELW (the Expected-Likelihood Weights applied to Local Rearrangements of tree topology) tests that support for the nodes.

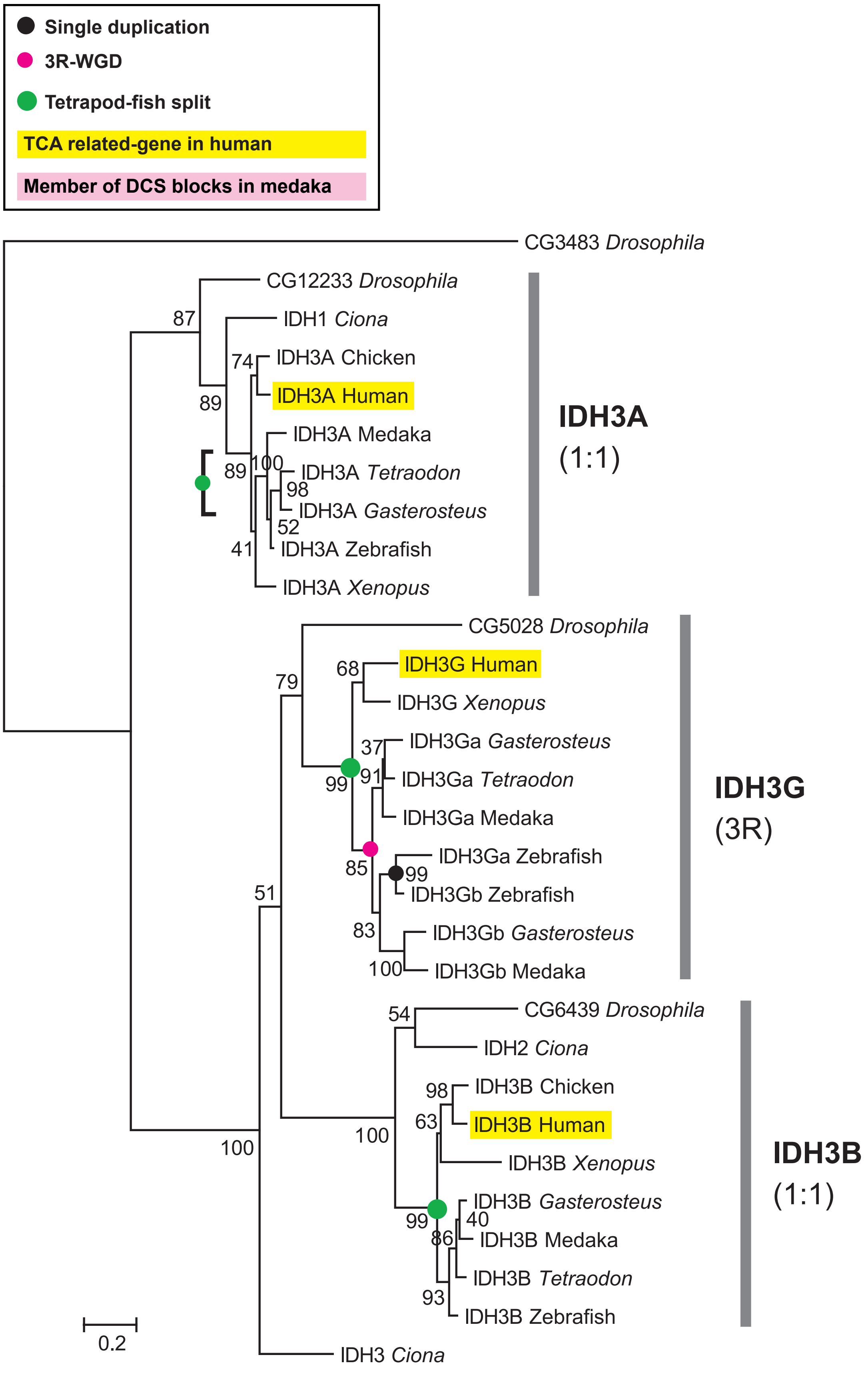

Fig. S60. A molecular phylogeny of IDH3 (isocitrate dehydrogenase 3 [NAD+], E.C. 1.1.1.41), inferred from maximum-likelihood analysis (303 amino acid sites were used; WAG+Γ). Numbers indicate approximate bootstrap values from 1,000 LR-ELW (the Expected-Likelihood Weights applied to Local Rearrangements of tree topology) tests that support for the nodes.

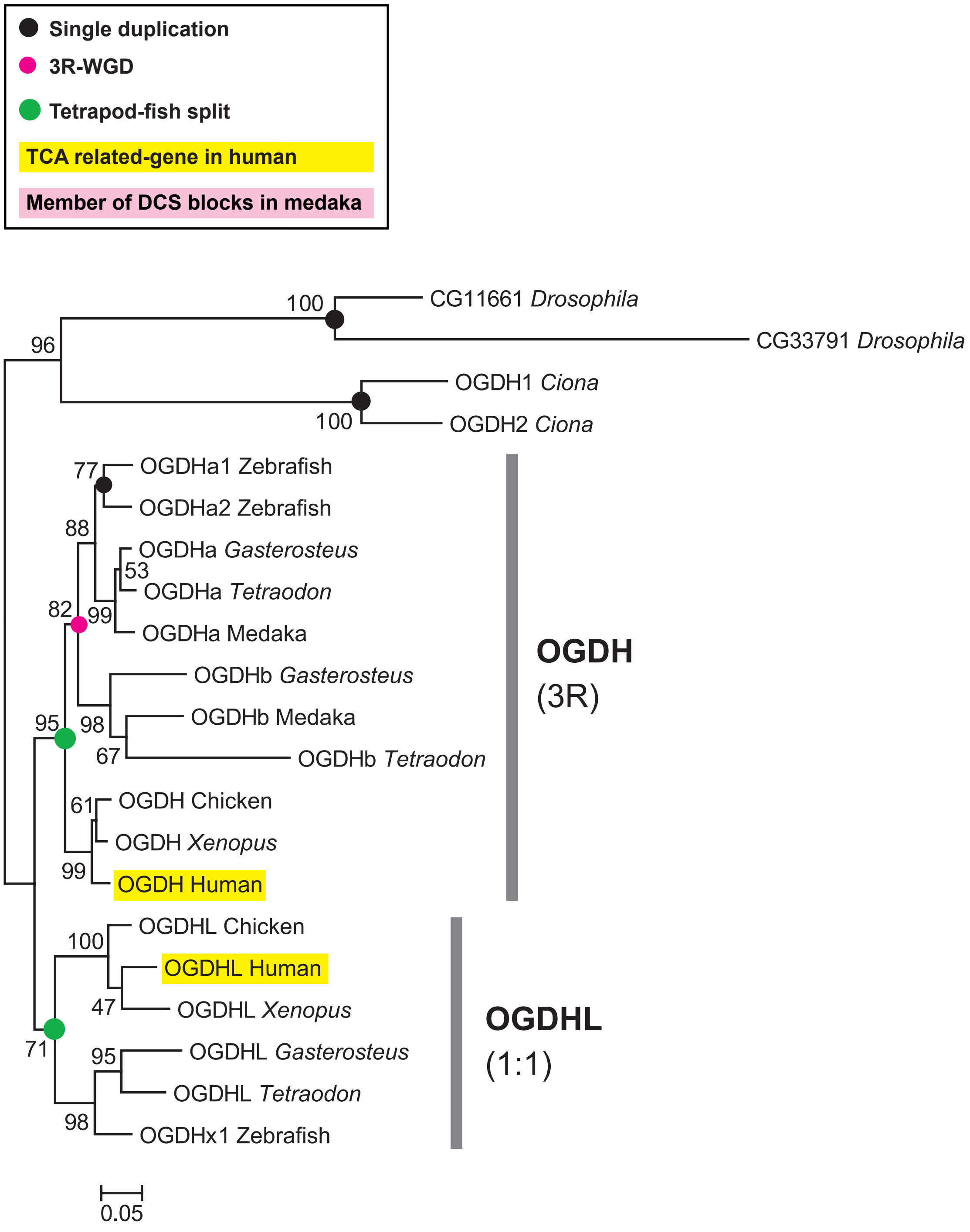

Fig. S61 A molecular phylogeny of OGDH (oxoglutarate dehydrogenase [lipoamide], E.C. 1.2.4.2), inferred from maximum-likelihood analysis (353 amino acid sites were used; JTT+Γ). Numbers indicate approximate bootstrap values from 1,000 LR-ELW (the Expected-Likelihood Weights applied to Local Rearrangements of tree topology) tests that support for the nodes.

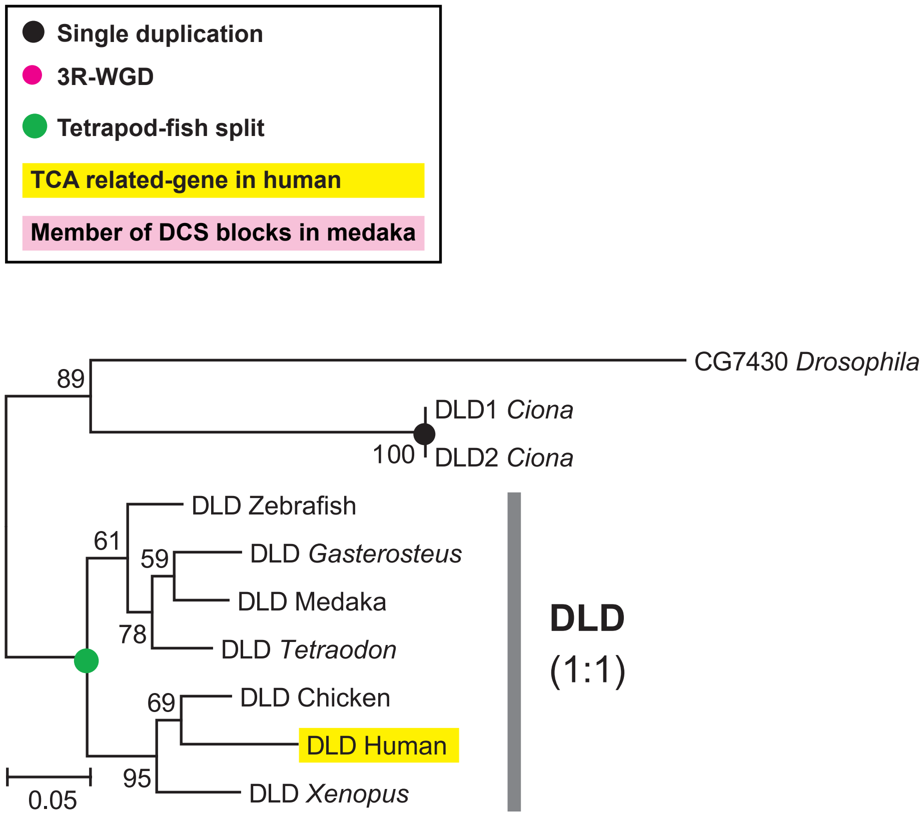

Fig. S62. A molecular phylogeny of DLD (dihydrolipoamide dehydrogenase, E.C. 1.8.1.4), inferred from maximum-likelihood analysis (393 amino acid sites were used; WAG+Γ). Numbers indicate approximate bootstrap values from 1,000 LR-ELW (the Expected-Likelihood Weights applied to Local Rearrangements of tree topology) tests that support for the nodes.

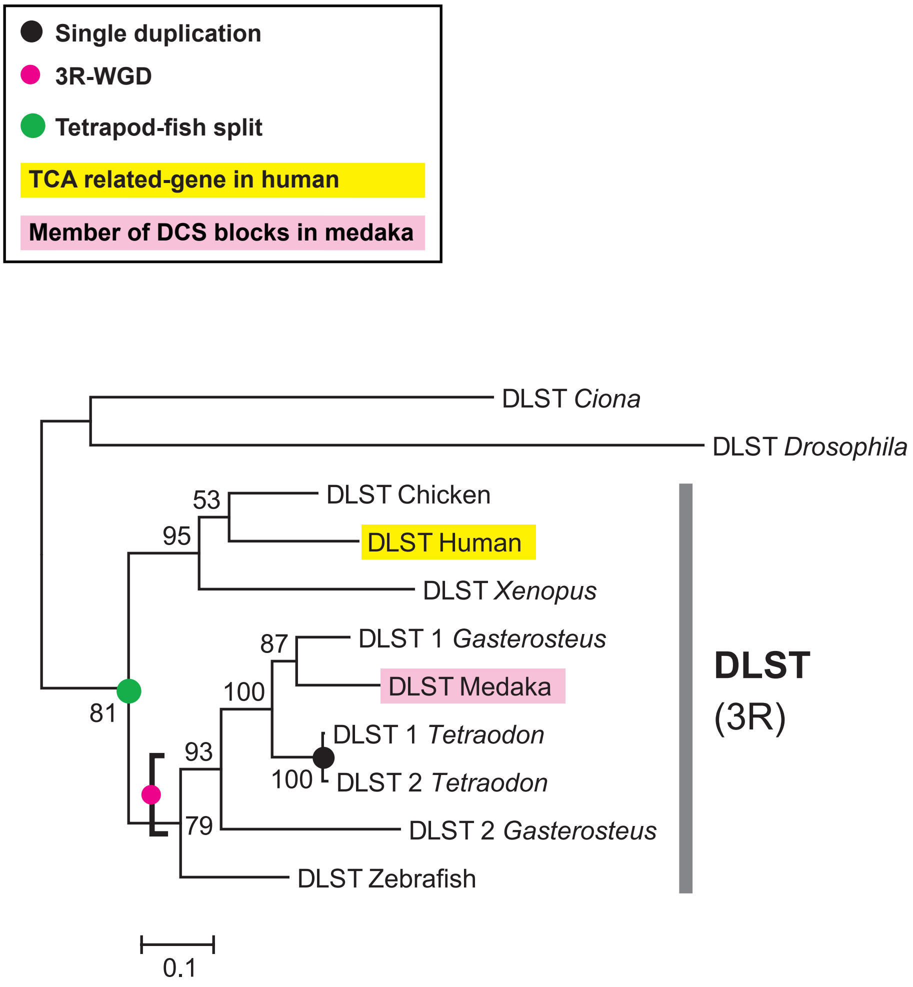

Fig. S63. A molecular phylogeny of DLST (dihydrolipoamide S-succinyltransferase, E2 component of 2-oxo-glutarate complex, E.C. 2.3.1.61), inferred from maximum-likelihood analysis (1170 nucleotide sites were used; GTR+I+Γ). Numbers indicate approximate bootstrap values from 1,000 LR-ELW (the Expected-Likelihood Weights applied to Local Rearrangements of tree

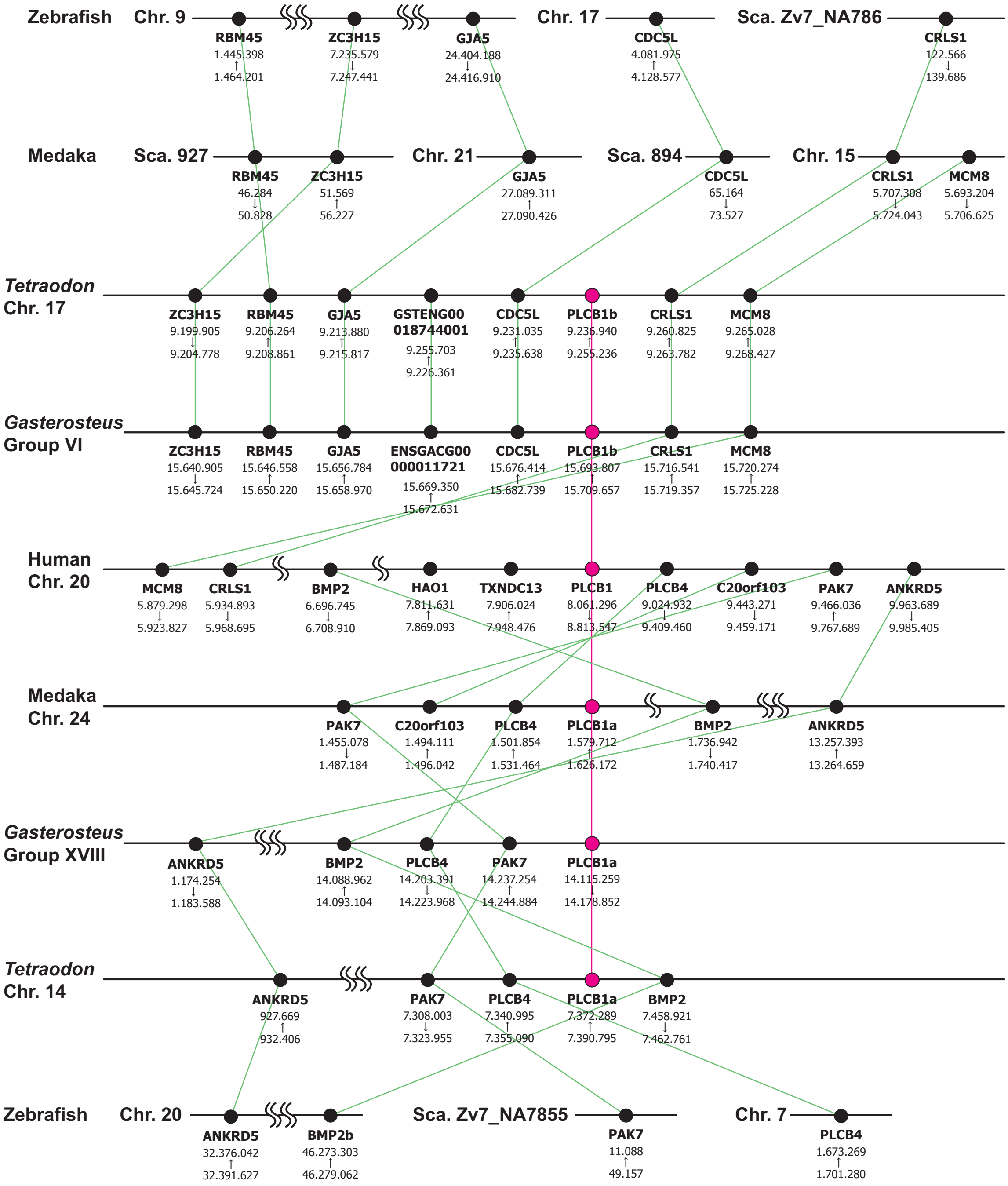

Fig. S64. Conserved synteny around the PLCB1 loci among human and the four teleosts (zebrafish, medaka, stickleback, and pufferfish). Lines show the orthologous/paralogous relationships among the genes.

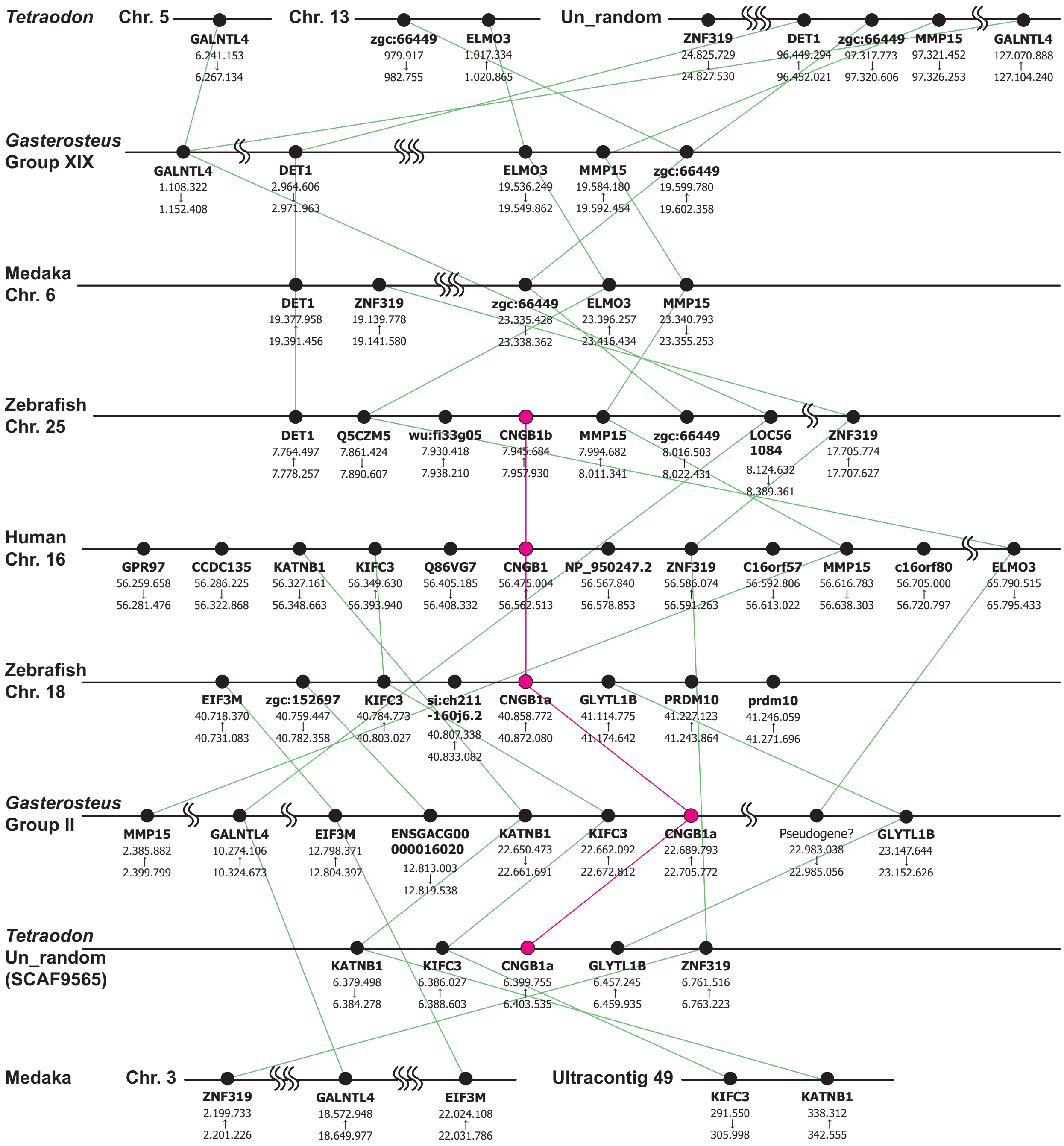

Fig. S65. Conserved synteny around the CNGB1 loci among human and the four teleosts (zebrafish, medaka, stickleback, and pufferfish). Lines show the orthologous/paralogous relationships among the genes.

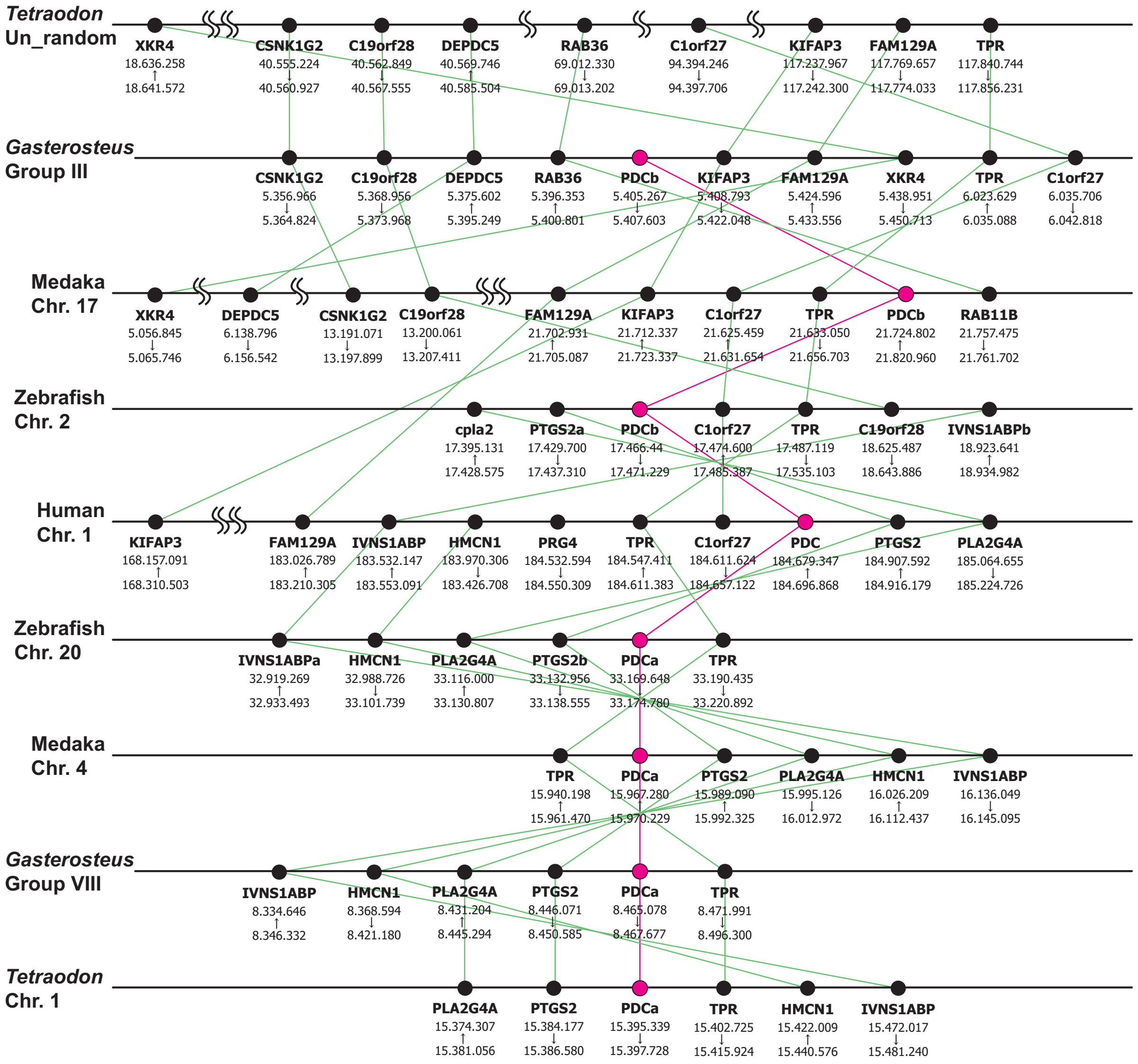

Fig. S66. Conserved synteny around the PDC loci among human and the four teleosts (zebrafish, medaka, stickleback, and pufferfish). Lines show the orthologous/paralogous relationships among the genes.

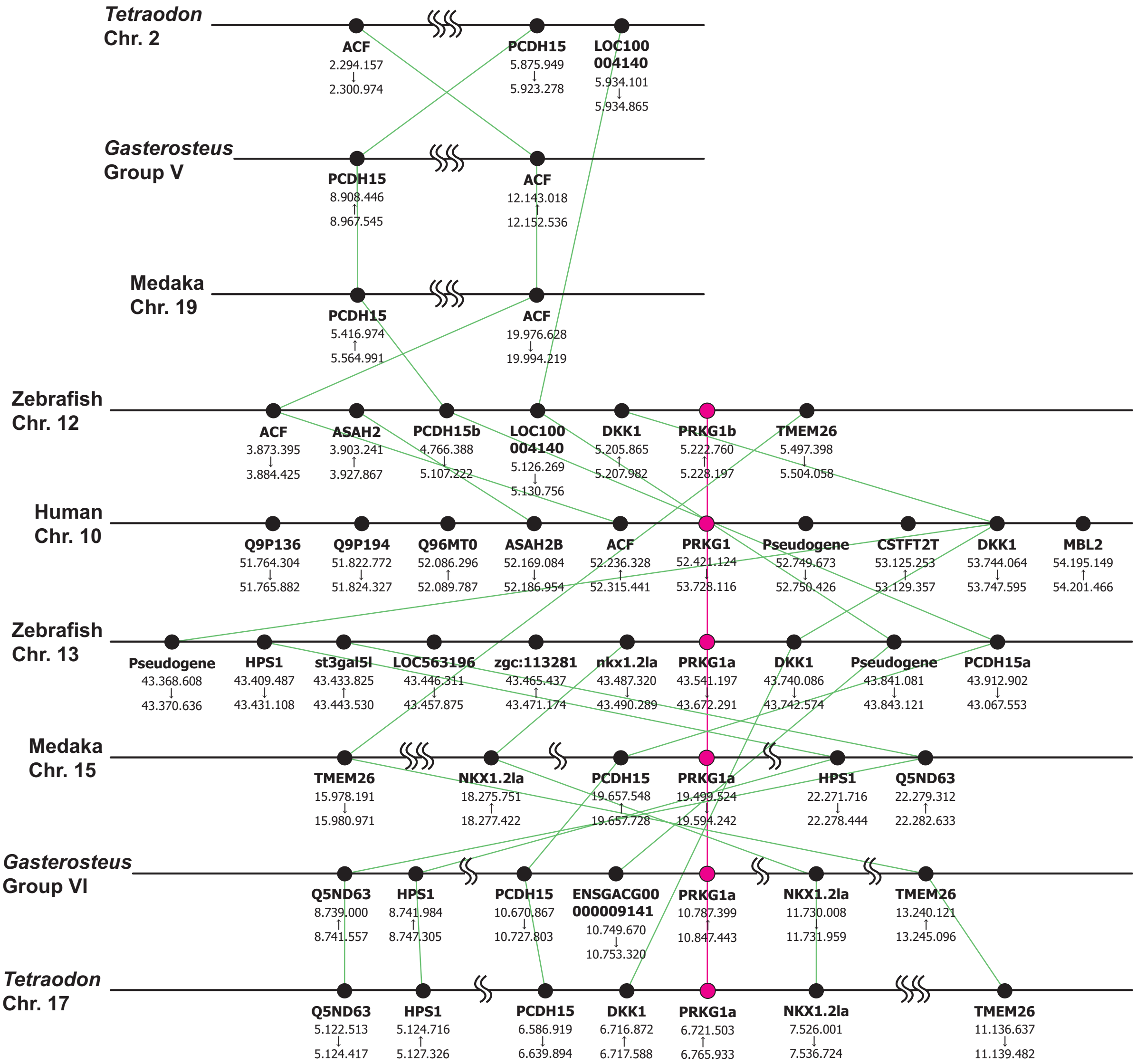

Fig. S67. Conserved synteny around the PRKG1 loci among human and the four teleosts (zebrafish, medaka, stickleback, and pufferfish). Lines show the orthologous/paralogous relationships among the genes.

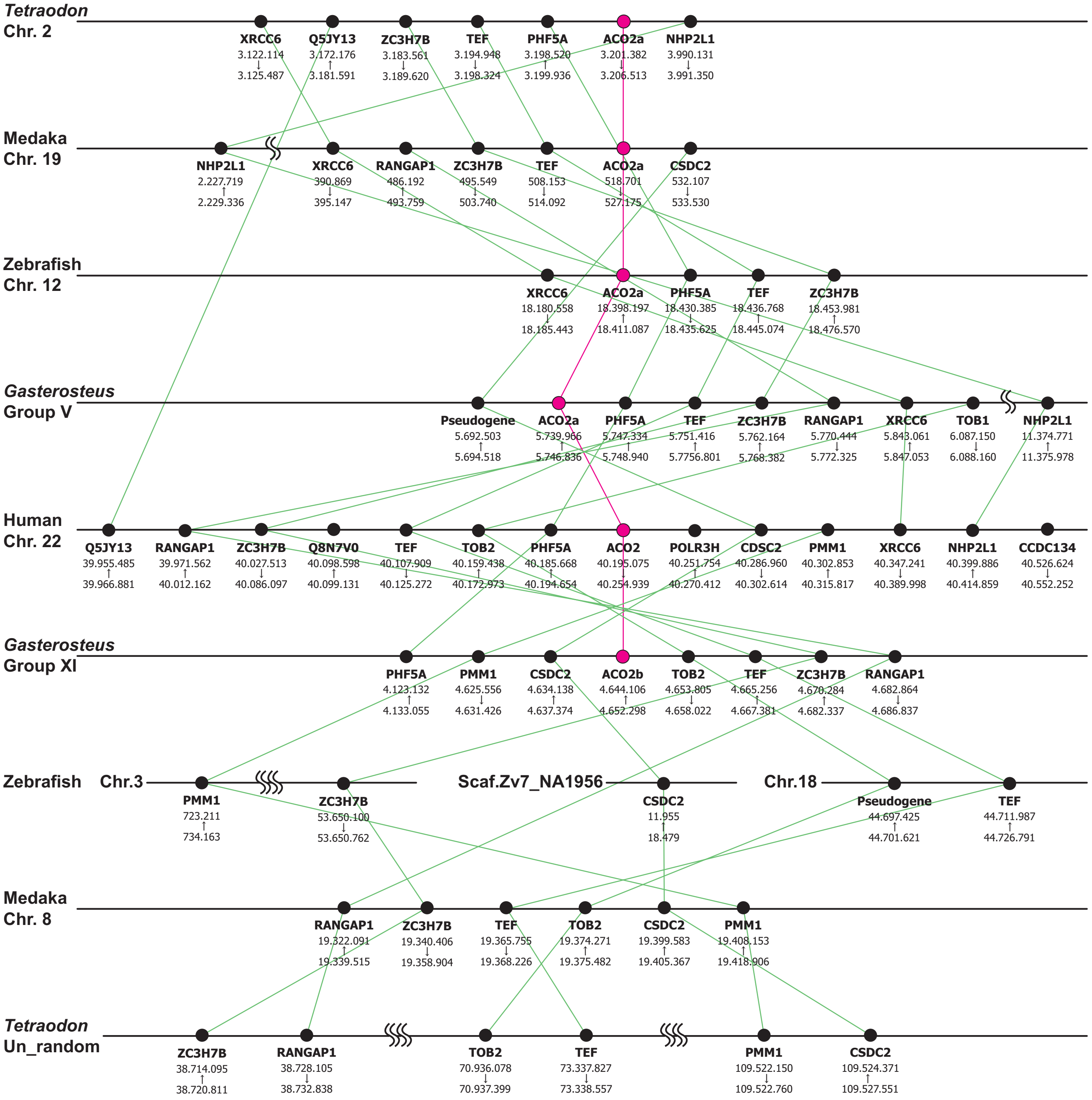

Fig. S68. Conserved synteny around the ACO2 loci among human and the four teleosts (zebrafish, medaka, stickleback, and pufferfish). Lines show the orthologous/paralogous relationships among the genes.
