# Supplementary material for: Temporal pattern of loss/persistence of duplicate genes involved in signal transduction and metabolic pathways after teleost-specific genome duplication
Source: BMC Evol Biol. 2009 Jun 5;9:127. doi: 10.1186/1471-2148-9-127 (PMC2702319; doi:10.1186/1471-2148-9-127)
Supplement: Additional file 3 — Supplementary appendix. This PDF file includes an appendix. [file 1471-2148-9-127-S3.pdf]

**Appendix (to be continued).**

| Gene name | Species | Ensembl ID | Length (aa) | Notes |
|---|---|---|---|---|
| **AMPAR (GRIA, glutamate receptor, ionotropic, AMPA)** | | | | |
| GRIA1_H | Human | ENSP00000285900 | 906 | |
| GRIA2_H | Human | ENSP00000264426 | 883 | |
| GRIA1a_T | *Tetraodon* | GSTENP00014672001 | 800 | |
| GRIA1b_T | *Tetraodon* | GSTENP00031632001 | 879 | |
| GRIA1_M | Medaka | ENSORLP00000009669 | 931 | |
| GRIA2_M | Medaka | ENSORLP00000025223 | 890 | |
| GRIA1_G | *Gasterosteus* | ENSGACP00000024435 | 963 | |
| GRIA2_G | *Gasterosteus* | ENSGACP00000023886 | 863 | |
| Gria2_Z | Zebrafish | ENSDARP00000071976 | 875 | |
| Gria1a_Z | Zebrafish | ENSDARG00000032714 | 917 | |
| Gria1b_Z | Zebrafish | ENSDARP00000043920 | 914 | |
| GRIA1_X | *Xenopus* | ENSXETP00000004107 | 900 | |
| GRIA1_C | Chicken | ENSGALP00000006483 | 902 | |
| GRIA2_C | Chicken | ENSGALP00000038049 | 883 | |
| **AC1/8 (ADCY1/8, adenylate cyclase 1 and 8 (brain) [EC:4.6.1.1])** | | | | |
| ADCY1_H | Human | ENSP00000373834 | 1119 | |
| ADCY8_H | Human | ENSP00000286355 | 1251 | |
| ADCY1a_T | *Tetraodon* | GSTENP00012275001 | 1085 | |
| ADCY1b_T | *Tetraodon* | GSTENP00004784001 | 788 | |
| ADCY_1_T | *Tetraodon* | GSTENP00012568001 | 1108 | |
| ADCY8_T | *Tetraodon* | GSTENP00015280001 | 971 | |
| ADCY_2_T | *Tetraodon* | GSTENP00030377001 | 1077 | |
| ADCY1a_M | Medaka | ENSORLP00000016126 | 720 | Program WISE2 was applied |
| ADCY1b_M | Medaka | ENSORLP00000021485 | 1030 | |
| ADCY_1_M | Medaka | ENSORLP00000001387 | 1091 | |
| ADCY8_M | Medaka | ENSORLP00000008051 | 902 | |
| ADCY_2_M | Medaka | ENSORLP00000017064 | 1061 | |
| ADCY1a_G | *Gasterosteus* | ENSGACP00000006434 | 1028 | |
| ADCY1b_G | *Gasterosteus* | ENSGACP00000017294 | 1094 | |
| ADCY_2_G | *Gasterosteus* | ENSGACP00000013316 | 1049 | |
| ADCY_1_Z | Zebrafish | ENSDARP00000041443 | 755 | Partial |
| ADCY_3_Z | Zebrafish | ENSDARP00000082160 | 933 | |
| ADCY_4_Z | Zebrafish | ENSDARP00000045039 | 723 | Program WISE2 was applied |
| ADCY1_X | *Xenopus* | ENSXETP00000021578 | 945 | |
| ADCY1_C | Chicken | ENSGALP00000020295 | 1090 | |
| ADCY8_C | Chicken | ENSGALP00000026243 | 1079 | |
| ADCY1_D | *Drosophila* | CG7978-PA | 1307 | |
| ADCYA_D | *Drosophila* | CG17176-PA | 1112 | |
| ADCYB_D | *Drosophila* | CG17174-PA | 1114 | |
| ADCYC_D | *Drosophila* | CG5983-PA | 1130 | |

**Appendix (continued).**

| Gene name | Species | Ensembl ID | Length (aa) | Notes |
|---|---|---|---|---|
| ADCYE_D | *Drosophila* | CG17178-PA | 1117 | |
| ADCY_Ci | *Ciona* | ENSCINP00000014863 | 1095 | |
| **NMDAR (GRIN, glutamate receptor, ionotropic, N-methyl D-aspartate)** | | | | |
| GRIN1_H | Human | ENSP00000360616 | 959 | |
| GRIN1b_T | *Tetraodon* | GSTENP00011452001 | 1223 | |
| GRIN1a_M | Medaka | ENSORLP00000017802 | 964 | |
| GRIN1b_M | Medaka | ENSORLP00000020601 | 967 | |
| GRIN1a_G | *Gasterosteus* | ENSGACP00000023949 | 941 | |
| GRIN1x_G | *Gasterosteus* | ENSGACP00000000304 | 196 | Partial |
| GRIN1a_Z | Zebrafish | ENSDARP00000041868 | 846 | Program WISE2 was applied |
| GRIN1b_Z | Zebrafish | ENSDARP00000038151 | 854 | |
| GRIN1_X | *Xenopus* | ENSXETP00000049601 | 904 | |
| GRIN1_C | Chicken | ENSGALP00000039569 | 901 | |
| Nmdar1_D | *Drosophila* | CG2902-PA | 997 | |
| GRIN2A_H | Human | ENSP00000332549 | 1464 | |
| GRIN2B_H | Human | ENSP00000279593 | 1484 | |
| GRIN2C_H | Human | ENSP00000293190 | 1233 | |
| GRIN2D_H | Human | ENSP00000263269 | 1336 | |
| GRIN2Aa_T | *Tetraodon* | GSTENP00015754001 | 1563 | |
| GRIN2Ab_T | *Tetraodon* | GSTENP00021857001 | 1403 | |
| GRIN2Ba_T | *Tetraodon* | GSTENP00010391001 | 1391 | |
| GRIN2Bb_T | *Tetraodon* | GSTENP00020497001 | 1548 | Program WISE2 was applied |
| GRIN2Ca_T | *Tetraodon* | GSTENP00021266001 | 968 | |
| GRIN2Cb_T | *Tetraodon* | GSTENP00027026001 | 960 | |
| GRIN2D_T | *Tetraodon* | GSTENP00033737001 | 1379 | |
| GRIN2Aa_M | Medaka | ENSORLP00000011524 | 1303 | |
| GRIN2Ab_M | Medaka | ENSORLP00000015929 | 1368 | |
| GRIN2Ba_M | Medaka | ENSORLP00000005277 | 1588 | |
| GRIN2Bb_M | Medaka | ENSORLP00000018670 | 1614 | |
| GRIN2Ca1_M | Medaka | ENSORLP00000001076 | 847 | |
| GRIN2Cb_M | Medaka | ENSORLP00000013759 | 842 | |
| GRIN2D_M | Medaka | ENSORLP00000014337 | 810 | |
| GRIN2Ca2_M | Medaka | UTOLAPRE05100105506 | 1114 | |
| GRIN2Aa_G | *Gasterosteus* | ENSGACP00000021959 | 1446 | |
| GRIN2Bb_G | *Gasterosteus* | ENSGACP00000009530 | 1629 | |
| GRIN2Ba_G | *Gasterosteus* | ENSGACP00000021088 | 1485 | |
| GRIN2Cb_G | *Gasterosteus* | ENSGACP00000004275 | 834 | |
| GRIN2Ca_G | *Gasterosteus* | ENSGACP00000019778 | 837 | |
| GRIN2D_G | *Gasterosteus* | ENSGACP00000014091 | 805 | |
| GRIN2Bb_Z | Zebrafish | ENSDARP00000087045 | 970 | |
| GRIN2Ba_Z | Zebrafish | ENSDARP00000047093 | 1604 | |
| GRIN2Ca_Z | Zebrafish | ENSDARP00000083814 | 839 | |

**Appendix (continued).**

| Gene name | Species | Ensembl ID | Length (aa) | Notes |
|---|---|---|---|---|
| GRIN2A_X | *Xenopus* | ENSXETP00000017540 | 1447 | |
| GRIN2C_X | *Xenopus* | ENSXETP00000014979 | 892 | |
| GRIN2D_X | *Xenopus* | ENSXETP00000023239 | 1271 | |
| GRIN2A_C | Chicken | ENSGALP00000011779 | 1437 | |
| GRIN2B_C | Chicken | ENSGALP00000019247 | 1370 | |
| **VDCC (CACNA1C, calcium channel, voltage-dependent, L type, alpha 1C subunit)** | | | | |
| CACNA1C_H | Human | ENSP00000329877 | 2208 | |
| CACNA1C_T | *Tetraodon* | GSTENP00014510001 | 1327 | Partial |
| CACNA1C_M | Medaka | ENSORLP00000017938 | 2017 | |
| CACNA1C_G | *Gasterosteus* | ENSGACP00000026026 | 2046 | |
| CACNA1C_Z | Zebrafish | ENSDARP00000019929 | 2196 | |
| CACNA1C_X | *Xenopus* | ENSXETP00000034191 | 855 | Program WISE2 was applied |
| CACNA1F_X | *Xenopus* | ENSXETP00000015655 | 1581 | |
| CACNA1C_C | Chicken | ENSGALP00000021218 | 1447 | Partial |
| Ca_alpha1D_D | *Drosophila* | CG4894-PC | 2552 | |
| CACNA1C_Ci | *Ciona* | ENSCINP00000015609 | 1645 | |
| **mGluR (GRM, glutamate receptor, metabotropic)** | | | | |
| GRM1_H | Human | ENSP00000282753 | 1194 | |
| GRM5_H | Human | ENSP00000306138 | 1212 | |
| GRM1a_T | *Tetraodon* | GSTENP00023057001 | 961 | |
| GRM1b_T | *Tetraodon* | GSTENP00035748001 | 1223 | |
| GRM5b_T | *Tetraodon* | GSTENP00018905001 | 1209 | |
| GRM1a_M | Medaka | ENSORLP00000017804 | 1207 | |
| GRM1b_M | Medaka | ENSORLP00000023504 | 855 | Partial |
| GRM5a_M | Medaka | ENSORLP00000013639 | 1229 | |
| GRM5b_M | Medaka | ENSORLP00000005680 | 1213 | |
| GRM1a_G | *Gasterosteus* | ENSGACP00000015408 | 1133 | |
| GRM1b_G | *Gasterosteus* | ENSGACP00000015341 | 1136 | |
| GRM5a_G | *Gasterosteus* | ENSGACP00000010565 | 1247 | |
| GRM5b_G | *Gasterosteus* | ENSGACP00000027224 | 1213 | |
| GRM1b_Z | Zebrafish | ENSDARP00000048545 | 1117 | |
| GRM1a_Z | Zebrafish | ENSDARP00000033374 | 1150 | |
| GRM5a_Z | Zebrafish | ENSDARP00000086408 | 966 | |
| GRM5b_Z | Zebrafish | ENSDARP00000042766 | 933 | |
| GRM1_X | *Xenopus* | ENSXETP00000017306 | 1245 | |
| GRM5_X | *Xenopus* | ENSXETP00000025701 | 1188 | |
| GRM1_C | Chicken | ENSGALP00000020069 | 1160 | |
| GRM5_C | Chicken | ENSGALP00000027813 | 1242 | |
| **Protein kinase A, cAMP-dependent, catalytic, alpha/beta/gamma [EC:2.7.11.11]** | | | | |
| PRKACA_H | Human | ENSP00000309591 | 351 | |

**Appendix (continued).**

| Gene name | Species | Ensembl ID | Length (aa) | Notes |
|---|---|---|---|---|
| PRKACB_H | Human | ENSP00000359723 | 351 | |
| PRKACG_H | Human | ENSP00000297734 | 351 | |
| PRKACA_T | *Tetraodon* | GSTENP00019760001 | 336 | |
| PRKACBa_T | *Tetraodon* | GSTENP00016205001 | 395 | |
| PRKACBb_T | *Tetraodon* | GSTENP00022860001 | 338 | |
| PRKACA_M | Medaka | ENSORLP00000008832 | 348 | |
| PRKACBa_M | Medaka | ENSORLP00000021647 | 351 | |
| PRKACBb_M | Medaka | ENSORLP00000007943 | 351 | |
| PRKACA_G | *Gasterosteus* | ENSGACP00000013822 | 351 | |
| PRKACBa_G | *Gasterosteus* | ENSGACP00000023579 | 351 | |
| PRKACBb_G | *Gasterosteus* | ENSGACP00000012693 | 351 | |
| PRKACA_Z | Zebrafish | ENSDARP00000002825 | 351 | |
| PRKACBa_Z | Zebrafish | ENSDARP00000023805 | 395 | |
| PRKACBb_Z | Zebrafish | ENSDARP00000076580 | 351 | |
| PRKACA_X | *Xenopus* | ENSXETP00000049427 | 311 | Partial |
| PRKACB_X | *Xenopus* | ENSXETP00000018929 | 337 | |
| PRKACB_C | Chicken | ENSGALP00000014351 | 398 | |
| PkaC1_D | *Drosophila* | CG4379-PA | 353 | |
| PKAC_Ci | *Ciona* | ENSCINP00000024366 | 357 | |
| **Protein kinase, X-linked/Y-linked [EC:2.7.11.11]** | | | | |
| PRKX_H | Human | ENSP00000262848 | 358 | |
| PRKY_H | Human | ENSP00000310643 | 277 | |
| PRKX_T | *Tetraodon* | GSTENP00027088001 | 344 | |
| PRKX_M | Medaka | ENSORLP00000025578 | 358 | |
| PRKX_G | *Gasterosteus* | ENSGACP00000020058 | 303 | |
| PRKX_X | *Xenopus* | ENSXETP00000010923 | 369 | |
| PRKX_C | Chicken | ENSGALP00000029643 | 302 | |
| PRKX_Z | Zebrafish | ENSDARP00000080286 | 306 | |
| PRKX_Ci | *Ciona savignyi* | ENSCSAVP00000017283 | 302 | |
| **IPP1 (I-1, PPP1R1A, protein phosphatase 1, regulatory (inhibitor) subunit 1A)** | | | | |
| IPP1_H | Human | ENSP00000257905 | 171 | |
| IPP1_T | *Tetraodon* | GSTENP00027617001 | 123 | |
| IPP1_M | Medaka | ENSORLP00000004271 | 164 | |
| IPP1_G | *Gasterosteus* | ENSGACP00000015367 | 97 | |
| IPP1_X | *Xenopus* | ENSXETP00000009351 | 184 | |
| IPP1C_C | Chicken | ENSGALP00000014453 | 115 | |
| IPP1a_Ci | *Ciona* | ENSCINP00000029740 | 448 | |
| IPP1b_Ci | *Ciona* | ENSCINP00000013063 | 315 | |
| **EPAC1 (RAPGEF3, Rap guanine nucleotide exchange factor (GEF) 3)** | | | | |
| EPAC1_H | Human | ENSP00000373863 | 881 | |

**Appendix (continued).**

| Gene name | Species | Ensembl ID | Length (aa) | Notes |
|---|---|---|---|---|
| EPAC1_T | *Tetraodon* | GSTENP00023559001 | 980 | |
| EPAC1_M | Medaka | ENSORLP00000019634 | 889 | |
| EPAC1_G | *Gasterosteus* | ENSGACP00000014548 | 874 | |
| EPAC1_Z | Zebrafish | ENSDARP00000079343 | 846 | |
| EPAC1_X | *Xenopus* | ENSXETP00000009345 | 856 | |
| EPAC1_C | Chicken | ENSGALP00000010246 | 766 | |
| EPAC1_D | *Drosophila* | CG3427-PA | 861 | |
| **Rap1 (RAP1A, RAP1A, member of RAS oncogene family)** | | | | |
| Rap1_H | Human | ENSP00000348786 | 184 | |
| Rap1_T | *Tetraodon* | GSTENP00013536001 | 225 | |
| Rap1_M | Medaka | ENSORLP00000007312 | 185 | |
| Rap1_G | *Gasterosteus* | ENSGACP00000008153 | 185 | |
| Rap1 _X | *Xenopus* | ENSXETP00000029726 | 185 | |
| Rap1_C | Chicken | ENSGALP00000002236 | 188 | |
| Rap1a_Z | Zebrafish | ENSDARP00000075571 | 185 | |
| Rap1b_Z | Zebrafish | ENSDARP00000050670 | 185 | |
| Rap1_D | *Drosophila* | CG1956-PA | 184 | |
| **PP1 (protein phosphatase 1)** | | | | |
| PPP1R12A_H | Human | ENSP00000261207 | 1028 | |
| PPP1R12A_T | *Tetraodon* | GSTENP00023775001 | 1007 | |
| PPP1R12A_M | Medaka | ENSORLP00000020423 | 1035 | |
| PPP1R12A_G | *Gasterosteus* | ENSGACP00000026440 | 1030 | |
| PPP1R12A_X | *Xenopus* | ENSXETP00000046417 | 979 | |
| PPP1R12A_C | Chicken | ENSGALP00000016789 | 1029 | |
| PPP1R12A_Z | Zebrafish | ENSDARP00000034186 | 1117 | |
| PPP1R_D | *Drosophila* | CG32156-PC | 1145 | |
| PPP1CA_H | Human | ENSP00000365936 | 330 | |
| PPP1CC_H | Human | ENSP00000335084 | 323 | |
| PPP1CAa_T | *Tetraodon* | GSTENP00027785001 | 326 | Program WISE2 was applied |
| PPP1CAb_T | *Tetraodon* | GSTENP00035052001 | 325 | |
| PPP1CC_T | *Tetraodon* | GSTENP00021602001 | 328 | |
| PPP1CAa_M | Medaka | ENSORLP00000002503 | 330 | |
| PPP1CAb_M | Medaka | ENSORLP00000007368 | 325 | |
| PPP1CC_M | Medaka | ENSORLP00000019963 | 323 | |
| PPP1CAb_G | Gasterosteus | ENSGACP00000009804 | 327 | |
| PPP1CAa_G | Gasterosteus | ENSGACP00000017909 | 332 | |
| PPP1CC_G | Gasterosteus | ENSGACP00000017831 | 323 | |
| PPP1CAa_Z | Zebrafish | ENSDARP00000002360 | 331 | |
| PPP1CC_X | *Xenopus* | ENSXETP00000052896 | 323 | |
| PPP1CC_C | Chicken | ENSGALP00000007265 | 323 | |
| PPP1_1_Ci | *Ciona* | ENSCINP00000001310 | 329 | Phylogenetically ambiguous |

**Appendix (continued).**

| Gene name | Species | Ensembl ID | Length (aa) | Notes |
|---|---|---|---|---|
| PPP1_2_Ci | *Ciona* | ENSCINP00000009413 | 343 | Phylogenetically ambiguous |
| PPP1CB_H | Human | ENSP00000351298 | 327 | |
| PPP1CBa_T | *Tetraodon* | GSTENP00031983001 | 299 | |
| PPP1CBa_M | Medaka | ENSORLP00000016746 | 327 | |
| PPP1CBb_M | Medaka | ENSORLP00000013924 | 328 | |
| PPP1CBb1_G | *Gasterosteus* | ENSGACP00000010813 | 331 | |
| PPP1CBa_G | *Gasterosteus* | ENSGACP00000013643 | 327 | |
| PPP1CBb2_G | *Gasterosteus* | ENSGACP00000026494 | 334 | |
| PPP1CBb_Z | Zebrafish | ENSDARP00000064810 | 310 | |
| PPP1CB_C | Chicken | ENSGALP00000031272 | 327 | |
| PPP1CB_D | *Drosophila* | CG2096-PB | 330 | Phylogenetically ambiguous |
| PPP1CB_Ci | *Ciona* | ENSCINP00000011235 | 327 | |
| **CaMK2 (calcium/calmodulin-dependent protein kinase (CaM kinase) II [EC:2.7.11.17])** | | | | |
| CAMK2A_H | Human | ENSP00000305090 | 489 | |
| CAMK2B_H | Human | ENSP00000258682 | 666 | |
| CAMK2D_H | Human | ENSP00000339740 | 499 | |
| CAMK2G_H | Human | ENSP00000307082 | 529 | |
| CAMK2A_T | *Tetraodon* | GSTENP00014562001 | 523 | |
| CAMK2D_T | *Tetraodon* | GSTENP00033048001 | 424 | |
| CAMK2Ga_T | *Tetraodon* | GSTENP00014312001 | 512 | |
| CAMK2Bb_T | *Tetraodon* | GSTENP00029080001 | 596 | |
| CAMK2Ba_T | *Tetraodon* | GSTENP00025360001 | 597 | |
| CAMK2A_M | Medaka | ENSORLP00000016108 | 513 | |
| CAMK2D_M | Medaka | ENSORLP00000006417 | 490 | |
| CAMK2x_M | Medaka | ENSORLP00000000306 | 642 | |
| CAMK2Ba_M | Medaka | ENSORLP00000001096 | 455 | Program WISE2 was applied |
| CAMK2Ga_M | Medaka | ENSORLP00000005224 | 559 | |
| CAMK2Gb_M | Medaka | ENSORLP00000013394 | 646 | |
| CAMK2A_G | *Gasterosteus* | ENSGACP00000027643 | 514 | |
| CAMK2D_G | *Gasterosteus* | ENSGACP00000021218 | 441 | |
| CAMK2Ga_G | *Gasterosteus* | ENSGACP00000014406 | 569 | |
| CAMK2Gb_G | *Gasterosteus* | ENSGACP00000003791 | 562 | |
| CAMK2Bb_Z | Zebrafish | ENSDARP00000039570 | 477 | |
| CAMK2Ba_Z | Zebrafish | ENSDARP00000044836 | 540 | |
| CAMK2D_Z | Zebrafish | ENSDARP00000044895 | 418 | |
| CAMK2Ga_Z | Zebrafish | ENSDARP00000073113 | 514 | |
| CAMK2Gb_Z | Zebrafish | ENSDARP00000044752 | 507 | |
| CAMK2D_X | *Xenopus* | ENSXETP00000033918 | 403 | |
| CAMK2B_X | *Xenopus* | ENSXETP00000041159 | 608 | |
| CAMK2G_X | *Xenopus* | ENSXETP00000014165 | 643 | |
| CAMK2A_C | Chicken | ENSGALP00000009006 | 489 | |
| CAMK2G_X | *Xenopus* | ENSXETP00000014165 | 643 | |

**Appendix (continued).**

| Gene name | Species | Ensembl ID | Length (aa) | Notes |
|---|---|---|---|---|
| CAMK2A_C | Chicken | ENSGALP00000009006 | 489 | |
| CAMK2D_C | Chicken | ENSGALP00000036906 | 483 | |
| CAMK2G_C | Chicken | ENSGALP00000008145 | 517 | |
| CaMKIIRD_D | *Drosophila* | CG18069-PD | 530 | |
| CAMK2_Ci | *Ciona* | ENSCINP00000004087 | 475 | Phylogenetically ambiguous |
| **CaN (calcium binding protein P22 and protein phosphatase 3)** | | | | |
| CHP_H | Human | ENSP00000335632 | 195 | |
| CHP_T | *Tetraodon* | GSTENP00035813001 | 232 | |
| CHP_M | Medaka | ENSORLP00000011997 | 195 | |
| CHP_G | *Gasterosteus* | ENSGACP00000016117 | 194 | |
| CHP_X | *Xenopus* | ENSXETP00000057366 | 193 | |
| CHP_C | Chicken | ENSGALP00000032005 | 195 | |
| CHP_Z | Zebrafish | ENSDARP00000069277 | 194 | |
| CG2185_D | *Drosophila* | CG2185-PA | 189 | |
| PPP3CA_H | Human | ENSP00000320580 | 521 | |
| PPP3CB_H | Human | ENSP00000353881 | 524 | |
| PPP3CC_H | Human | ENSP00000240139 | 512 | |
| PPP3CA_T | *Tetraodon* | GSTENP00019008001 | 571 | |
| PPP3CCa_T | *Tetraodon* | GSTENP00010354001 | 520 | |
| PPP3CA_M | Medaka | ENSORLP00000006587 | 519 | |
| PPP3CCb_M | Medaka | ENSORLP00000020174 | 505 | |
| PPP3CA_G | *Gasterosteus* | ENSGACP00000026029 | 524 | |
| PPP3CCb_G | *Gasterosteus* | ENSGACP00000006856 | 447 | |
| PPP3CCa_G | *Gasterosteus* | ENSGACP00000001534 | 454 | |
| PPP3CCb_Z | Zebrafish | ENSDARP00000015405 | 510 | |
| PPP3CCa_Z | Zebrafish | ENSDARP00000074861 | 500 | |
| PPP3CA_X | *Xenopus* | ENSXETP00000024402 | 499 | |
| PPP3CB_X | *Xenopus* | ENSXETP00000024405 | 530 | |
| PPP3CC_X | *Xenopus* | ENSXETP00000023087 | 489 | |
| PPP3CA_C | Chicken | ENSGALP00000020041 | 421 | |
| PPP3CB_C | Chicken | ENSGALP00000039533 | 522 | |
| PPP3Ca_D | *Drosophila* | CG1455-PA | 622 | |
| PPP3Cb_D | *Drosophila* | CG9819-PA | 584 | |
| PPP3Cc_D | *Drosophila* | CG9842-PA | 570 | |
| PPP3C_Ci | *Ciona* | ENSCINP00000021645 | 493 | |
| PPP3R1_H | Human | ENSP00000234310 | 160 | |
| PPP3R2_H | Human | ENSP00000342600 | 173 | |
| PPP3R_T | *Tetraodon* | GSTENP00007813001 | 169 | |
| PPP3R_M | Medaka | ENSORLP00000002910 | 169 | |
| PPP3R_Z | Zebrafish | ENSDARP00000022350 | 169 | |
| PPP3R_X | *Xenopus* | ENSXETP00000037793 | 169 | |

**Appendix (continued).**

| Gene name | Species | Ensembl ID | Length (aa) | Notes |
|---|---|---|---|---|
| PPP3R_X | *Xenopus* | ENSXETP00000037793 | 169 | |
| PPP3R_C | Chicken | ENSGALP00000014233 | 170 | |
| CanBRA_D | *Drosophila* | CG4209-PA | 170 | |
| CanB2_D | *Drosophila* | CG11217-PA | 170 | |
| **CAM (calmodulin)** | | | | |
| CALML6_H | Human | ENSP00000304643 | 181 | |
| CALM1_H | Human | ENSP00000349467 | 149 | |
| CALM2_H | Human | ENSP00000272298 | 149 | |
| CALM3_H | Human | ENSP00000315299 | 149 | |
| CALMx1_T | *Tetraodon* | GSTENP00018439001 | 149 | |
| CALMx2_T | *Tetraodon* | GSTENP00006151001 | 165 | |
| CALMx3_T | *Tetraodon* | GSTENP00031732001 | 174 | |
| CALMx4_T | *Tetraodon* | GSTENP00014816001 | 148 | |
| CALMx1_M | Medaka | ENSORLP00000008598 | 149 | |
| CALMx2_M | Medaka | ENSORLP00000001797 | 149 | |
| CALMx3_M | Medaka | ENSORLP00000022187 | 149 | |
| CALMx1_G | *Gasterosteus* | ENSGACP00000012669 | 151 | |
| CALMx2_G | *Gasterosteus* | ENSGACP00000003417 | 151 | |
| CALMx3_G | *Gasterosteus* | ENSGACP00000016676 | 154 | |
| CALMx4_G | *Gasterosteus* | ENSGACP00000006811 | 149 | |
| CALMx1_Z | Zebrafish | ENSDARP00000065391 | 148 | |
| CALMx2_Z | Zebrafish | ENSDARP00000035027 | 148 | |
| CALMx3_Z | Zebrafish | ENSDARP00000041779 | 148 | |
| CALMx4_Z | Zebrafish | ENSDARP00000053797 | 148 | |
| CALMx5_Z | Zebrafish | ENSDARP00000018820 | 148 | |
| CALM1a_X | *Xenopus* | ENSXETP00000034284 | 148 | |
| CALM2b_X | *Xenopus* | ENSXETP00000000663 | 148 | |
| CALM1_C | Chicken | ENSGALP00000036828 | 148 | |
| CALM3_C | Chicken | ENSGALP00000013706 | 149 | |
| CALM2_C | Chicken | ENSGALP00000037503 | 149 | |
| CALM6_C | Chicken | ENSGALP00000011046 | 143 | |
| CALMx1_D | *Drosophila* | CG17769-PA | 148 | |
| CALMx3_D | *Drosophila* | CG30378-PA | 148 | |
| CALMx2_D | *Drosophila* | CG8472-PA | 149 | |
| CALMx_Ci | *Ciona* | ENSCINP00000025381 | 161 | |
| **Ras (v-Ha-ras Harvey rat sarcoma viral oncogene homolog)** | | | | |
| HRAS_H | Human | ENSP00000309845 | 189 | |
| KRAS_H | Human | ENSP00000256078 | 189 | |
| NRAS_H | Human | ENSP00000358548 | 189 | |
| HRAS_T | *Tetraodon* | GSTENP00021499001 | 189 | |
| KRAS_T | *Tetraodon* | GSTENP00022812001 | 189 | |

**Appendix (continued).**

| Gene name | Species | Ensembl ID | Length (aa) | Notes |
|---|---|---|---|---|
| NRAS_T | *Tetraodon* | GSTENP00003165001 | 107 | Partial |
| HRAS_M | Medaka | ENSORLP00000023599 | 189 | |
| KRAS_M | Medaka | ENSORLP00000014318 | 189 | |
| xRAS_M | Medaka | ENSORLP00000014157 | 186 | |
| HRAS_Z | Zebrafish | ENSDARP00000016622 | 189 | |
| xRAS1_Z | Zebrafish | ENSDARP00000064608 | 187 | |
| NRAS_Z | *Zebrafish* | ENSDARP00000055741 | 188 | |
| xRAS2_Z | *Zebrafish* | ENSDARP00000021124 | 188 | |
| HRAS_G | *Gasterosteus* | ENSGACP00000014989 | 189 | |
| KRAS_G | Gasterosteus | ENSGACP00000015443 | 193 | |
| xRAS1_G | *Gasterosteus* | ENSGACP00000017051 | 187 | |
| xRAS2_G | *Gasterosteus* | ENSGACESTP00000015169 | 188 | |
| HRAS_X | *Xenopus* | ENSXETP00000013308 | 189 | |
| KRAS_X | *Xenopus* | ENSXETP00000007368 | 189 | |
| xRAS_X | *Xenopus* | ENSXETP00000013310 | 186 | |
| HRAS_C | Chicken | ENSGALP00000011140 | 189 | |
| xRAS_C | Chicken | ENSGALP00000022686 | 188 | |
| NRAS_C | Chicken | ENSGALP00000003258 | 193 | |
| Ras85D_D | *Drosophila* | CG9375-PA | 189 | |
| **Raf (v-raf murine sarcoma 3611 viral oncogene homolog [EC:2.7.11.1])** | | | | |
| ARAF_H | Human | ENSP00000366244 | 606 | |
| BRAF_H | Human | ENSP00000288602 | 766 | |
| ARAF_T | *Tetraodon* | GSTENP00020048001 | 671 | |
| BRAF_T | *Tetraodon* | GSTENP00013902001 | 771 | |
| ARAF_M | Medaka | ENSORLP00000020929 | 606 | |
| BRAF_M | Medaka | ENSORLP00000012350 | 768 | |
| ARAF_G | *Gasterosteus* | ENSGACP00000004834 | 609 | |
| BRAF_G | *Gasterosteus* | ENSGACP00000025456 | 810 | |
| ARAF_Z | Zebrafish | ENSDARP00000071167 | 608 | |
| RAFx_Z | Zebrafish | ENSDARP00000076954 | 688 | |
| BRAF_Z | Zebrafish | ENSDARP00000048390 | 817 | |
| ARAF_X | *Xenopus* | ENSXETP00000049610 | 327 | Partial |
| BRAF_X | *Xenopus* | ENSXETP00000010512 | 804 | |
| BRAF_C | Chicken | ENSGALP00000020950 | 760 | |
| phlRA_D | *Drosophila* | CG2845-PA | 739 | |
| Q4H2W3_Ci | *Ciona* | ENSCINP00000007716 | 451 | |
| RAF1_H | Human | ENSP00000251849 | 648 | |
| RAF1_T | *Tetraodon* | GSTENP00023725001 | 704 | |
| RAF1_M | Medaka | ENSORLP00000014365 | 662 | |
| RAF1_G | *Gasterosteus* | ENSGACP00000000078 | 629 | |
| RAF1_Z | *Zebrafish* | ENSDARP00000076954 | 688 | |
| RAF1_X | *Xenopus* | ENSXETP00000043489 | 636 | |

**Appendix (continued).**

| Gene name | Species | Ensembl ID | Length (aa) | Notes |
|---|---|---|---|---|
| RAF1_C | Chicken | ENSGALP00000007991 | 647 | |
| phlRA_D | *Drosophila* | CG2845-PA | 739 | |
| **MEK1/2 (MAP2K, mitogen-activated protein kinase kinase [EC:2.7.12.2])** | | | | |
| MAP2K1_H | Human | ENSP00000302486 | 393 | |
| MAP2K2_H | Human | ENSP00000262948 | 400 | |
| MAP2K1_T | *Tetraodon* | GSTENP00016020001 | 462 | |
| MAP2K2_T | *Tetraodon* | GSTENP00004843001 | 370 | |
| MAP2K1_M | Medaka | ENSORLP00000008701 | 394 | |
| MAP2K2_M | Medaka | ENSORLP00000006223 | 397 | |
| MAP2K1_G | *Gasterosteus* | ENSGACP00000006553 | 394 | |
| MAP2K2a_G | *Gasterosteus* | ENSGACP00000016808 | 399 | |
| MAP2K2b_G | *Gasterosteus* | ENSGACP00000020149 | 395 | |
| MAP2K1_Z | Zebrafish | ENSDARP00000008018 | 394 | |
| MAP2K2_Z | Zebrafish | ENSDARP00000014914 | 394 | Program WISE2 was applied |
| MAP2K1_X | *Xenopus* | ENSXETP00000034662 | 395 | |
| MAP2K1_C | Chicken | ENSGALP00000012448 | 395 | |
| MAP2K2_C | Chicken | ENSGALP00000001932 | 398 | |
| Dsor1RA_D | *Drosophila* | G15793-PA | 396 | |
| MAP2K_Ci | *Ciona* | ENSCINP00000008465 | 377 | |
| **ERK1/2 (MAPK, mitogen-activated protein kinase [EC:2.7.11.24])** | | | | |
| MAPK1_H | Human | ENSP00000215832 | 360 | |
| MAPK3_H | Human | ENSP00000263025 | 379 | |
| MAPK1_T | *Tetraodon* | GSTENP00028566001 | 366 | |
| MAPK3_T | *Tetraodon* | GSTENP00021811001 | 358 | |
| MAPK3_M | Medaka | ENSORLP00000015020 | 389 | |
| MAPK1_G | *Gasterosteus* | ENSGACP00000019049 | 369 | |
| MAPK3_G | *Gasterosteus* | ENSGACP00000017345 | 339 | |
| MAPK1_Z | Zebrafish | ENSDARP00000038550 | 369 | |
| MAPK3_Z | Zebrafish | ENSDARP00000055552 | 392 | |
| MAPK1_X | *Xenopus* | ENSXETP00000040234 | 361 | |
| MAPK1_C | Chicken | ENSGALP00000039208 | 321 | |
| **Rsk (RPS6KA, ribosomal protein S6 kinase, 90kDa, polypeptide [EC:2.7.11.1])** | | | | |
| RPS6KA6_H | Human | ENSP00000262752 | 745 | |
| RPS6KA1_H | Human | ENSP00000363277 | 744 | |
| RPS6KA2_H | Human | ENSP00000265678 | 733 | |
| RPS6KA3_H | Human | ENSP00000368884 | 740 | |
| RPS6KA6_T | *Tetraodon* | GSTENP00029331001 | 719 | |
| RPS6KA1_T | *Tetraodon* | GSTENP00032087001 | 732 | |
| RPS6KA2_T | *Tetraodon* | GSTENP00025809001 | 694 | |
| RPS6KA6_M | Medaka | ENSORLP00000010672 | 702 | |

**Appendix (continued).**

| Gene name | Species | Ensembl ID | Length (aa) | Notes |
|---|---|---|---|---|
| RPS6KA1_M | Medaka | ENSORLP00000018054 | 732 | |
| RPS6KA2_M | Medaka | ENSORLP00000012525 | 717 | |
| RPS6KA3_M | Medaka | ENSORLP00000003184 | 716 | |
| RPS6KA6_G | *Gasterosteus* | ENSGACP00000024513 | 745 | |
| RPS6KA1_G | *Gasterosteus* | ENSGACP00000012601 | 702 | |
| RPS6KA2_G | *Gasterosteus* | ENSGACP00000021164 | 739 | |
| RPS6KA3_G | *Gasterosteus* | ENSGACP00000016938 | 725 | |
| RPS6KA6_Z | Zebrafish | ENSDARP00000047992 | 732 | |
| RPS6KA1_Z | Zebrafish | ENSDARP00000050094 | 700 | |
| RPS6KA2_Z | Zebrafish | ENSDARP00000051816 | 708 | |
| RPS6KA3a_Z | Zebrafish | ENSDARP00000051545 | 732 | |
| RPS6KA3b_Z | Zebrafish | ENSDARP00000075154 | 699 | |
| RPS6KA6_X | *Xenopus* | ENSXETP00000031774 | 634 | |
| RPS6KA1_X | *Xenopus* | ENSXETP00000026921 | 697 | |
| RPS6KA2_X | *Xenopus* | ENSXETP00000022660 | 694 | |
| RPS6KA3_X | *Xenopus* | ENSXETP00000022653 | 482 | Partial |
| RPS6KA6_C | Chicken | ENSGALP00000011476 | 728 | |
| RPS6KA1_C | Chicken | ENSGALP00000000497 | 752 | |
| RPS6KA2_C | Chicken | ENSGALP00000018680 | 708 | |
| RPS6KA3_C | Chicken | ENSGALP00000026406 | 697 | |
| RPS6KA_Ci | *Ciona* | ENSCINP00000011581 | 747 | |
| **CREB (ATF4, activating transcription factor 4, tax-responsive enhancer element B67)** | | | | |
| ATF4_H | Human | ENSP00000336790 | 351 | |
| ATF4a_T | *Tetraodon* | GSTENP00021675001 | 315 | |
| ATF4b_M | Medaka | ENSORLP00000013351 | 375 | |
| ATF4a_M | Medaka | ENSORLP00000016837 | 352 | |
| ATF4a_G | *Gasterosteus* | ENSGACP00000010811 | 369 | |
| ATF4b_G | *Gasterosteus* | ENSGACP00000026215 | 392 | |
| ATF4b2_Z | Zebrafish | ENSDARP00000055608 | 396 | |
| ATF4b1_Z | Zebrafish | ENSDARP00000042969 | 339 | |
| ATF4_X | *Xenopus* | ENSXETP00000050104 | 343 | |
| ATF4_C | Chicken | ENSGALP00000037099 | 354 | |
| Q4H3V8_Ci | *Ciona* | ENSCINP00000022333 | 340 | |
| **CBP (CREB binding protein [EC:2.3.1.48])** | | | | |
| CREBBP_H | Human | ENSP00000262367 | 2442 (bp) | |
| EP300_H | Human | ENSP00000263253 | 2414 (bp) | |
| EP300b_T | *Tetraodon* | GSTENP00011900001 | 2730 (bp) | |
| EP300a_T | *Tetraodon* | GSTENP00024248001 | 2539 (bp) | |
| CREBBPa_T | *Tetraodon* | GSTENP00013510001 | 2473 (bp) | |
| CREBBPb_T | *Tetraodon* | GSTENP00026318001 | 982 (bp). | Partial; phylogenetically unsorted |
| CREBBPa_M | Medaka | ENSORLP00000010434 | 2297 (bp) | |
| CREBBPb_M | Medaka | UTOLAPRE05100117230 | 1864 (bp) | |

**Appendix (continued).**

| Gene name | Species | Ensembl ID | Length (aa) | Notes |
|---|---|---|---|---|
| EP300b_M | Medaka | ENSORLP00000017525 | 2420 (bp) | |
| CREBBPa_G | *Gasterosteus* | ENSGACP00000014994 | 2319 (bp) | |
| CREBBPb_G | *Gasterosteus* | ENSGACP00000024794 | 2397 (bp) | |
| EP300a_G | *Gasterosteus* | ENSGACP00000006405 | 2344 (bp) | |
| EP300b_G | *Gasterosteus* | ENSGACP00000010236 | 2411 (bp) | |
| CREBBPa_Z | Zebrafish | ENSDARP00000086311 | 1777 (bp) | Partial |
| EP300b2_Z | Zebrafish | ENSDARP00000083887 | 2401 (bp) | |
| CREBBPb_Z | Zebrafish | Chr22 31620000-31710000+ | 2326 (bp) | Program WISE2 was applied |
| EP300a_Z | Zebrafish | ENSDARP00000081212 | 1908 (bp) | |
| EP300b1_Z | Zebrafish | ENSDARP00000084542 | 2423 (bp) | |
| CREBBP_X | *Xenopus* | ENSXETP00000022709 | 2427 (bp) | |
| EP300_X | *Xenopus* | ENSXETP00000049701 | 2138 (bp) | Program WISE2 was applied |
| CREBBP_C | Chicken | ENSGALP00000012573 | 2432 (bp) | |
| EP300_C | Chicken | ENSGALP00000019537 | 2451 (bp) | |
| nejRB_D | *Drosophila* | CG15319-PB | 3276 (bp) | |
| CREBBP_Ci | *Ciona* | ENSCINP00000018331 | 1463 (bp) | |
| **CaMK4 (calcium/calmodulin-dependent protein kinase IV [EC:2.7.11.17])** | | | | |
| CaMK4_H | Human | ENSP00000282356 | 473 | |
| CaMK4_M | Medaka | ENSORLP00000017485 | 324 | |
| CaMK4_G | *Gasterosteus* | ENSGACP00000017983 | 360 | |
| CaMK4_X | *Xenopus* | ENSXETP00000015124 | 328 | |
| CaMK4_C | Chicken | ENSGALP00000024483 | 388 | |
| CaMK4_Z | Zebrafish | ENSDARP00000037747 | 364 | |
| CaMKIRA_D | *Drosophila* | CG1495-PA | 405 | |
| **PKC (protein kinase C [EC:2.7.11.13])** | | | | |
| PRKCA_H | Human | ENSP00000284384 | 672 | |
| PRKCB_H | Human | ENSP00000305355 | 673 | |
| PRKCG_H | Human | ENSP00000263431 | 697 | |
| PRKCAb_T | *Tetraodon* | GSTENP00026368001 | 656 | Program WISE2 was applied |
| PRKCAa_T | *Tetraodon* | GSTENP00033392001 | 740 | |
| PRKCBa_T | *Tetraodon* | GSTENP00021867001 | 633 | |
| PRKCBb_T | *Tetraodon* | GSTENP00009847001 | 825 | |
| PRKCBb_M | Medaka | ENSORLP00000010930 | 666 | |
| PRKCAa_M | Medaka | ENSORLP00000011078 | 578 | |
| PRKCAb_G | *Gasterosteus* | ENSGACP00000024481 | 667 | |
| PRKCAa_G | *Gasterosteus* | ENSGACP00000015453 | 670 | |
| PRKCBa_G | *Gasterosteus* | ENSGACP00000018940 | 671 | |
| PRKCBb_G | *Gasterosteus* | ENSGACP00000022148 | 664 | |
| PRKCBa_Z | Zebrafish | ENSDARP00000024929 | 668 | |
| PRKCB_X | *Xenopus* | ENSXETP00000037655 | 668 | |
| PRKCG_X | *Xenopus* | ENSXETP00000022125 | 659 | Program WISE2 was applied |

**Appendix (continued).**

| Gene name | Species | Ensembl ID | Length (aa) | Notes |
|---|---|---|---|---|
| PRKCA_C | Chicken | ENSGALP00000006278 | 674 | |
| PRKCB_C | Chicken | ENSGALP00000009662 | 608 | |
| Pkc53E_D | *Drosophila* | CG6622-PB | 679 | |
| PRKCx_Ci | *Ciona* | ENSCINP00000017438 | 779 | |
| **Gq (GNAQ, guanine nucleotide binding protein (G protein), q polypeptide)** | | | | |
| GNAQ_H | Human | ENSP00000286548 | 359 | |
| GNAQ_T | *Tetraodon* | GSTENP00029406001 | 502 | |
| GNAQ_M | Medaka | ENSORLP00000007128 | 358 | |
| GNAQ_G | *Gasterosteus* | ENSGACP00000010461 | 362 | |
| GNAQ_X | *Xenopus* | ENSXETP00000007815 | 359 | |
| GNAQ_C | Chicken | ENSGALP00000024446 | 359 | |
| GNAQ_Z | Zebrafish | ENSDARP00000020119 | 373 | |
| Q8WSR9_Ci | *Ciona* | ENSCINP00000022318 | 380 | |
| **PLCβ (phospholipase C, beta [EC:3.1.4.11])** | | | | |
| PLCB1_H | Human | ENST00000338037 | 7093 (bp) | |
| PLCB2_H | Human | ENST00000389812 | 3546 (bp) | |
| PLCB1b_T | *Tetraodon* | GSTENT00018741001 | 4386 (bp) | |
| PLCB1a_T | *Tetraodon* | GSTENT00020281001 | 2361 (bp) | |
| PLCB2_T | *Tetraodon* | GSTENT00032419001 | 2115 (bp) | |
| PLCB1a_M | Medaka | ENSORLT00000012252 | 3432 (bp) | |
| PLCB2_M | Medaka | UTOLAPRE05100101470 | 3159 (bp) | |
| PLCB1b_G | *Gasterosteus* | ENSGACT00000015673 | 3504 (bp) | |
| PLCB1a_G | *Gasterosteus* | ENSGACT00000016555 | 3657 (bp) | |
| PLCB2_G | *Gasterosteus* | ENSGACT00000008512 | 4020 (bp) | |
| PLCB1_X | *Xenopus* | ENSXETT00000012140 | 1410 (bp) | |
| PLCB2_X | *Xenopus* | ENSXETT00000029321 | 3537 (bp) | |
| PLCB1_C | Chicken | ENSGALT00000014420 | 3306 (bp) | |
| PLCB2_C | Chicken | ENSGALT00000007647 | 4007 (bp) | |
| PLCB3_H | Human | ENST00000279230 | 3705 (bp) | |
| PLCB3_T | *Tetraodon* | GSTENT00003549001 | 3714 (bp) | |
| PLCB3_M | Medaka | ENSORLT00000006894 | 3381 (bp) | |
| PLCB3_G | *Gasterosteus* | ENSGACT00000026168 | 3390 (bp) | |
| PLCB3_Z | Zebrafish | ENSDART00000073670 | 3423 (bp) | |
| PLCB3_X | *Xenopus* | ENSXETT00000015647 | 3618 (bp) | |
| Plc21C_D | *Drosophila* | CG4574-RD | 5749 (bp) | |
| PLCB4_H | Human | ENST00000278655 | 5488 (bp) | |
| PLCB4_T | *Tetraodon* | GSTENT00020282001 | 1593 (bp) | |
| PLCB4_M | Medaka | ENSORLT00000012106 | 3552 (bp) | |
| PLCB4_G | *Gasterosteus* | ENSGACT00000016591 | 3543 (bp) | |
| PLCB4_X | *Xenopus* | ENSXETT00000032998 | 1925 (bp) | |
| PLCB4_C | Chicken | ENSGALT00000014481 | 4095 (bp) | |

**Appendix (continued).**

| Gene name | Species | Ensembl ID | Length (aa) | Notes |
|---|---|---|---|---|
| norpA_D | *Drosophila* | CG3620-RA | 4225 (bp) | |
| PLCB_Ci | *Ciona savignyi* | ENSCSAVT00000004146 | 2640 (bp) | |
| **IP3R (ITPR3, inositol 1,4,5-triphosphate receptor, type 3)** | | | | |
| ITPR3_H | Human | ENSP00000363435 | 2671 | |
| ITPR1_H | Human | ENSP00000349029 | 2758 | |
| ITPR2_H | Human | ENSP00000370744 | 2703 | |
| ITPR3_T | *Tetraodon* | GSTENP00005900001 | 2848 | |
| ITPR1a_T | *Tetraodon* | GSTENP00014091001 | 2743 | |
| ITPR1b_T | *Tetraodon* | GSTENP00027211001 | 2336 | |
| ITPR2_T | *Tetraodon* | GSTENP00022806001 | 2747 | |
| ITPR3_M | Medaka | ENSORLP00000022924 | 2606 | |
| ITPR1a_M | Medaka | ENSORLP00000015946 | 2772 | |
| ITPR1b_M | Medaka | ENSORLP00000018255 | 2714 | |
| ITPR2a_M | Medaka | ENSORLP00000014246 | 2411 | |
| ITPR2b_M | Medaka | UTOLAPRE05100114864 | 2212 | |
| ITPR3_G | *Gasterosteus* | ENSGACP00000000328 | 2609 | |
| ITPR1b_G | *Gasterosteus* | ENSGACP00000000845 | 2765 | |
| ITPR1a_G | *Gasterosteus* | ENSGACP00000015790 | 2747 | |
| ITPR2_G | *Gasterosteus* | ENSGACP00000015473 | 2694 | |
| ITPR3a_Z | Zebrafish | ENSDARP00000082620 | 2688 | |
| ITPR3b_Z | Zebrafish | ENSDARP00000077685 | 2368 | |
| ITPRx_Z | Zebrafish | ENSDARP00000081374 | 1214 | Partial; phylogenetically ambiguous |
| ITPR2_Z | Zebrafish | ENSDARP00000061133 | 2624 | |
| ITPR1b2_Z | Zebrafish | ENSDARP00000033695 | 2345 | |
| ITPR1b1_Z | Zebrafish | ENSDARP00000080167 | 2715 | |
| ITPR1b3_Z | Zebrafish | ENSDARP00000080191 | 2685 | |
| ITPR3_1_X | *Xenopus* | ENSXETP00000033593 | 839 | Partial; phylogenetically ambiguous |
| ITPR3_2_X | *Xenopus* | ENSXETP00000040538 | 488 | Partial; phylogenetically ambiguous |
| ITPR1_X | *Xenopus* | ENSXETP00000011267 | 2692 | |
| ITPR2_X | *Xenopus* | ENSXETP00000035890 | 2105 | |
| ITPR3_C | Chicken | ENSGALP00000004972 | 2665 | |
| ITPR1_C | Chicken | ENSGALP00000013490 | 2751 | |
| ITPR2_C | Chicken | ENSGALP00000022744 | 2703 | |
| Itpr83A_D | *Drosophila* | CG1063-PB | 2837 | |
| ITPR_Ci | *Ciona* | ENSCINP00000015029 | 2054 | Program WISE2 was applied |
| **Gα (guanine nucleotide binding protein, alpha transducing)** | | | | |
| GNAT3_H | Human | OTTHUMT00000138730 | 342 | |
| GNAS_H | Human | ENSP00000265620 | 395 | |
| GNAT3_T | *Tetraodon* | GSTENP00028189001 | 336 | |
| GNAT1_T | *Tetraodon* | GSTENP00027493001 | 350 | |
| GNASb_T | *Tetraodon* | GSTENP00022571001 | 301 | |

**Appendix (continued).**

| Gene name | Species | Ensembl ID | Length (aa) | Notes |
|---|---|---|---|---|
| GNASa_T | *Tetraodon* | GSTENP00013542001 | 352 | |
| GNAT1_M | Medaka | ENSORLP00000023702 | 350 | |
| GNASa_M | Medaka | ENSORLP00000007194 | 398 | |
| GNAT1_G | *Gasterosteus* | ENSGACP00000016355 | 350 | |
| GNAT3_G | *Gasterosteus* | ENSGACP00000010234 | 352 | |
| GNASa_G | *Gasterosteus* | ENSGACP00000008323 | 394 | |
| GNASb_G | *Gasterosteus* | ENSGACP00000008186 | 380 | |
| GNAT1_Z | Zebrafish | ENSDARP00000064895 | 350 | |
| GNAT3_Z | Zebrafish | ENSDARP00000062362 | 354 | |
| GNASa_Z | Zebrafish | ENSORLP00000004563 | 379 | |
| GNAT1_X | *Xenopus* | ENSXETP00000015940 | 350 | |
| GNAT3_X | *Xenopus* | ENSXETP00000004299 | 354 | |
| GNAT3_C | Chicken | ENSGALP00000013713 | 373 | |
| GNAS_C | Chicken | ENSGALP00000012126 | 379 | |
| **GNB (guanine nucleotide binding protein, beta polypeptide 1 and 3)** | | | | |
| GNB1_H | Human | ENSP00000367872 | 340 | |
| GNB3_H | Human | ENSP00000229264 | 340 | |
| GNB1_T | *Tetraodon* | GSTENP00027506001 | 340 | |
| GNB3_T | *Tetraodon* | GSTENP00015314001 | 346 | |
| GNB1_M | Medaka | ENSORLP00000004503 | 340 | |
| GNB3a_M | Medaka | ENSORLP00000015882 | 340 | |
| GNB3b_M | Medaka | ENSORLP00000008043 | 349 | |
| GNB1_G | *Gasterosteus* | ENSGACP00000016084 | 340 | |
| GNB1x_G | *Gasterosteus* | ENSGACP00000007206 | 394 | |
| GNB3a_G | *Gasterosteus* | ENSGACP00000013068 | 370 | |
| GNB3b_G | *Gasterosteus* | ENSGACP00000012473 | 342 | |
| GNB1x_Z | Zebrafish | ENSDARP00000018443 | 321 | |
| GNB3a_Z | Zebrafish | ENSDARP00000017443 | 340 | |
| GNB3b_Z | Zebrafish | ENSDARP00000005843 | 338 | |
| GNB1_X | *Xenopus* | ENSXETP00000042401 | 341 | |
| GNB3_X | *Xenopus* | ENSXETP00000020865 | 340 | |
| GNB1_C | Chicken | ENSGALP00000039570 | 340 | |
| GNB3_C | Chicken | ENSGALP00000023365 | 340 | |
| **GNG (guanine nucleotide binding protein, gamma polypeptide 3 and 13)** | | | | |
| GNG3_H | Human | ENSP00000294117 | 75 | |
| GNG13_H | Human | ENSP00000248150 | 67 | |
| GNG3_T | *Tetraodon* | GSTENP00020393001 | 75 | |
| GNG13a_T | *Tetraodon* | GSTENP00008081001 | 67 | |
| GNG13x_T | *Tetraodon* | GSTENP00036646001 | 42 | Partial; phylogenetically unsorted |
| GNG13a_M | Medaka | ENSORLP00000010987 | 67 | |
| GNG13b_M | Medaka | ENSORLP00000020790 | 67 | |

**Appendix (continued).**

| Gene name | Species | Ensembl ID | Length (aa) | Notes |
|---|---|---|---|---|
| GNG3_G | *Gasterosteus* | ENSGACP00000026804 | 75 | |
| GNG13a_G | *Gasterosteus* | ENSGACP00000022136 | 67 | |
| GNGx1_G | *Gasterosteus* | ENSGACP00000005598 | 75 | |
| GNGx2_G | *Gasterosteus* | ENSGACP00000015903 | 75 | |
| GNG3_Z | Zebrafish | ENSDARP00000015972 | 75 | |
| GNG13a_Z | Zebrafish | ENSDARP00000036461 | 67 | |
| GNG2_Z | Zebrafish | ENSDARP00000073854 | 71 | |
| GNGx1_Z | Zebrafish | ENSDARP00000068714 | 75 | Phylogenetically unsorted |
| GNG13b_Z | Zebrafish | ENSDARP00000055268 | 67 | |
| GNG13_X | *Xenopus* | ENSXETP00000012710 | 67 | |
| GNG13_C | Chicken | ENSGALP00000008472 | 67 | |
| GNGx1_C | Chicken | ENSGALP00000020180 | 71 | |
| Ggamma1_D | *Drosophila* | CG8261-PA | 70 | |
| GNG13_D | *Drosophila* | CG3694-PA | 72 | |
| **AC4 (adenylate cyclase 4 [EC:4.6.1.1])** | | | | |
| ADCY4_H | Human | ENSP00000312126 | 1077 | |
| ADCY4b_T | *Tetraodon* | GSTENP00005702001 | 947 | Program WISE2 was applied |
| ADCY4a_G | *Gasterosteus* | ENSGACP00000022081 | 1110 | |
| ADCY4b_G | *Gasterosteus* | ENSGACP00000024950 | 1089 | |
| ADCY4a_M | Medaka | ENSORLP00000017520 | 1099 | |
| ADCY4b_M | Medaka | ENSORLP00000011324 | 1042 | |
| ADCY4a_Z | Zebrafish | ENSDARP00000078705 | 1083 | |
| ADCY4_X | *Xenopus* | ENSXETP00000036809 | 1075 | |
| ADCYx_C | Chicken | ENSGALP00000021289 | 1098 | |
| ADCY1_D | Drosophila | CG7978-PA | 1307 | |
| **AC6 (adenylate cyclase 6 [EC:4.6.1.1])** | | | | |
| ADCY6_H | Human | ENSP00000311405 | 1168 | |
| ADCY6x_T | *Tetraodon* | GSTENP00029196001 | 1772 | |
| ADCY6b_T | *Tetraodon* | GSTENP00028124001 | 1208 | |
| ADCY6x_G | *Gasterosteus* | ENSGACP00000001602 | 873 | Partial |
| ADCY6b_G | *Gasterosteus* | ENSGACP00000011337 | 1166 | |
| ADCY6a_G | *Gasterosteus* | ENSGACP00000003999 | 1148 | |
| ADCY6x_M | Medaka | ENSORLP00000012232 | 1193 | |
| ADCY6b_M | Medaka | ENSORLP00000008753 | 1170 | |
| ADCY6a_M | Medaka | ENSORLP00000015500 | 1192 | |
| ADCY6b_Z | Zebrafish | ENSDARP00000034426 | 747 | Partial |
| ADCY6a_Z | Zebrafish | ENSDARP00000005946 | 1175 | |
| ADCY6x_C | Chicken | ENSGALP00000019099 | 1212 | |
| ADCY1_D | *Drosophila* | CG7978-PA | 1307 | |

**Appendix (continued).**

| Gene name | Species | Ensembl ID | Length (aa) | Notes |
|---|---|---|---|---|
| **PDE1A (phosphodiesterase 1A, calmodulin-dependent [EC:3.1.4.17])** | | | | |
| PDE1A_H | Human | ENSP00000350858 | 535 | |
| PDE1A_T | *Tetraodon* | GSTENP00037899001 | 151 | Partial |
| PDE1A_M | Medaka | ENSORLP00000017334 | 543 | |
| PDE1A_G | *Gasterosteus* | ENSGACP00000010793 | 557 | |
| PDE1A_Z | Zebrafish | ENSDARP00000080640 | 476 | |
| PDE1A_C | Chicken | ENSGALP00000014423 | 509 | |
| Pde1cRA_D | Drosophila | CG14940-PA | 605 | |
| **CACN (calcium channel, voltage-dependent, P/Q type, alpha 1A subunit)** | | | | |
| CACNA1A_H | Human | ENSP00000353362 | 2505 | |
| CACNA1B_H | Human | ENSP00000360423 | 2339 | |
| CACNA1Ab_T | *Tetraodon* | GSTENP00021312001 | 1615 | |
| CACNA1Aa_T | *Tetraodon* | GSTENP00023728001 | 2040 | |
| CACNA1Ba_T | *Tetraodon* | GSTENT00009266001 | 1485 | |
| CACNA1Aa_M | Medaka | ENSORLP00000013836 | 2310 | |
| CACNA1Ba_M | Medaka | ENSORLP00000017325 | 1393 | |
| CACNA1Aa_G | *Gasterosteus* | ENSGACP00000016841 | 2000 | |
| CACNA1Ba_G | *Gasterosteus* | ENSGACP00000023871 | 2373 | |
| CACNA1Bb_G | *Gasterosteus* | ENSGACP00000004380 | 1557 | Program WISE2 was applied |
| CACNA1Aa_Z | Zebrafish | ENSDARP00000023232 | 1940 | |
| CACNA1Ab_Z | Zebrafish | ENSDARP00000005825 | 1751 | |
| CACNA1Bb_Z | Zebrafish | ENSDARP00000047718 | 1279 | |
| CACNA1B_C | Chicken | ENSGALP00000013765 | 2356 | |
| cacRE_D | *Drosophila* | CG1522-PE | 1851 | |
| CACNA1_Ci | *Ciona* | ENSCINP00000004981 | 1813 | |
| **TRPM5 (transient receptor potential cation channel, subfamily M, member 5)** | | | | |
| TRPM5_H | Human | ENSP00000155858 | 1165 | |
| TRPM5_T | *Tetraodon* | GSTENP00018196001 | 1082 | Program WISE2 was applied |
| TRPM5_M | Medaka | ENSORLP00000001370 | 1132 | |
| TRPM5_G | *Gasterosteus* | ENSGACP00000022654 | 1113 | |
| TRPM5_Z | Zebrafish | ENSDARP00000077915 | 1139 | |
| TRPM5_C | Chicken | ENSGALP00000010522 | 1120 | |
| TRPM_D | *Drosophila* | CG30078-PA | 1131 | |
| TRPM_CiS | *Ciona savignyi* | ENSCSAVP00000015105 | 941 | |
| **KCN (KCNB1, potassium voltage-gated channel, Shab-related subfamily, member 1)** | | | | |
| KCNB1_H | Human | ENSP00000360806 | 858 | |
| KCNB1_T | *Tetraodon* | GSTENP00027468001 | 792 | |
| KCNx_T | *Tetraodon* | GSTENP00033644001 | 528 | |
| KCNB1_M | Medaka | ENSORLP00000000766 | 749 | |
| KCNB1_G | *Gasterosteus* | ENSGACP00000001736 | 826 | |

**Appendix (continued).**

| Gene name | Species | Ensembl ID | Length (aa) | Notes |
|---|---|---|---|---|
| KCNx_G | *Gasterosteus* | ENSGACP00000019593 | 533 | Program WISE2 was applied |
| KCNB1_X | *Xenopus* | ENSXETP00000034901 | 875 | |
| KCNx_X | *Xenopus* | ENSXETP00000025847 | 709 | |
| KCNB1_C | Chicken | ENSGALP00000033591 | 855 | |
| KCNB1_Z | Zebrafish | ENSDARP00000078796 | 831 | |
| ShabRB_D | *Drosophila* | CG1066-PB | 1015 | |
| **AC3 (ADCY3, adenylate cyclase 3 [EC:4.6.1.1])** | | | | |
| ADCY3_H | Human | ENSP00000260600 | 1144 | |
| ADCY3b1_T | *Tetraodon* | GSTENP00012767001 | 928 | |
| ADCY3b2_T | *Tetraodon* | GSTENP00012928001 | 982 | |
| ADCY3a_T | *Tetraodon* | GSTENP00021989001 | 1292 | |
| ADCY3b_M | Medaka | ENSORLP00000021102 | 1135 | |
| ADCY3a_M | Medaka | ENSORLP00000003093 | 923 | |
| ADCY3a_G | *Gasterosteus* | ENSGACP00000018535 | 1153 | |
| ADCY3b_G | *Gasterosteus* | ENSGACP00000017710 | 1132 | |
| ADCY3b_Z | Zebrafish | ENSDARP00000012983 | 1052 | |
| ADCY3_X | *Xenopus* | ENSXETP00000045792 | 1079 | |
| ADCY3_C | Chicken | ENSGALP00000026753 | 1133 | |
| rutRA_D | *Drosophila* | CG9533-PA | 2248 | |
| **CNGB1 (cyclic nucleotide gated channel beta 1)** | | | | |
| CNGB1_H | Human | ENSP00000251102 | 1251 | |
| CNGB1_T | *Tetraodon* | GSTENP00007198001 | 743 | |
| CNGB1_G | *Gasterosteus* | ENSGACP00000023240 | 594 | Program WISE2 was applied |
| CNGB1a_Z | Zebrafish | ENSDARP00000089356 | 613 | |
| CNGB1b_Z | Zebrafish | ENSDARP00000061711 | 614 | |
| CNGB1a_X | *Xenopus* | ENSXETP00000030119 | 1067 | |
| CNGB1b_X | *Xenopus* | ENSXETP00000030000 | 689 | |
| CNGB1_C | Chicken | ENSGALP00000001491 | 713 | |
| **CNGA (cyclic nucleotide gated channel alpha)** | | | | |
| CNGA3_H | Human | ENSP00000272602 | 694 | |
| CNGA4_H | Human | ENSP00000369268 | 575 | |
| CNGA3a1_T | *Tetraodon* | GSTENP00025775001 | 563 | |
| CNGA3b_T | *Tetraodon* | GSTENP00027057001 | 468 | |
| CNGA3a2_T | *Tetraodon* | GSTENP00032742001 | 563 | |
| CNGA4_T | *Tetraodon* | GSTENP00010054001 | 511 | |
| CNGA3a_M | Medaka | ENSORLP00000019723 | 613 | |
| CNGA3b_M | Medaka | ENSORLP00000025597 | 515 | |
| CNGA4_M | Medaka | ENSORLP00000012922 | 575 | |
| CNGA3a_G | *Gasterosteus* | ENSGACP00000018154 | 720 | |
| CNGA4_G | *Gasterosteus* | ENSGACP00000002781 | 611 | |

**Appendix (continued).**

| Gene name | Species | Ensembl ID | Length (aa) | Notes |
|---|---|---|---|---|
| CNGA3b_Z | Zebrafish | ENSDARP00000022566 | 622 | |
| CNGA3a1_Z | Zebrafish | ENSDARP00000069528 | 735 | |
| CNGA3a2_Z | Zebrafish | ENSDARP00000069652 | 666 | |
| CNGA3_X | *Xenopus* | ENSXETP00000052592 | 737 | |
| CNGA4_X | *Xenopus* | ENSXETP00000023078 | 518 | |
| CNGA3_C | Chicken | ENSGALP00000026992 | 731 | |
| CNGA4_C | Chicken | ENSGALP00000035597 | 530 | |
| Cng_D | *Drosophila* | CG7779-PA | 665 | |
| CNG_Ci | *Ciona* | ENSCINP00000010344 | 541 | |
| **ARRB2 (arrestin, beta 2)** | | | | |
| ARRB2_H | Human | ENSP00000269260 | 409 | |
| ARR1_T | *Tetraodon* | GSTENP00021161001 | 575 | |
| ARRB2a_T | *Tetraodon* | GSTENP00006671001 | 303 | Program WISE2 was applied |
| ARRB2b_T | *Tetraodon* | GSTENP00014682001 | 386 | |
| ARRB1_M | Medaka | ENSORLP00000025743 | 409 | |
| ARRB2b_M | Medaka | ENSORLP00000016469 | 418 | |
| ARRB1_G | *Gasterosteus* | ENSGACP00000006046 | 415 | |
| ARRB2a_G | *Gasterosteus* | ENSGACP00000000965 | 407 | |
| ARRB2b_G | *Gasterosteus* | ENSGACP00000002368 | 403 | |
| ARR1_Z | Zebrafish | ENSDARESTP00000025319 | 382 | Program WISE2 was applied |
| ARRB2b2_Z | Zebrafish | ENSDARP00000024694 | 401 | |
| ARRB2b1_Z | Zebrafish | ENSDARP00000057290 | 406 | |
| ARRB1_X | *Xenopus* | ENSXETP00000021181 | 404 | |
| ARR_C | Chicken | ENSGALP00000006752 | 359 | |
| krzRA_D | *Drosophila* | CG1487-PA | 470 | |
| Q8MYB1_Ci | Ciona | ENSCINP00000025095 | 418 | |
| **GRK (ADRBK2, adrenergic, beta, receptor kinase 2 [EC:2.7.11.15])** | | | | |
| ADRBK2_H | Human | ENSP00000317578 | 688 | |
| ADRBK2_T | *Tetraodon* | GSTENP00025383001 | 697 | |
| ADRBK2_M | Medaka | ENSORLP00000001399 | 600 | |
| ADRBK2_Z | Zebrafish | ENSDARP00000007164 | 370 | Program WISE2 was applied |
| ADRBK2_C | Chicken | ENSGALP00000008839 | 625 | |
| ADRBK2x_C | Chicken | ENSGALP00000028987 | 465 | |
| Gprk1_D | *Drosophila* | CG40129-PA | 513 | |
| ADRBK_Ci | *Ciona* | ENSCINP00000002847 | 511 | |
| **Phd (PDC, phosducin)** | | | | |
| PDC_H | Human | ENSP00000342033 | 246 | |
| PDCa_T | *Tetraodon* | GSTENP00033630001 | 235 | |
| PDCa_M | Medaka | ENSORLP00000011833 | 242 | |
| PDCb_M | Medaka | ENSORLP00000017422 | 259 | |

**Appendix (continued).**

| Gene name | Species | Ensembl ID | Length (aa) | Notes |
|---|---|---|---|---|
| PDCa_G | *Gasterosteus* | ENSGACP00000009882 | 254 | |
| PDCb_G | *Gasterosteus* | ENSGACP00000019408 | 240 | |
| PDCb1_Z | Zebrafish | ENSDARP00000017670 | 245 | |
| PDCb2_Z | Zebrafish | ENSDARP00000010853 | 261 | |
| PDC_X | *Xenopus* | ENSXETP00000037837 | 229 | |
| PDC_C | Chicken | ENSGALP00000008117 | 249 | |
| **GNAL (Golf, G protein, alpha activating activity polypeptide, olfactory type)** | | | | |
| GNAL_H | Human | ENSP00000269162 | 381 | |
| GNAL1_T | *Tetraodon* | GSTENP00007449001 | 357 | |
| GNAL2_T | *Tetraodon* | GSTENP00027664001 | 343 | |
| GNAL1_M | Medaka | ENSORLP00000024591 | 391 | |
| GNAL2_M | Medaka | ENSORLP00000004678 | 396 | |
| GNAL1_G | *Gasterosteus* | ENSGACP00000001495 | 377 | |
| GNAL2_G | *Gasterosteus* | ENSGACP00000021927 | 379 | |
| GNAL1_Z | Zebrafish | ENSDARP00000066781 | 379 | |
| GNAL_X | *Xenopus* | ENSXETP00000042648 | 379 | |
| GNAL_C | Chicken | ENSGALP00000022345 | 379 | |
| Gsalpha60A_D | *Drosophila* | CG2835-PB | 385 | |
| GNAL_Ci | *Ciona* | ENSCINP00000011969 | 381 | |
| **PKG (protein kinase, cGMP-dependent, type I and type II [EC:2.7.11.12])** | | | | |
| PRKG1_H | Human | ENSP00000327642 | 671 | |
| PRKG1_C | Chicken | ENSGALP00000006027 | 685 | |
| PRKG1a_T | *Tetraodon* | GSTENP00016387001 | 726 | |
| CGK1_T | *Tetraodon* | GSTENP00005488001 | 831 | |
| PRKG1a_M | Medaka | ENSORLP00000007952 | 684 | |
| PRKG1a_G | *Gasterosteus* | ENSGACP00000012095 | 684 | |
| CGK1_G | *Gasterosteus* | ENSGACP00000013540 | 586 | |
| PRKG1a_Z | Zebrafish | ENSDARP00000024589 | 667 | |
| PRKG1b_Z | Zebrafish | ENSDARP00000043235 | 329 | Program WISE2 was applied |
| Pkg21D_D | *Drosophila* | CG3324-PA | 768 | |
| CGKI_Ci | *Ciona* | ENSCINP00000025733 | 580 | |
| PRKG2_H | *Human* | ENSP00000264399 | 762 | |
| PRKG2_C | *Chicken* | ENSGALP00000017721 | 755 | |
| PRKG2_X | *Xenopus* | ENSXETP00000046551 | 788 | |
| CGK2_X | *Xenopus* | ENSXETP00000014175 | 608 | |
| PRKG2_T | *Tetraodon* | GSTENP00006641001 | 293 | Partial; phylogenetically unsorted |
| PRKG2_M | *Medaka* | ENSORLP00000022254 | 768 | |
| CGK2_M | *Medaka* | ENSORLP00000001301 | 766 | |
| PRKG2_G | *Gasterosteus* | ENSGACP00000002402 | 457 | Partial |
| PRKG2_Z | *Zebrafish* | ENSDARP00000071397 | 769 | |
| CGK2_Z | *Zebrafish* | ENSDARP00000085704 | 763 | |

**Appendix (continued).**

| Gene name | Species | Ensembl ID | Length (aa) | Notes |
|---|---|---|---|---|
| PRKG_Ci | *Ciona* | ENSCINP00000007393 | 725 | |
| **PDE1C (phosphodiesterase 1C, calmodulin-dependent 70kDa [EC:3.1.4.17])** | | | | |
| PDE1C_H | Human | ENSP00000284920 | 769 | |
| PDE1Ca_T | *Tetraodon* | GSTENP00034501001 | 695 | |
| PDE1Cb_T | *Tetraodon* | GSTENP00009446001 | 601 | |
| PDE1Ca_M | Medaka | ENSORLP00000022278 | 433 | |
| PDE1A_M | Medaka | ENSORLP00000017334 | 543 | |
| PDE1Ca_G | *Gasterosteus* | ENSGACP00000022683 | 554 | |
| PDE1Cb_1_G | *Gasterosteus* | ENSGACP00000000002 | 525 | |
| PDE1Cb_2_G | *Gasterosteus* | ENSGACP00000001141 | 508 | |
| PDE1Cb_3_G | *Gasterosteus* | ENSGACP00000001232 | 463 | |
| PDE1Cb_4_G | *Gasterosteus* | ENSGACP00000001336 | 246 | Partial; phylogenetically unsorted |
| PDE1Cb_5_G | *Gasterosteus* | ENSGACP00000001443 | 420 | |
| PDE1Cb_6_G | *Gasterosteus* | ENSGACP00000001564 | 564 | |
| PDE1Cb_7_G | *Gasterosteus* | ENSGACP00000014854 | 472 | |
| PDE1Cb_8_G | *Gasterosteus* | ENSGACP00000020708 | 498 | |
| PDE1A_Z | Zebrafish | ENSDARP00000080640 | 476 | |
| PDE1Ca_Z | Zebrafish | ENSDARP00000067654 | 395 | Partial |
| PDE1C_X | *Xenopus* | ENSXETP00000050616 | 662 | |
| PDE1C_C | Chicken | ENSGALP00000019935 | 601 | |
| PDE1A_C | Chicken | ENSGALP00000014423 | 509 | |
| Pde1c_D | *Drosophila* | CG14940-PA | 605 | |
| **GCAP (GUCA, guanylate cyclase activator)** | | | | |
| GUCA1A_H | Human | ENSP00000053469 | 201 | |
| GUCA1B_H | Human | ENSP00000230361 | 200 | |
| GUCA1C_H | Human | ENSP00000261047 | 209 | |
| GUCA1A_T | *Tetraodon* | GSTENP00027319001 | 190 | |
| GUCA1B_T | *Tetraodon* | GSTENP00023626001 | 177 | |
| GUCA1x_M | Medaka | ENSORLP00000019893 | 191 | |
| GUCA1B_M | Medaka | ENSORLP00000016751 | 197 | |
| GUCA1B_G | *Gasterosteus* | ENSGACP00000004424 | 197 | |
| GUCA1A_X | *Xenopus* | ENSXETP00000003235 | 203 | |
| GUCA1B_X | *Xenopus* | ENSXETP00000003231 | 159 | |
| GUCA1C_X | *Xenopus* | ENSXETP00000020214 | 187 | |
| GUCA1x_X | *Xenopus* | ENSXETP00000008239 | 191 | |
| GUCA1A_C | Chicken | ENSGALP00000028327 | 199 | |
| GUCA1B_C | Chicken | ENSGALP00000002158 | 198 | |
| GUCA1C_C | Chicken | ENSGALP00000029794 | 187 | |
| GUCA1A_Z | Zebrafish | ENSDARP00000063553 | 198 | |
| GUCA1B_Z | Zebrafish | ENSDARP00000019977 | 197 | |
| CG7646 _D | *Drosophila* | CG7646-PA | 186 | |

**Appendix (continued).**

| Gene name | Species | Ensembl ID | Length (aa) | Notes |
|---|---|---|---|---|
| GUCA1_Ci | *Ciona* | ENSCINP00000004308 | 155 | |
| GUCA2_Ci | *Ciona* | ENSCINP00000027731 | 194 | |
| **pGC (Gucy, guanylate cyclase)** | | | | |
| Gucy2d_H | Human | ENSP00000369550 | 1103 | |
| Gucy2f_H | Human | ENSP00000218006 | 1108 | |
| Gucy2f_T | *Tetraodon* | GSTENP00005625001 | 1071 | |
| GUCY2d1_T | *Tetraodon* | GSTENP00022134001 | 1037 | |
| GUCY2d2_T | *Tetraodon* | GSTENP00004053001 | 1154 | |
| Gucy2f_M | Medaka | ENSORLP00000011929 | 1133 | |
| GUCY2d1_M | Medaka | ENSORLP00000009861 | 1126 | |
| Gucy2f_G | *Gasterosteus* | ENSGACP00000024728 | 1109 | |
| GUCY2d1_G | *Gasterosteus* | ENSGACP00000026963 | 1148 | |
| GUCY2d2_G | *Gasterosteus* | ENSGACP00000001536 | 1057 | |
| GUCY2d1_Z | Zebrafish | ENSDARP00000030649 | 1055 | Program WISE2 was applied |
| Gucy2f_Z | Zebrafish | ENSDARP00000012115 | 1045 | |
| Gucy2f_X | *Xenopus* | ENSXETP00000003311 | 999 | |
| GUCY2d_X | *Xenopus* | ENSXETP00000019910 | 956 | |
| Gucy2f_C | Chicken | ENSGALP00000001168 | 1307 | |
| CG31183_D | *Drosophila* | CG31183-PA | 1417 | |
| **CLCA (chloride channel, calcium activated)** | | | | |
| CLCA1_H | Human | ENSP00000234701 | 914 | |
| CLCA2_H | Human | ENSP00000359596 | 943 | |
| CLCA4_H | Human | ENSP00000263723 | 919 | |
| CLCA | *Tetraodon* | --- | --- | No significant BLAST hits |
| CLCA | Medaka | --- | --- | No significant BLAST hits |
| CLCA | *Gasterosteus* | --- | --- | No significant BLAST hits |
| CLCA1a_Z | *Zebrafish* | ENSDARP00000023466 | 830 | Phylogenetically ambiguous |
| CLCA1b_Z | *Zebrafish* | ENSDARP00000051965 | 925 | Phylogenetically ambiguous |
| CLCA1a_X | *Xenopus* | ENSXETP00000001081 | 855 | |
| CLCA1b_X | *Xenopus* | ENSXETP00000001090 | 886 | |
| CLCA1c_X | *Xenopus* | ENSXETP00000001100 | 933 | |
| CLCA1d_X | *Xenopus* | ENSXETP00000006708 | 872 | |
| CLCA_C | Chicken | ENSGALP00000010208 | 929 | |
| CLCA2_C | Chicken | ENSGALP00000010207 | 930 | |
| CLCA1a_Ci | *Ciona* | ENSCINP00000006772 | 674 | Program WISE2 was applied |
| CLCA1b_Ci | *Ciona* | ENSCINP00000006764 | 1075 | |
| **EC 6.4.1.1 (PYC, pyruvate carboxylase)** | | | | |
| PYC_H | Human | ENSP00000347900 | 1178 | |
| PYC_T | *Tetraodon* | GSTENP00006675001 | 462 | Partial |
| PYC_M | Medaka | ENSORLP00000006680 | 719 | Partial |

**Appendix (continued).**

| Gene name | Species | Ensembl ID | Length (aa) | Notes |
|---|---|---|---|---|
| PYC_G | *Gasterosteus* | ENSGACP00000026084 | 1179 | |
| PYC_X | *Xenopus* | ENSXETP00000018435 | 1179 | |
| PYC_C | Chicken | ENSGALP00000035230 | 167 | Partial |
| PYCa_Z | Zebrafish | ENSDARP00000001575 | 1181 | |
| PYCb_Z | Zebrafish | ENSDARP00000045088 | 1181 | |
| PYC_Ci | *Ciona* | ENSCINP00000015427 | 1154 | |
| PYC_D | *Drosophila* | CG1516-PI | 1197 | |
| **EC 4.1.1.32 (PCK, phosphoenolpyruvate carboxykinase 1, soluble)** | | | | |
| PCK1_H | Human | ENSP00000319814 | 622 | |
| PCK2_H | Human | ENSP00000216780 | 640 | |
| PCK1_T | *Tetraodon* | GSTENP00032465001 | 611 | |
| PCK2_T | *Tetraodon* | GSTENP00009627001 | 638 | |
| PCK1_M | Medaka | ENSORLP00000016033 | 623 | |
| PCK2_M | Medaka | ENSORLP00000007893 | 646 | |
| PCK1_G | *Gasterosteus* | ENSGACP00000004885 | 627 | |
| PCK2_G | *Gasterosteus* | ENSGACP00000002900 | 635 | |
| PCK1_Z | Zebrafish | ENSDARP00000018261 | 630 | |
| PCK2_Z | Zebrafish | ENSDARP00000005448 | 636 | |
| PCK1_X | *Xenopus* | ENSXETP00000024598 | 624 | |
| PCK2_X | *Xenopus* | ENSXETP00000031961 | 643 | |
| PCK1_C | Chicken | ENSGALP00000012342 | 622 | |
| PCK_Ci | *Ciona* | ENSCINP00000012511 | 382 | Partial; phylogenetically unsorted |
| PCKa_D | *Drosophila* | CG10924-PA | 638 | Phylogenetically ambiguous |
| PCKb_D | *Drosophila* | CG17725-PA | 647 | Phylogenetically ambiguous |
| **EC 1.1.1.37 (MDH, malate dehydrogenase, NAD, soluble)** | | | | |
| MDH1_H | Human | ENSP00000233114 | 334 | |
| MDH2_H | Human | ENSP00000327070 | 338 | |
| MDH1a_T | *Tetraodon* | GSTENP00018705001 | 301 | |
| MDH1b_T | *Tetraodon* | GSTENP00003840001 | 393 | |
| MDH2_T | *Tetraodon* | GSTENP00035367001 | 337 | |
| MDH1a_M | Medaka | ENSORLP00000003135 | 335 | |
| MDH1b_M | Medaka | ENSORLP00000003630 | 350 | |
| MDH2_M | Medaka | ENSORLP00000007226 | 337 | |
| MDH1a_G | *Gasterosteus* | ENSGACP00000001268 | 340 | |
| MDH1b_G | *Gasterosteus* | ENSGACP00000025915 | 333 | |
| MDH2_G | *Gasterosteus* | ENSGACP00000008052 | 337 | |
| MDH1a_Z | Zebrafish | ENSDARP00000015862 | 326 | |
| MDH1b_Z | Zebrafish | ENSDARP00000023121 | 333 | |
| MDH1_X | *Xenopus* | ENSXETP00000028520 | 333 | |
| MDH2_X | *Xenopus* | ENSXETP00000019672 | 338 | |
| MDH1_C | Chicken | ENSGALP00000014374 | 334 | |

**Appendix (continued).**

| Gene name | Species | Ensembl ID | Length (aa) | Notes |
|---|---|---|---|---|
| MDH2_C | Chicken | ENSGALP00000003012 | 337 | |
| MDH1_Ci | *Ciona* | ENSCSAVP00000018939 | 346 | |
| MDH2_Ci | *Ciona* | ENSCINP00000017905 | 345 | |
| MDH_D | *Drosophila* | CG5362-PA | 337 | |
| MDH2a_D | *Drosophila* | CG10748-PA | 349 | |
| MDH2b_D | *Drosophila* | CG10749-PA | 347 | |
| MDH2c_D | *Drosophila* | CG7998-PA | 336 | |
| **EC 4.2.1.2 (FH, fumarate hydratase)** | | | | |
| FH_H | Human | ENSP00000355518 | 510 | |
| FH_T | *Tetraodon* | GSTENP00028793001 | 455 | |
| FH_M | Medaka | ENSORLP00000021207 | 499 | |
| FH_G | *Gasterosteus* | ENSGACP00000015073 | 494 | |
| FH_X | *Xenopus* | ENSXETP00000014278 | 450 | Phylogenetically ambiguous |
| FH_C | Chicken | ENSGALP00000021472 | 507 | |
| FH_Z | Zebrafish | ENSDARP00000090090 | 425 | Phylogenetically ambiguous |
| FH_Ci | *Ciona* | ENSCINP00000016516 | 491 | |
| FH1_D | *Drosophila* | CG31874-PA | 484 | Phylogenetically ambiguous |
| FH2_D | *Drosophila* | CG4094-PA | 495 | Phylogenetically ambiguous |
| FH3_D | *Drosophila* | CG4095-PA | 503 | Phylogenetically ambiguous |
| FH4_D | *Drosophila* | CG6140-PA | 470 | Phylogenetically ambiguous |
| **EC 1.3.5.1 (SDH, succinate dehydrogenase complex)** | | | | |
| SDHA_H | Human | ENSP00000264932 | 664 | |
| SDHA_T | *Tetraodon* | GSTENP00035332001 | 696 | |
| SDHA_M | Medaka | ENSORLP00000007754 | 676 | |
| SDHA_G | *Gasterosteus* | ENSGACP00000007705 | 662 | |
| SDHA_Z | Zebrafish | ENSDARP00000076193 | 661 | |
| SDHA_X | *Xenopus* | ENSXETP00000050574 | 664 | |
| SDHA_C | Chicken | ENSGALP00000021475 | 643 | |
| SDHA_Ci | *Ciona* | ENSCINP00000008911 | 681 | |
| Scsfp_D | *Drosophila* | CG17246-PA | 661 | |
| CG5718_D | *Drosophila* | CG5718-PA | 651 | |
| SDHB_H | Human | ENSP00000364649 | 280 | |
| SDHBa_T | *Tetraodon* | GSTENP00020587001 | 281 | |
| SDHBb_T | *Tetraodon* | GSTENP00027350001 | 282 | |
| SDHBa_M | Medaka | ENSORLP00000000328 | 280 | |
| SDHBb_M | Medaka | ENSORLP00000022930 | 283 | |
| SDHBa_G | *Gasterosteus* | ENSGACP00000006746 | 281 | |
| SDHBb_G | *Gasterosteus* | ENSGACP00000001466 | 288 | |
| SDHB_Z | Zebrafish | ENSDARESTP00000005408 | 252 | |
| SDHB_X | *Xenopus* | ENSXETP00000029442 | 284 | |
| SDHB_C | Chicken | ENSGALP00000000693 | 290 | |

**Appendix (continued).**

| Gene name | Species | Ensembl ID | Length (aa) | Notes |
|---|---|---|---|---|
| SDHB_Ci | *Ciona* | ENSCINP00000014095 | 292 | |
| SDHBa_D | *Drosophila* | CG3283-PA | 297 | |
| SDHBb_D | *Drosophila* | CG7349-PA | 437 | |
| SDHC_H | Human | ENSP00000356952 | 150 | |
| SDHD_H | Human | ENSP00000374435 | 159 | |
| SDHC_T | *Tetraodon* | GSTENP00005575001 | 129 | |
| SDHC_M | Medaka | ENSORLP00000003332 | 172 | |
| SDHD_M | Medaka | ENSORLP00000012701 | 158 | |
| SDHC_G | *Gasterosteus* | ENSGACP00000023373 | 170 | |
| SDHCa_Z | Zebrafish | ENSDARP00000056369 | 143 | |
| SDHCb_Z | Zebrafish | ENSDARP00000088976 | 169 | |
| SDHD1_Z | Zebrafish | ENSDARP00000039864 | 158 | |
| SDHD2_Z | Zebrafish | ENSDARP00000072578 | 155 | |
| SDHC_X | *Xenopus* | ENSXETP00000005600 | 167 | |
| SDHD_X | *Xenopus* | ENSXETP00000057629 | 152 | |
| SDHD_C | Chicken | ENSGALP00000012780 | 157 | |
| SDH_Ci | *Ciona* | ENSCINP00000018372 | 166 | |
| CG10219_D | *Drosophila* | CG10219-PA | 182 | |
| **EC 6.2.1.4 (SUCLG, succinate-CoA ligase, GDP-forming)** | | | | |
| SUCLGa_H | Human | ENSP00000295783 | 333 | |
| SUCLGa_T | *Tetraodon* | GSTENP00003696001 | 311 | |
| SUCLGa_M | Medaka | ENSORLP00000007663 | 324 | |
| SUCLGa_G | *Gasterosteus* | ENSGACP00000023741 | 325 | |
| SUCLGa_Z | Zebrafish | ENSDARP00000069096 | 324 | |
| SUCLGa_X | *Xenopus* | ENSXETP00000047436 | 325 | |
| SUCLGa_C | Chicken | ENSGALP00000031379 | 331 | |
| SUCLGa_CiS | *Ciona savignyi* | ENSCSAVP00000015074 | 325 | |
| Scsalpha_D | *Drosophila* | CG1065-PA | 328 | |
| CG6255_D | *Drosophila* | CG6255-PA | 342 | |
| SUCLGb1_H | Human | ENSP00000307432 | 432 | |
| SUCLGb2_H | Human | ENSP00000343752 | 431 | |
| SUCLGb_T | Tetraodon | GSTENP00021008001 | 431 | |
| SUCLGb_M | Medaka | ENSORLP00000010034 | 383 | |
| SUCLGb_G | *Gasterosteus* | ENSGACP00000012380 | 430 | |
| SUCLGb_Z | Zebrafish | ENSDARP00000081954 | 406 | |
| SUCLGb_X | *Xenopus* | ENSXETP00000005372 | 347 | |
| SUCLGb_C | Chicken | ENSGALP00000012380 | 432 | |
| SUCLGb1_Ci | *Ciona* | ENSCINP00000015047 | 432 | |
| SUCLGb2_Ci | *Ciona* | ENSCINP00000015522 | 447 | |
| Sucb_D | *Drosophila* | CG10622-PA | 416 | |

**Appendix (continued).**

| Gene name | Species | Ensembl ID | Length (aa) | Notes |
|---|---|---|---|---|
| **EC 6.2.1.5 (SUCLA2, succinate-CoA ligase, ADP-forming, beta subunit)** | | | | |
| SUCLA2_H | Human | ENSP00000367923 | 463 | |
| SUCLA2 | *Tetraodon* | --- | --- | No significant BLAST hits |
| SUCLA2_F | *Fugu* | SINFRUP00000131566 | 459 | |
| SUCLA2_M | Medaka | ENSORLP00000017625 | 467 | |
| SUCLA2_G | *Gasterosteus* | ENSGACP00000010656 | 463 | |
| SUCLA2_X | Xenopus | ENSXETP00000001184 | 460 | |
| SUCLA2_C | Chicken | ENSGALP00000027401 | 463 | |
| SUCLA2_Z | Zebrafish | ENSDARP00000021652 | 467 | |
| SUCLG2b_Ci | *Ciona* | ENSCINP00000015522 | 447 | |
| CG11963_D | *Drosophila* | CG11963-PA | 502 | |
| **EC 2.3.3.1 (CS, citrate synthase)** | | | | |
| CS_H | Human | ENSP00000342056 | 466 | |
| CS_T | *Tetraodon* | GSTENP00023547001 | 469 | |
| CS_M | Medaka | ENSORLP00000019361 | 469 | |
| CS_G | *Gasterosteus* | ENSGACP00000014392 | 469 | |
| CS_X | *Xenopus* | ENSXETP00000020990 | 468 | |
| CS_Z | Zebrafish | ENSDARP00000094021 | 468 | |
| CS_Ci | *Ciona* | ENSCINP00000014102 | 203 | Partial |
| CS_D | *Drosophila* | CG3861-PB | 522 | |
| **EC 2.3.3.8 (ACLY, ATP citrate lyase)** | | | | |
| ACLY_H | Human | ENSP00000253792 | 1101 | |
| ACLYa_T | *Tetraodon* | GSTENP00012949001 | 615 | Partial |
| ACLYb_T | *Tetraodon* | GSTENP00012625001 | 962 | |
| ACLYa_M | Medaka | ENSORLP00000004628 | 1103 | |
| ACLYb_M | Medaka | ENSORLP00000017406 | 1095 | |
| ACLYa_G | *Gasterosteus* | ENSGACP00000011639 | 1092 | |
| ACLYb_G | *Gasterosteus* | ENSGACP00000008249 | 1093 | |
| ACLYa_Z | Zebrafish | ENSDARP00000044161 | 1062 | |
| ACLYb_Z | Zebrafish | ENSDARP00000072500 | 1093 | |
| ACLY_X | *Xenopus* | ENSXETP00000004892 | 1102 | |
| ACLY_C | Chicken | ENSGALP00000005492 | 1100 | |
| ACLY_Ci | *Ciona* | ENSCINP00000018161 | 1100 | Phylogenetically ambiguous |
| ATPCL_D | *Drosophila* | CG8322-PA | 1086 | Phylogenetically ambiguous |
| **EC 4.1.3.6 (CLYBL, citrate lyase beta like)** | | | | |
| CLYBL_H | Human | ENSP00000316738 | 340 | |
| CLYBL_T | *Tetraodon* | GSTENP00027917001 | 261 | Program WISE2 was applied |
| CLYBL_M | Medaka | ENSORLP00000013986 | 340 | |
| CLYBL_G | *Gasterosteus* | ENSGACP00000002457 | 261 | Program WISE2 was applied |
| CLYBL_Z | Zebrafish | ENSDARP00000091837 | 343 | |

**Appendix (continued).**

| Gene name | Species | Ensembl ID | Length (aa) | Notes |
|---|---|---|---|---|
| CLYBL_X | *Xenopus* | ENSXETP00000040951 | 380 | |
| CLYBL_C | Chicken | ENSGALP00000027213 | 324 | Program WISE2 was applied |
| CLYBL_Ci | *Ciona* | ENSCINP00000011879 | 356 | |
| **EC 4.2.1.3 (ACO, IREB, aconitase)** | | | | |
| ACO1_H | Human | ENSP00000369255 | 889 | |
| ACO1_T | Tetraodon | GSTENP00009462001 | 894 | |
| ACO1_M | Medaka | ENSORLP00000025589 | 912 | |
| ACO1_G | *Gasterosteus* | ENSGACP00000002197 | 894 | |
| ACO1_X | *Xenopus* | ENSXETP00000020189 | 893 | |
| ACO1_Z | Zebrafish | ENSDARP00000013136 | 494 | |
| ACO1_C | Chicken | ENSGALP00000003381 | 889 | |
| ACOa_Ci | *Ciona* | ENSCINP00000019424 | 888 | |
| Irp1A_D | *Drosophila* | CG4900-PA | 902 | |
| Irp1B_D | *Drosophila* | CG6342-PA | 899 | |
| ACO2_H | Human | ENSP00000216254 | 780 | |
| ACO2_T | *Tetraodon* | GSTENP00024238001 | 801 | |
| ACO2_M | Medaka | ENSORLP00000003264 | 846 | |
| ACO2a_G | *Gasterosteus* | ENSGACP00000006295 | 785 | |
| ACO2b_G | *Gasterosteus* | ENSGACP00000010099 | 779 | |
| ACO2_Z | Zebrafish | ENSDARP00000028117 | 782 | |
| ACO2_X | *Xenopus* | ENSXETP00000015081 | 760 | |
| ACO2_C | Chicken | ENSGALP00000019468 | 785 | |
| ACOb_Ci | *Ciona* | ENSCINP00000018216 | 793 | |
| CG4706_D | *Drosophila* | CG4706-PA | 783 | |
| Acon_D | *Drosophila* | CG9244-PB | 787 | |
| **EC 1.1.1.42 (IDH1, isocitrate dehydrogenase 1 (NADP+), soluble)** | | | | |
| IDH1_H | Human | ENSP00000260985 | 414 | |
| IDH2_H | Human | ENSP00000331897 | 452 | |
| IDH1_T | *Tetraodon* | GSTENP00021781001 | 410 | |
| IDH2_T | *Tetraodon* | GSTENP00035635001 | 415 | |
| IDH3_T | *Tetraodon* | GSTENP00021458001 | 438 | |
| IDH1_M | Medaka | ENSORLP00000024665 | 383 | Partial |
| IDH2_M | Medaka | ENSORLP00000015781 | 453 | |
| IDH1_G | *Gasterosteus* | ENSGACP00000012105 | 417 | |
| IDH2_G | *Gasterosteus* | ENSGACP00000021754 | 454 | |
| IDH3_G | *Gasterosteus* | ENSGACP00000014348 | 452 | |
| IDH1_Z | Zebrafish | ENSDARP00000092112 | 429 | |
| IDH1_X | *Xenopus* | ENSXETP00000017597 | 415 | |
| IDH1_C | Chicken | ENSGALP00000014317 | 418 | |
| IDHa_Ci | *Ciona* | ENSCINP00000006115 | 414 | |
| IDHb_Ci | *Ciona* | ENSCINP00000017691 | 446 | |

**Appendix (continued).**

| Gene name | Species | Ensembl ID | Length (aa) | Notes |
|---|---|---|---|---|
| Idh_D | *Drosophila* | CG7176-PC | 469 | |
| **EC 1.1.1.41 (IDH3A, isocitrate dehydrogenase 3 [NAD+])** | | | | |
| IDH3A_H | Human | ENSP00000299518 | 366 | |
| IDH3B_H | Human | ENSP00000370223 | 385 | |
| IDH3G_H | Human | ENSP00000217901 | 393 | |
| IDH3A_T | *Tetraodon* | GSTENP00015984001 | 366 | |
| IDH3B_T | *Tetraodon* | GSTENP00020960001 | 357 | |
| IDH3Ga_T | *Tetraodon* | GSTENP00028116001 | 388 | |
| IDH3A_M | Medaka | ENSORLP00000023646 | 369 | |
| IDH3B_M | Medaka | ENSORLP00000010582 | 390 | |
| IDH3Ga_M | Medaka | ENSORLP00000008916 | 400 | |
| IDH3Gb_M | Medaka | ENSORLP00000020890 | 391 | |
| IDH3A_G | *Gasterosteus* | ENSGACP00000013662 | 366 | |
| IDH3Ax_G | *Gasterosteus* | ENSGACP00000023148 | 109 | Partial |
| IDH3B_G | *Gasterosteus* | ENSGACP00000012012 | 392 | |
| IDH3Ga_G | *Gasterosteus* | ENSGACP00000011512 | 384 | |
| IDH3Gb_G | *Gasterosteus* | ENSGACP00000014059 | 382 | |
| IDH3A_Z | Zebrafish | ENSDARP00000094415 | 365 | |
| IDH3B_Z | Zebrafish | ENSDARP00000065776 | 382 | |
| IDH3Ga_Z | Zebrafish | ENSDARP00000035380 | 374 | |
| IDH3Gb_Z | Zebrafish | ENSDARP00000093079 | 343 | |
| IDH3A_X | *Xenopus* | ENSXETP00000021498 | 368 | |
| IDH3B_X | *Xenopus* | ENSXETP00000032718 | 373 | |
| IDH3G_X | *Xenopus* | ENSXETP00000001625 | 393 | |
| IDH3A_C | Chicken | ENSGALP00000038881 | 360 | |
| IDH3B_C | Chicken | ENSGALP00000023363 | 385 | |
| IDH1_Ci | *Ciona* | ENSCINP00000014956 | 369 | |
| IDH2_Ci | *Ciona* | ENSCINP00000009125 | 345 | |
| IDH3_Ci | *Ciona* | ENSCINP00000015369 | 398 | |
| CG3483_D | *Drosophila* | CG3483-PA | 391 | |
| CG12233_D | *Drosophila* | CG12233-PB | 377 | |
| CG6439_D | *Drosophila* | CG6439-PA | 370 | |
| CG5028_D | *Drosophila* | CG5028-PA | 402 | |
| **EC 1.2.4.2 (OGDH, oxoglutarate (alpha-ketoglutarate) dehydrogenase, lipoamide)** | | | | |
| OGDH_H | Human | ENSP00000222673 | 1023 | |
| OGDHL_H | Human | ENSP00000363216 | 1010 | |
| OGDHa_T | *Tetraodon* | GSTENP00010753001 | 1070 | |
| OGDHb_T | *Tetraodon* | GSTENP00025361001 | 1005 | |
| OGDHL_T | *Tetraodon* | GSTENP00016462001 | 1054 | |
| OGDHb_M | Medaka | ENSORLP00000001203 | 1039 | |
| OGDHa_M | Medaka | ENSORLP00000021894 | 949 | |

**Appendix (continued).**

| Gene name | Species | Ensembl ID | Length (aa) | Notes |
|---|---|---|---|---|
| OGDHL_M | Medaka | | | |
| OGDHb_G | *Gasterosteus* | ENSGACP00000000295 | 989 | |
| OGDHa_G | *Gasterosteus* | ENSGACP00000001427 | 1017 | |
| OGDHL_G | *Gasterosteus* | ENSGACP00000010888 | 1010 | |
| OGDH_Z | Zebrafish | ENSDARP00000009474 | 1020 | |
| OGDHa1_Z | Zebrafish | ENSDARESTP00000010149 | 509 | Program WISE2 was applied |
| OGDHa2_Z | Zebrafish | ENSDARESTP00000010682 | 1019 | Program WISE2 was applied |
| OGDH_X | *Xenopus* | ENSXETP00000041195 | 1021 | |
| OGDHL_X | *Xenopus* | ENSXETP00000012378 | 1018 | |
| OGDH_C | Chicken | ENSGALP00000028857 | 635 | Program WISE2 was applied |
| OGDHL_C | Chicken | ENSGALP00000003546 | 1014 | |
| OGDH1_Ci | *Ciona* | ENSCINP00000012074 | 1010 | |
| OGDH2_Ci | *Ciona* | ENSCINP00000012122 | 694 | Partial |
| CG33791_D | *Drosophila* | CG33791-PB | 1282 | |
| CG11661_D | *Drosophila* | CG11661-PF | 1017 | |
| **EC 1.8.1.4 (DLD, dihydrolipoamide dehydrogenase)** | | | | |
| DLD_H | Human | ENSP00000205402 | 509 | |
| DLD_T | *Tetraodon* | GSTENP00007788001 | 470 | |
| DLD_M | Medaka | ENSORLP00000000566 | 397 | Partial |
| DLD_G | *Gasterosteus* | ENSGACP00000010969 | 508 | |
| DLD_X | *Xenopus* | ENSXETP00000026823 | 496 | |
| DLD_C | Chicken | ENSGALP00000012869 | 508 | |
| DLD_Z | Zebrafish | ENSDARP00000017217 | 507 | |
| DLD1_Ci | *Ciona* | ENSCINP00000016324 | 503 | |
| DLD2_Ci | *Ciona* | ENSCINP00000027133 | 503 | |
| CG7430_D | *Drosophila* | CG7430-PA | 504 | |
| **EC 2.3.1.61 (DLST, dihydrolipoamide S-succinyltransferase, E2 component of 2-oxo-glutarate complex)** | | | | |
| DLST_H | Human | ENSP00000335304 | 453 | |
| DLST_1_T | *Tetraodon* | GSTENP00032331001 | 461 | |
| DLST_2_T | *Tetraodon* | GSTENP00032333001 | 417 | |
| DLST_M | Medaka | ENSORLP00000020806 | 465 | |
| DLST_1_G | *Gasterosteus* | ENSGACP00000009989 | 460 | |
| DLST_2_G | *Gasterosteus* | ENSGACP00000013932 | 450 | |
| DLST_X | *Xenopus* | ENSXETP00000029054 | 453 | |
| DLST_C | Chicken | ENSGALP00000016718 | 461 | |
| DLST_Z | Zebrafish | ENSDARP00000006973 | 458 | |
| DLST_Ci | *Ciona* | ENSCINP00000014023 | 449 | |
| DLST_D | *Drosophila* | CG5214-PA | 468 | |
